# Therapeutic Potentials of Antiviral Plants Used in Traditional African Medicine With COVID-19 in Focus: A Nigerian Perspective

**DOI:** 10.3389/fphar.2021.596855

**Published:** 2021-04-26

**Authors:** Alfred Francis Attah, Adeshola Adebayo Fagbemi, Olujide Olubiyi, Hannah Dada-Adegbola, Akinseinde Oluwadotun, Anthony Elujoba, Chinedum Peace Babalola

**Affiliations:** ^1^ Department of Pharmacognosy and Drug Development, Faculty of Pharmaceutical Sciences, University of Ilorin, Ilorin, Nigeria; ^2^ Department of Pharmaceutical Chemistry, Faculty of Pharmacy, University of Ibadan, Ibadan, Nigeria; ^3^ Department of Pharmaceutical Chemistry, Obafemi Awolowo University, Ile-Ife, Nigeria; ^4^ Institute of Biological Information Processing, Structural Biochemistry (IBI-7), Forschungszentrum Jülich, Jülich, Germany; ^5^ Department of Medical Microbiology and Parasitology, College of Medicine, University of Ibadan, Ibadan, Nigeria; ^6^ Nestle Nigeria Plc, Ilupeju Avenue, Lagos, Nigeria; ^7^ Department of Pharmacognosy, Faculty of Pharmacy, Obafemi Awolowo University, Ile-Ife, Nigeria; ^8^ Centre for Drug Discovery, Development and Production, University of Ibadan, Ibadan, Nigeria; ^9^ College of Basic Medical Sciences, Chrisland University, Abeokuta, Nigeria

**Keywords:** COVID-19, phytomedicines, Traditional African Medicine, herbal immuno-stimulants, herb-drug interaction

## Abstract

The coronavirus disease 2019 (COVID-19) pandemic is caused by an infectious novel strain of coronavirus known as severe acute respiratory syndrome coronavirus 2 (SARS-CoV-2) which was earlier referred to as 2019-nCoV. The respiratory disease is the most consequential global public health crisis of the 21st century whose level of negative impact increasingly experienced globally has not been recorded since World War II. Up till now, there has been no specific globally authorized antiviral drug, vaccines, supplement or herbal remedy available for the treatment of this lethal disease except preventive measures, supportive care and non-specific treatment options adopted in different countries via divergent approaches to halt the pandemic. However, many of these interventions have been documented to show some level of success particularly the Traditional Chinese Medicine while there is paucity of well reported studies on the impact of the widely embraced Traditional African Medicines (TAM) adopted so far for the prevention, management and treatment of COVID-19. We carried out a detailed review of publicly available data, information and claims on the potentials of indigenous plants used in Sub-Saharan Africa as antiviral remedies with potentials for the prevention and management of COVID-19. In this review, we have provided a holistic report on evidence-based antiviral and promising anti-SARS-CoV-2 properties of African medicinal plants based on *in silico* evidence, *in vitro assays* and *in vivo* experiments alongside the available data on their mechanistic pharmacology. In addition, we have unveiled knowledge gaps, provided an update on the effort of African Scientific community toward demystifying the dreadful SARS-CoV-2 micro-enemy of man and have documented popular anti-COVID-19 herbal claims emanating from the continent for the management of COVID-19 while the risk potentials of herb-drug interaction of antiviral phytomedicines when used in combination with orthodox drugs have also been highlighted. This review exercise may lend enough credence to the potential value of African medicinal plants as possible leads in anti-COVID-19 drug discovery through research and development.

## Introduction

**FIGURE 1:**
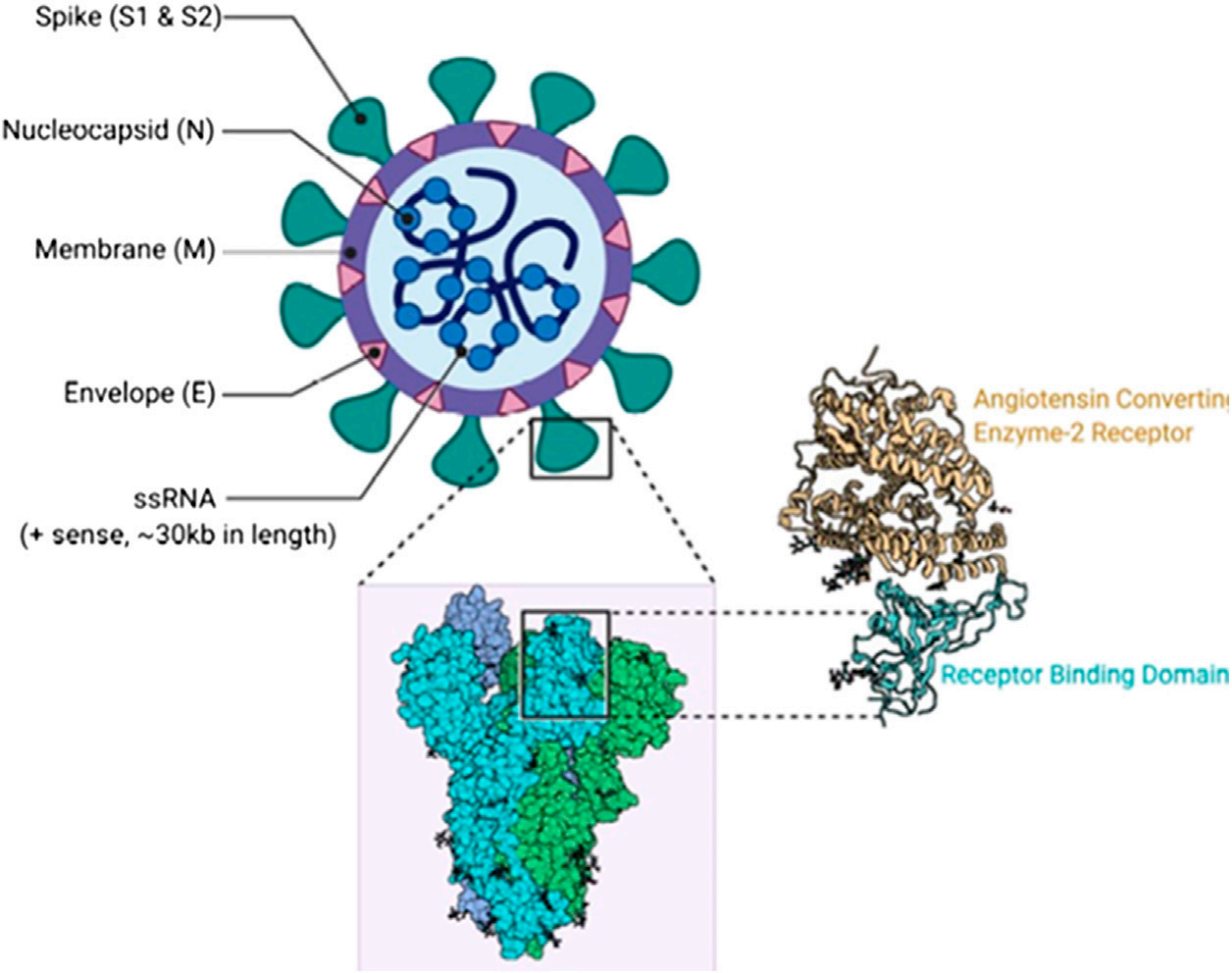
SARS- CoV two Structure (Cascella et al., 2020). Contributed by Rohan Bir Singh, MD; Made with Biorender.com.

**FIGURE 2:**
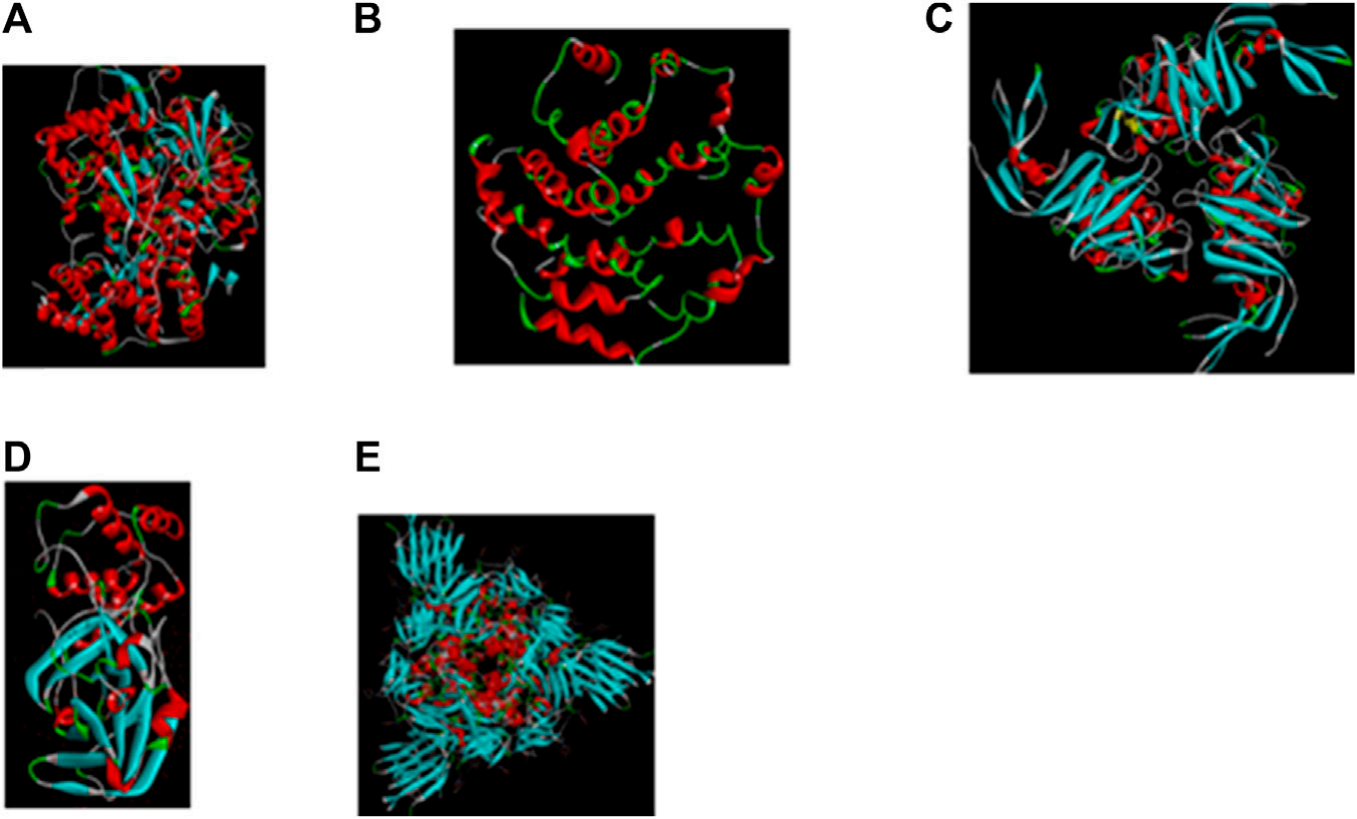
Important molecular targets in SARS-CoV2 structure for interaction with antiviral compounds in phytomedicines. Many African herbal solutions are polyherbal with potentials for more than one therapeutic targets on the viral particle **(A)** PDB 6M71: Structure of the RNA-dependent RNA polymerase from COVID-19 virus (Gao et al., 2020); **(B)** PDB 5X29: NMR structure of the SARS Coronavirus E protein pentameric ion channel (Surya et al., 2018); **(C)** PDB 6W9C: The crystal structure of papain-like protease of SARS CoV-2 (Walls et al., 2020); **(D)** PDB 6MQ: SARS-CoV-2 3CL protease (3CL pro) apo structure (Su et al., 2010); **(E)** PDB 6VXX: Structure of the SARS-CoV-2 spike glycoprotein (closed state) (Osipiuk et al., 2020).

The current pandemic threatening the global community, a highly communicable viral infection otherwise known as Coronavirus disease 2019 (COVID-19), is caused by the Severe Acute Respiratory Syndrome Coronavirus two or SARS-CoV-2 (Figures 1, 2) (Chan et al., 2020a). The sudden emergence of the disease was first noticed in Wuhan city, China, East Asia (Chan et al., 2020b; Guo et al., 2020). Social distancing, hand washing, alcoholic disinfectants or hand sanitizers, isolation/quarantine, travel restrictions, wearing of face mask, community containments and partial or total lockdown (World Health Organization, 2020) have continued to remain effective non-pharmaceutical preventive measures.

Despite all the divergent efforts to halt the spread and mortalities associated with COVID-19, the devastating micro-enemy has continued to spread causing more deaths and a lot of socio-economic implications. While most of the affected countries in Europe and America are relying solely on orthodox drugs, South-East Asia and in particular, China where the COVID-19 pandemic appear to have originated, has adequate documentation of successful outcomes following the integration of Traditional Chinese Medicine (TCM) with orthodox medicines in COVID-19 management (Chang et al., 2020; Gao et al., 2020). Interestingly, overwhelming literature evidence suggests that China and neighboring Asian territories practice a robust age-long traditional medicine system that has been favorably integrated with the western medicine; the TCM-western system of healthcare was therefore adopted to combat the earlier outbreak of SARS-CoV in Guangdong, China in 2002 leading to the reported defeat of the epidemic (Leung, 2007). Top among the well documented herbal recipes and formulations used as adjuvants alongside western medicines during the time included San Ren Tang, Yin Qiao San, Ma Xing Shi Gan Tang, Gan Lu Xiao Du Dan, and Qing Ying Tang, a polyherbal formulation containing many indigenous plants. In addition, Hong Kong has documented the traditional application of Sang Ju Yin and Yu Ping Feng San, *Isatis tinctoria* L. (Brassicaceae) and *Scutellaria baicalensis* Georgi (Lamiaceae), for prophylactic use among health workers against SARS-CoV infection (Hensel et al., 2020; Luo et al., 2020). Following the reported success with the use of herbal adjuvants during the previous outbreaks of viral infections in China, the outbreak of SARS-CoV2 received an immediate authorization of integral Traditional Chinese–Western medicines to treat COVID-19 (Gao et al., 2020). This means Traditional Chinese Medicine - TCMs (mainly plant-based) were co-administered with western drugs as adjuvants.

However, in Africa, the use of phytomedicines which is also referred to as herbal medicine or phytotherapy is well embraced in different Pan African territories where 80–90% of its rural populations rely on traditional medicines (mainly plant-based) for primary healthcare (Elujoba et al., 2005; Mahomoodally, 2013). The extensive use of the predominantly plant-derived traditional medicine in Africa otherwise referred to as Traditional African Medicine, has been described to be associated with African socio-economic and socio-cultural endowments (Elujoba et al., 2005). For this reason, the WHO has continued to sensitize African Member states toward the integration of TAM into their health system (Mahomoodally, 2013) as the body recognizes the relevance of traditional, complementary and alternative medicine to Africa which has a long history of TAM and knowledgeable indigenous practitioners. For instance, there has been an unprecedented use of phytomedicines in Africa following the outbreak and global spread of COVID-19 pandemic, a situation which has been compounded by lack of authorized medicines that are effective, affordable and accessible to the populations coupled with a relatively weak African health sector (Lone and Ahmad, 2020; WHO, 2020). Coincidentally, available evidence from Africa Center for disease Control and Prevention (Africa CDC) suggests that the African continent is the last to be hit by the viral pandemic and least affected continent whose mortality rate (2.1%) until July 21, 2020 was less than half of the reported global mortality (5%) rate. Hence, despite the vulnerability of the African continent, it accounts for only 5% of the globally reported cases of COVID-19. While several factors may be attributable to this seeming positive trend, the near absolute dependence on the obvious potentials of the African medicinal plants for COVID-19 management may not be ruled out. As a malaria endemic region, the Sub-Saharan Africa often co-administer herbal remedies alone or combined with orthodox drugs as adjuvants and many of these plant-based medicines have since been informally repurposed by various users for COVID-19 prevention and symptomatic management as simple home remedies. Unlike the Traditional Chinese Medicine, there is a paucity of well reported studies on the impact of the widely embraced TAM adopted so far for the prevention, management and treatment of COVID-19. This review is therefore aimed at the documentation of African medicinal plants and their therapeutic potentials in the prevention and management of COVID-19. The potential risks associated with herb-drug interaction of antiviral phytomedicines when used in combination with orthodox drugs have been highlighted. In addition, we document the pharmacokinetic considerations in developing potential anti-COVID19 herbal products.

## Methods

In this review, a literature search was carried out and popular scientific databases including PubMed, PubChem, Google Scholar, HINARI; these were searched to retrieve scientific peer-reviewed publications on African traditional medicinal plants with antiviral potentials. Considering the framework of unveiling the role played by antiviral plants commonly used in Traditional African Medicine (TAM) in tackling deadly infectious diseases such as COVID 19, the traditional uses, bioactive metabolites, in silico, *in vitro*, *in vivo*, and clinical studies as well as the sustainable use of these plants in African ethnomedicine and associated challenges were considered and included. Articles published in English before July 2020 using the keywords; “Africa”, “antiviral plants”, “SARS COV”, “COVID-19”, “antiviral phytomedicines”, “Traditional African Medicine”, “herbal immuno-stimulants”, “herb-drug interaction” were subsequently retrieved. Generally accepted and popular anecdotal claims on plant-based COVID-19 treatment options have also been included wherever appropriate. Excluded from this review were studies carried out on plants not found in Africa, repetitive studies and publications that have failed to meet the inclusion criteria. Following the minimal impact of the much earlier outbreaks of the severe acute respiratory syndrome (SARS) and Middle East respiratory syndrome (MERS) epidemics on the African continent, SARS and MERS have not attracted a significant TAM-related research attention; and therefore are not a focus of this review.

In order to rightly place the claims made in proper context with regards to the availability of research data, we have defined and categorized the claims reported in this review based on the relevance of different plants and plant products in COVID-19 management; consequently, to reveal what level of evidence exists for a reported plant, the following classifications have been described;

Level I evidence - Evidence from at least one clinical study.
Level II evidence - Inferences supported by *in vivo* experiments.
Level III evidence - Detailed mechanistic and other *in vitro* evaluations support the conclusion.
Level IV evidence - Evidence from preliminary *in vitro* screening or in silico data (IV*).
Level V evidence - Claims are extrapolated from activities demonstrated against other similar viruses or in contextually related settings.

These defined levels of evidence are indicated in square brackets within the review, e.g [Level I] for claims derived from at least one human study.

### Medicinal Plants of African Origin with Antiviral Activities

Africa, with one of the richest cultural heritage in the traditional application of plants in healthcare, is endowed with a vast plant biodiversity (Cunningham, 1997; Dzoyem et al., 2013). An estimated 68,000 plant species have been reported to grow within the continent, over half (35,000) of which are endemic to Africa (UNEP-WCMC, 2016).

The peculiar diversity and uniqueness of climatic, soil, rainfall and environmental factors have encouraged the growth of an extensive plant diversity, endemism and great variation in indigenous plants across the entire region (James et al., 2007). The proximity, accessibility and abundance of African medicinal plant resources may have informed their amazing acceptability and popularity by African populations for meeting primary healthcare needs (Neuwinger, 2000) especially during emergency scenarios as in COVID-19.

**FIGURE 3:**
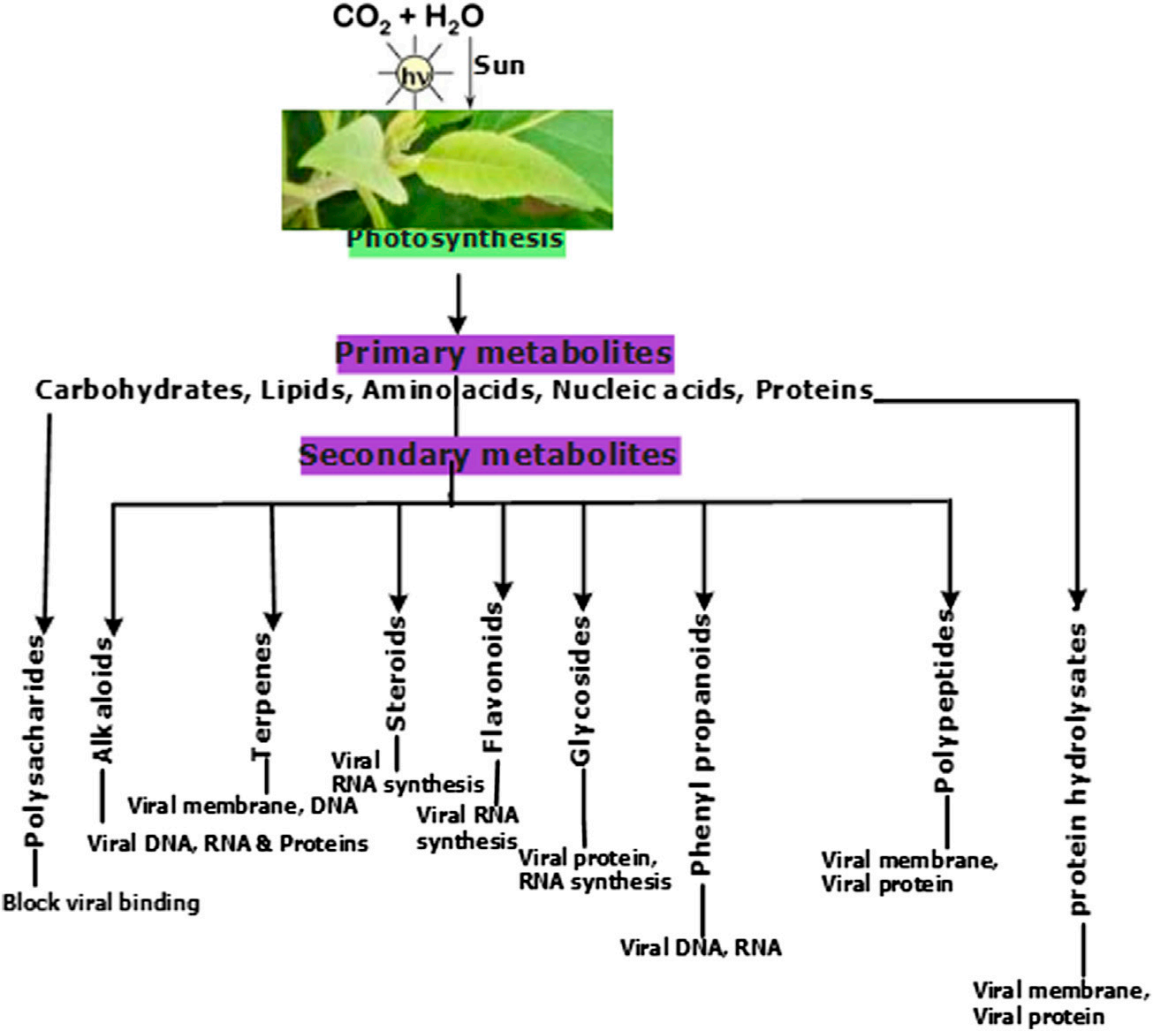
The role of primary and secondary plant metabolites as antiviral agents.

**FIGURE 4:**
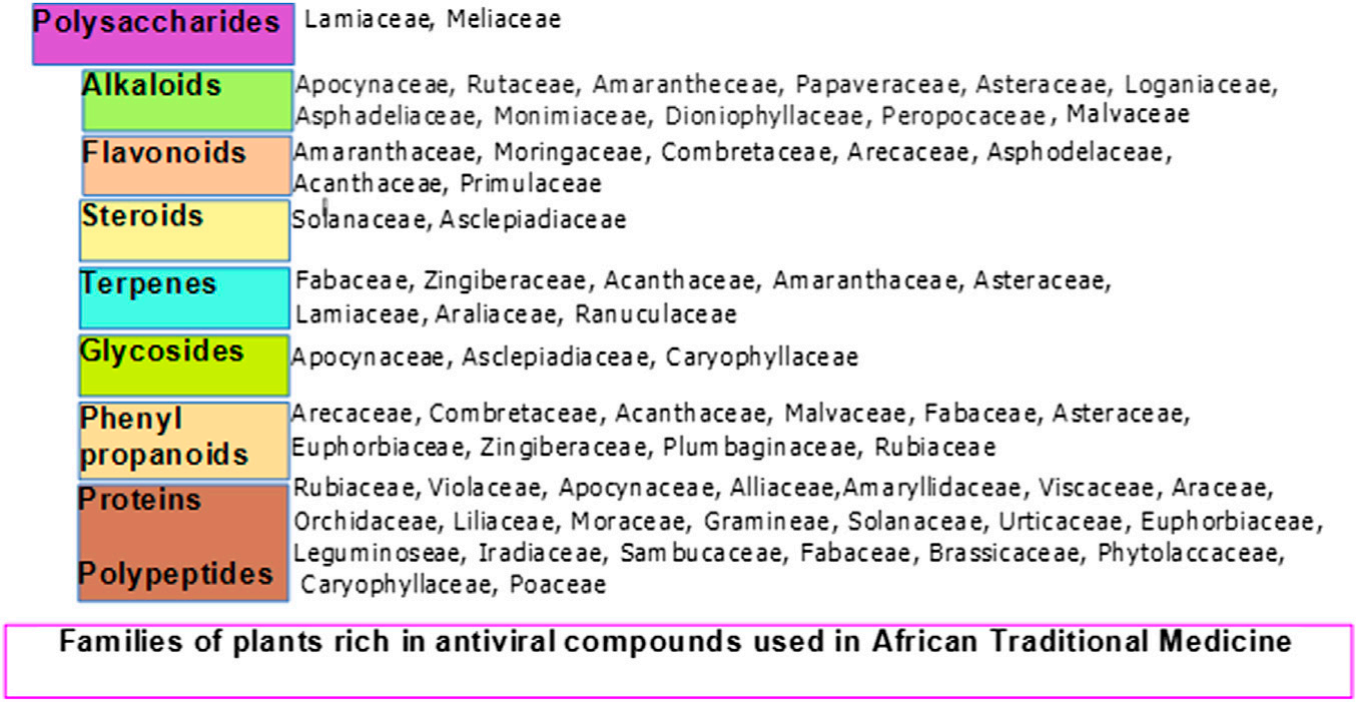
Family of Plants endemic to Africa expressing and accumulating antiviral primary and secondary metabolites.

Diverse plants, with their isolated products and derivatives with antiviral properties including alkaloids, flavonoids, phenolic compounds, terpenes, polysaccharides and polypeptides (Figure 3), have been reported (Badia-Boungou et al., 2019; Maroyi 2014). As nature’s *biological* laboratories containing hundreds and thousands of bioactive metabolites, African medicinal plants abundantly accumulate phytochemical markers and defense compounds of chemotaxonomic significance in different plant families (Figure 4). This variation in bioactive chemical markers in different plants has facilitated and justified the use of some plants in some families more often than others following their superior efficacy for conditions they are meant to treat in Traditional African Medicine (TAM) including viral outbreaks. Plant families which accumulate antiviral classes of compounds have been summarized in Figure 4. The antiviral properties and immuno-modulatory activities of these compounds can be utilized in the prevention, treatment and management of COVID-19, which till date awaits effective, safe, affordable and accessible treatment options. The efficacy of some plants and derived phytochemicals of African origin have been established following their potential to interfere with the replication and transcription machinery of some causative agents of viral infections (Mehrbod et al., 2018a; Mehrbod et al., 2019). Documented antiviral potency of these medicinal plant extracts justifies their selection for further studies as potential agents for prophylactic administration or potential therapeutic intervention against COVID-19. However, an in-depth and rigorous analysis of their efficacy and safety using internationally acceptable protocols is germane during clinical trials prior to healthcare utilization.

Cos and colleagues (Cos et al., 2002a) reported some African plants that are active against poliomyelitis, coxsackie, semliki forest, measles, and vesicular stomatitis virus (VSV). The antiviral activity of the extracts investigated was determined as the reduction factor (RF) of the viral titer which is interpreted as the ratio of the virus titer in the absence and in the presence of the extract. The leaves of *Macaranga kilimandscharica* Pax (Euphorbiaceae) exhibited considerable *in vitro* effect against measles. The 80% ethanol extracts were found to block the viral replication of Coxsackie and Measles. The leaf extracts of *Guizotia scabra (Vis.)* Chiov. (Asteraceae) were active against Coxsackie and Polio, while *Pavetta ternifolia* Hiern (Rubiaceae) (leaves) was shown to display high activity against only Coxsackie. The leaves of *Eriosema montanum* Baker f. (Fabaceae) have been reported to have considerable activity against all the tested viruses. The stems of *Entada abyssinica (EnE)* Steud. ex A. Rich (Fabaceae) were highly potent against Polio while displaying intermediate efficacy against other viruses tested with the exception of Measles. The leaves of *EnE* have profound antiviral effects against Semliki forest virus (Cos et al., 2002a). The antiviral activity of aqueous extract of *Syzygium brazzavillense* Aubrév. and Pellegr. (Myrtaceae) against coxsackievirus (CV) and poliovirus type 1 was revealed by Badia- Boungou et al. The extract was found to inhibit replication of CVB4 in HEp-2 cell cultures and also limit the cytopathic effect (CPE) induced by type 1 polioviruses and by CVB2, CVB3, and CVB4 (Badia-Boungou et al., 2019) and may possibly interfere with SARS-CoV-2 replication. Active fractions and metabolites (such as flavonoids and terpenenoids) reported in these African plants have now been documented to be promising COVID-19 (da Silva Antonio et al., 2020; James et al., 2007; Nworu et al., 2017) remedies making these plants sustainable biomass for drug discovery against COVID-19 [Level V]. However, little is known about the toxicity of the plants while *in vivo* data as well as elaborate and mechanistic *in vitro* investigations will be required to support the current preliminary *in vitro* findings.

In another experiment, Cos et al. (Cos et al., 2002b) investigated the antiviral activity of certain Rwandan plants against human immunodeficiency virus type-1. They showed that ethanolic extract of *Aspilia pluriseta* Schweinf. ex Engl. (Asteraceae) exhibited pronounced antiviral activity by enabling an absolute cell-resilient against HIV-induced cytopathic effect compared to the controls. The selective index value of the extract was found to be greater than 12 (Cos et al., 2002b). Also, thiarubrine-A **(93)** isolated from the leaves of *A. pluriseta* demonstrated phototoxic activity against enveloped viruses such as cytomegalovirus and Sindbis virus (Hudson et al., 1986). The ethanolic extract of *Rumex nepalensis* Spreng. (Polygonaceae) with a selective index of 11 was able to achieve 89% cell protection against HIV-induced cytopathic effect. The residue obtained when ethanolic extract of *Tithonia diversifolia* (Hemsl.) A. Gray (Asteraceae) was suspended in 60% methanol and was subsequently extracted with petroleum ether and ethyl acetate concurrently: it displayed significant inhibitory effect as anti-HIV-1 agent having a selective index greater than 461 (Cos et al., 2002b). In addition, the aqueous fraction showed pronounced anti-HIV-1 activities at concentrations of 200, 40, 8, 1.6 and 0.32 μg/ml. Furthermore, isolation of sesquiterpene lactones such as diversifolin **(91),** diversifolin methyl ether, and tirotundin **(92)** from *T. diversifolia* has been reported with relevant pharmacological properties (Asres et al., 2001; Cos et al., 2002b). The mode of action of these compounds is associated with decline in the production of inflammatory mediators including cytokines and chemokines. The reported mechanism involves the interference with the DNA binding activity of the transcription factor NF-ҡB (Rüngeler et al., 1998). The fact that some of these products exhibit biological activities involving host inflammatory response may indicate their potential treatment potentials in COVID-19 with its reported inflammatory undertone [**Level III, V**]. Another bioactive compound with anti-HIV-1 activity isolated from the mature stems of *T. diversifolia* is an artemisinic acid derivative (Bordoloi et al., 1996). Artemisinic acid is a sesquiterpenoid precursor of artemisinin and the semi-synthetic product 12-*N*-butyl deoxoartemisinin has been reported to inhibit HIV activity (Jung et al., 2012). It should however be pointed out that in-depth *in vivo* and clinical investigations will need to be conducted to objectively establish the clinical relevance of these plant products. Interestingly however, the major component of the Madagascar’s COVID organics (CVO), a herbal formulation containing *Artemisia annua* L. (Asteraceae), is the antimalarial compound artemisinin. Although the efficacy and safety of CVO is yet to be clinically validated, the overwhelming willingness of other African countries to participate in the clinical trials highlights the priority accorded plant-derived medicines in Africa. The WHO and African Health Ministers have agreed to allow herbal and indigenous health products to go through requisite clinical trials to establish their efficacy and safety prior to adoption as treatment options for COVID-19 (WHO, 2020). Increasing evidence suggests that these plant-derived antimalarial sesquiterpene lactones, an active component *T. diversifolia* and *A. annua* may hold a promise in COVID-19 treatment provided further research attention is given to support efficacy and safety (Rahman et al., 2020; da Silva Antonio et al., 2020).

*Helichrysum foetidum* Moench (Asteraceae) is one of the selected Rwandan medicinal plants (Sindambiwe et al., 1999) whose ethanol extract (200 mg/ml), after a 10-fold dilution produced antiviral activity by limiting the extracellular viability of herpes simplex virus type 1 (HSV 1) and Semliki forest virus A7 (SF A7) while *Chamaecrista mimosoides* (L.) Greene (Fabaceae) and *Ipomoea involucrata* P.Beauv. (Convolvulaceae) under the same experimental conditions and concentration displayed high antiviral potential against HSV 1*. Ipomoea involucrata* was potent as a virucidal agent against vesicular stomatitis virus T2 (VSV T2), SF A7 and measles virus strain Edmonston A (MV-EA). Findings from this study showed that *C*. *mimosoides*, *Rotheca myricoides* (Hochst.) Steane and Mabb. (Lamiaceae) and *Helichrysum cymosum* (L.). D. Don ex G. Don (Asteraceae) demonstrated virucidal activity against HSV 1, measles virus strain Edmonston A (MV-EA), and Semliki forest virus A7. In addition, the study highlights the virucidal activity of *Maesa lanceolata* Forssk. (Primulaceae) against the screened enveloped viruses which was exceptional compared to the other tested plants, making this plant an interesting candidate for further research consideration against SARS-CoV-2 [**Level V**]. Also investigated is a mixture isolated from methanol extract of *M*. *lanceolata* (leaves) termed maesasaponin mixture A. This mixture was found to reduce the titer and infectivity of herpes simplex virus type 2 (HSV 2) at concentrations of 100 μg/ml and 250 μg/ml, respectively. More so, it incapacitated the virus at 500 μg/ml concentration. Maesasaponin mixture A also repressed the activity of vesicular stomatitis virus T2 (VSV T2) (Sindambiwe et al., 1999). Maesasaponin mixture A may be a promising potential source of active antiviral metabolites which may produce activity against SARS-CoV-2 following a more elaborate preclinical, clinical investigations and phytochemical standardization of extracts which are lacking in the study under review [**Level V**].

The extracts of *Pittosporum viridiflorum* Sims (Pittosporaceae) and *Rapanea melanophloeos* (L.) Mez (Primulaceae) were reported by Mehrbod et al. (Mehrbod et al., 2018a) to have inhibitory effect against influenza A virus (IAV). The activity of the extracts resulted in averages of 7.4 and five logs hemagglutination (HA) decrements for *R. melanophloeos* and *P. viridiflorum*, respectively. This shows the potency of the plants against IAV (Mehrbod et al., 2018a). In another study, Mehrbod and colleagues again evaluated the activity of a glycoside flavone **(73)** (quercetin-3-O-α-L-rhamnopyranoside) isolated from *R. melanophloeos* against IAV. Quercetin-3-O-α-L-rhamnopyranoside **(73)** was reported to decrease the virus titer at 150 μg/ml by directly inhibiting the virus replication, and modulation of cytokine production (Mehrbod et al., 2018b). Research evidence supports the antiviral activity and more specifically, anti-COVID-19 potentials of a combination of quercetin and vitamin C, some common components of the mainly polyherbal extracts used in TAM (Colunga Biancatelli et al., 2020) [**Level III**]. Interestingly, emerging evidence suggests that the anti-SARS-2 activity of glycosylated forms of flavonoids may be significantly higher than their respective aglycons while plant extracts and fractions may be significantly more effective than isolated pure compounds (Zakaryan et al., 2017; da Silva Antonio et al., 2020). However, the indigenous formulations containing these plant species require preclinical and clinical standardization for evidence-informed application and for a possible clinical use.

In a study demonstrating the antiviral activity of Ethiopian medicinal plants against both HIV-1 and HIV- 2, the methanol fraction obtained from the root bark of *Bersama abyssinica* Fresen. (Francoaceae) and the leaves of *Combretum paniculatum* Vent (Combretaceae) at median effective concentrations (EC_50_) of 3.1 and 5.2 μg/ml were the most potent in inhibiting the replication of HIV-1 having a selective index of 3.8 and 6.4, respectively [**Level V**]. The extracts obtained from the leaves of *Dodonaea viscosa subsp. angustifolia* (L.f.) J. G. West (Sapindaceae) and the stem bark of *Ximenia americana* L. (Olacaceae) were found to be slightly active against HIV-1 with EC_50_ values ranging from 8.3 to 27.7 μg/ml and selectivity indices that ranged from 3.9 to 4.9. The acetone fraction of *C. paniculatum* displayed an inhibitory potential against HIV-2 with a relatively high selectivity index of 32 at an EC_50_ value of 3.0 μg/ml while demonstrating moderate activity against HIV-1 with EC_50_ value of 15 and selectivity index of 6.4. Also, the replication in HIV-2 was altered by hydroalcohol fraction of *X. americana* at EC_50_ value of 27.1 μg/ml (Asres et al., 2001). In addition to lack of robustness and quality issues associated with these investigations, it remains to be determined if *in vitro* studies would suggest potential benefits of clinical relevance.

Another study involving human subjects, carried out in collaboration with herbal practitioners in different districts of Uganda, revealed that HIV-positive patients showed a treatment outcome with significant decrease in CD4 positive T-cell lymphocytes in the blood when treated with *Aloe spp*., *Erythrina abyssinica* Lam. (Fabaceae), *Nauclea latifolia* Sm (Rubiaceae)*, Psorospermum febrifugum* Spach (Hypericaceae), *Mangifera indica* L. (Anacardiaceae), and *Warburgia salutaris* (G. Bertol.) Chiov. (Canellaceae) (Lamorde et al., 2010)*.* The use of *Calendula officinalis* L([Asteraceae) have also been shown to result in progressive decline in viral loads and in CD4 T-cell counts in HIV-positive volunteers (Mills et al., 2005). However, these human studies lack adequate comparative data so that it remains unclear whether the patient recovered because of the use of particular herbal preparation or the general clinical care received. While findings from this study may be of interest, there is need for further investigation to establish an elaborate toxicological data, *in vivo* evidence and clinical proof of safety and efficacy. These documented antiviral African medicinal plants hold promise in the ambitious search for potent medicines to defeat the lethal COVID-19 pandemic [**Level V**].

### African Plant-Derived Antiviral Metabolites, Immunomodulation and Molecular Targets

Phytomedicnes have shown potentials as immunoadjuvants for their ability to increase the effectiveness of vaccines while plant-derived chemical compounds including ellagic acid **(80)**, curcumin **(72)**, flavonoids and quercetin possess anti-infective properties that work either by directly attacking the pathogen or indirectly by stimulating innate and acquired defense mechanisms of the host (Sodagari et al., 2018; Afolayan et al., 2020). Chemically diverse antiviral compounds including primary plant metabolites such as polysaccharides, proteins, lectins, protein hyrolysates and aminoglycans (Monzingo et al., 1993; Bouckaert et al., 1996; Sankaranarayanan et al., 1996; Harata and Muraki, 2000; Meagher et al., 2005) as well as secondary metabolites including alkaloids, phenylpropanoids, tannins, flavonoids, lignans, coumarins, glycosides, steroids, terpenes, polypeptides, antimicrobial peptides, defensins, cyclotides (**1–7**) and many other plant-derived cystine-knot peptides (Kapoor et al., 2017; Rex et al., 2018; Younas et al., 2018; Berit et al., 2020; Ghildiyal et al., 2020; He et al., 2020) have been detected and isolated from African medicinal plants **(1–172)**. The role of these antiviral compounds and their main molecular target have been presented in Figure 3 while plant families native to Africa which abundantly express and accumulate these phytocompounds that have found uses as anti-infective agents in TAM are presented in Figure 4.

Phytomedicines with a long history of use in traditional medicines and bioactive compounds obtained from them have been shown to exert antiviral, anti-inflammatory and immunomodulatory effects and these bioactivities have been proposed to be linked (Fialho et al., 2016), following their ability to modulate the immune response (Han et al., 1998; Zhang et al., 2002; Shivaprasad et al., 2006; Cruz et al., 2007; Sodagari et al., 2018) and in parallel reduce viral or parasite load (Omoregie and Pal, 2016; Michelini et al., 2018; Jasso-Miranda et al., 2019; Salinas et al., 2019; Afolayan et al., 2020). These desirable dual antiviral effects have been demonstrated in indigenous plants used in TAM for the treatment of various viral diseases (Goren et al., 2003; Esimone et al., 2005; Akram et al., 2014; Buba et al., 2016; Donma and Donma, 2020; Jacques et al., 2020; Kumar and Venkatesh, 2016; Kumar et al., 2015; Nawrot et al., 2014; Nworu et al., 2017; Osadebe and Omeje, 2007; Parvez et al., 2019; Raza et al., 2015). For instance, *Combretum micranthum* G. Don is one of the main constituents of an indigenous Nigerian antiviral phytomedicine called “Seven Keys to Power” used in the traditional management of smallpox, chicken pox, measles and HIV/AIDS (Esimone et al., 2005). In addition, *R. capparoides* has been used by herbalists in the eastern part of Nigeria for the treatment of chickenpox, smallpox and hepatitis, while *C. cajan* is used in ethnoveterinary medicine for the treatment of several viral diseases of cattles in Northern Nigeria (Esimone et al., 2005). However, rigorous, robust and well validated scientific investigations are needed to turn these potential antiviral remedies to clinical use. At this time, these data are not available, thus limiting their application.

A typical medicinal plant is a biological factory of a plethora of complex bioactive metabolites and most of the phytomedicines used in TAM are polyherbal with potential multiple targets in host and/or pathogen structure. Expectedly, complex phytotherapeutics which target both the pathogen as well as the host structure required for infection of viruses without a significant cytotoxicity to the host, could represent an alternative way to develop new and effective antiviral phytotherapies (Bekerman and Einav, 2015). Illustrated in Figures 1, 2 are some important molecular targets identified in SARS-CoV-2 and featuring druggable structural components capable of fostering interaction with nature-inspired antiviral metabolites biosynthesized from both primary and secondary metabolic pathways presented in Figure 3 (da Silva Antonio et al., 2020; Ghildiyal et al., 2020). Bioactive protein hydrolysates and cysteine-rich polypeptides target viral membrane and proteins, alkaloids and glycosides target viral proteins and RNA, terpenes target viral membrane while steroids and flavonoids target viral RNA synthesis (Figure 3). For instance, the interaction between the spike glycoproteins of SARS COV-2 and the host cell angiotensin converting enzyme 2 (ACE2) receptors which leads to viral attachment and entry, culminating in COVID-19 could be prevented or blocked effectively [**Level V**] by antiadhesive phytocompounds such as phenolics, tannins and polysaccharides (Jassim and Naji, 2003; Hensel et al., 2020) reported in some African antiviral plants including *Adansonia digitata* L., *Andrographis paniculata* (Burm.f.) Nees, *Combretum micranthum* G.Don, *Macaranga barteri* Müll. Arg*.*, *Azadirachta indica* A. Juss. (Table 1). These antiviral metabolites accumulate in high amounts in several plant families used in TAM including the Lamiaceae, Meliaceae, Asteraceae, Arecaceae, Acanthaceae, Combretaceae, Zingiberaceae, Euphorbiaceae and Malvaceae (Figure 4). However, further mechanistic studies, safety investigation as well as clinical studies are required for their clinical applications.

**TABLE 1:** Selected antiviral Angiosperm plants of African origin and the major class of phytochemicals present based on widespread use and documented evidence.

S/N	Plants	Class of phytochemicals present	Identified phytochemicals with antiviral activity	Indications	Country
1	*Achyranthes aspera* L. (Amaranthaceae)	Flavonoids, alkaloids, terpenoids Goyal et al. (2007)	Oleanolic acid **(168)** Mukherjee et al. (2013)	HSV-1	Africa, south Afrcia
				HSV-2	
				HIV- Mukherjee et al. (2013)	
**2**	*Adansonia digitata* L. (Malvaceae)	Phenolics	Nil	HSV-1	Nigeria
					Senegal Sulaiman et al. (2011)
**3**	*Andrographis paniculata* (Burm.f.) Nees (Acanthaceae)	Diterpenoids, flavonoids, polyphenols Pongtuluran and Rofaani (2015)	Andrographolide **(63) (** Pongtuluran and Rofaani (2015)	HSV-1	Nigeria Hamidi et al. (1996)
				SRV	
				EBV Wiart et al. (2005)	
				DV Panraksa et al. (2017)	
**4**	*Aspalathus linearis* (Burm.f.) R.Dahlgren (Fabaceae)	Phenolics Rahmasari et al. (2017)	Aspalathin **(105),** nothofagin **(106**), isoorientin **(104**), orientin **(103**), quercetin **(73**), luteolin **(170)** Rahmasari et al. (2017)	HIV	South Africa
				Influenza Rahmasari et al. (2017)	
**5**	*Azadirachta indica* A. Juss. (Meliaceae)	Carbohydrates	Polysaccharides P1 and P2	PV-1 Faccin-Galhardi et al. (2012)	African countries
**6**	*Bulbine frutescens* (L.) Willd. (Xanthorrhoeaceae)	Phenolics, alkaloids, flavonoids Shikalepo et al. (2018)	Myricitin **(32)**, xanthohumol **(96**), scutellarin **(95),** methoxyflavone **(169)** Shikalepo et al. (2018)	HIV-1 Shikalepo et al. (2018)	South Africa
**7**	*Canavalia ensiformis (L.) DC.* (Fabaceae)	Protein	Lectins (Concanavalin A) Figure 6	HSV Marchetti et al. (1995)	Nigeria Africa
**8**	*Cocos nucifera* L. (Arecaceae)	Phenolics Esquenazi et al. (2002)	Catechins **(133)** Esquenazi et al. (2002), myricetin **(136)** Vlietinck et al. (1997)	EBV	Kenya
		Tannins Lima et al. (2015)		CMV	
		Flavonoids Vlietinck et al. (1997)		VV Lima et al. (2015) HIV-1 Vlietinck et al. (1997)	
**9**	*^a^* *Combretum micranthum* G.Don (Combretaceae)	Phenolics, tannins Ferrea et al. (1993), Flavonoids Welch (2010)	Catechin **(133)**, catechinic acid Ferrea et al. (1993) cinnamtanins **(98),** pavetanins **(97),** AOCA(Alkaline auto-oxidized catechins) Vlietinck et al. (1997), Apigenin **(156)** Welch (2010)	HSV-1	Nigeria
				HSV-2 Ferrea et al. (1993)	
				HIV-1 Vlietinck et al. (1997)	
**10**	*Echinacea purpurea* (L.) Moench (Compositae)	Phenolics, Alkamides Vimalanathan et al. (2005)	Cichoric acid **(108)** Vimalanathan et al. (2005) Iwu (2014)	HIV Awortwe et al. (2013)	South Africa Zimbabwe
				HSV	
				Influenza Barnes et al. (2005)	
**11**	*Glycyrrhiza glabra* L. (Fabaceae)	Triterpenes (saponins), flavonoids Vlietinck et al. (1997)	Glycyrrihizin and its derivatives **(107)**, liochalchone, isolicoflavonol, glycocoumarin, glycyrrhizoflavone, licopyranocoumarin	HSV-1	South Africa
				HIV Vlietinck et al. (1997)	
**12**	*Macaranga barteri* Müll. Arg. (Euphorbiaceae)	Phenolics (stilbenes) Ogbole et al. (2018), Segun et al. (2019)	Vedehanin **(110**), schwenfurthin, mappai Ogbole et al. (2018), Segun et al. (2019)	EV^b^ Ogbole et al. (2018), Segun et al. (2019)	Nigeria
**13**	*Musa acuminata* L. Musa spp (Musaceae)	Protein	Lectins Peumans et al. (2000) Figure 7	Anti-HIV Swanson et al. (2010)	Nigeria, tropical Africa
**14**	*Oldenlandia affinis* (Roem. and schult.) DC. (Rubiaceae)	Peptides	Cyclotides (KB1, KB8) Ireland et al. (2008) Figure 5	HIV Daly et al. (2004)	Dr. Congo
**15**	*Papaver somniferum* L. (Papaveraceae)	Alkaloids Vlietinck et al. (1997)	Papaverine **(99)** Vlietinck et al. (1997)	HIV-1 Vlietinck et al. (1997)	Nigeria
**16**	*Rapanea melanophloeos* (L.) Mez (Primulaceae)	Flavonoids Mehrbod et al. (2018a)	Quercetin **(73)** Mehrbod et al. (2018a)	Influenza A Mehrbod et al. (2018a)	South Africa
**17**	*Zingiber officinale* Roscoe (Zingiberaceae)	Terpenoids Chrubasik et al. (2005), Iwu (2014)	Beta sesquiphellandrene **(109)** Chrubasik et al. (2005), Iwu (2014)	RhV	Nigeria
				RSV Chrubasik et al. (2005), Iwu (2014)	

HIV–Human Immunodeficiency Virus; HSV 1–Human Simplex Virus one; HSV 2–Human Simplex Virus two; RhV–Rhinovirus; RSV–Respiratory Syncytial Virus; EBV–Epstein-Barr Virus; CMV–Cytomegalovirus; VV–Visna Virus; DV–Dengue Virus; SRV–Simian Retrovirus; PV-1 -Poliovirus type 1.

^a^ As a part of the seven Keys preparation, it is used to treat small-pox, chicken pox and measles. Welch (2010).

^b^ It is only effective against serotypes E7 and E19. (Segun, et al., 2019); (Ogbole et al., 2018).

**FIGURE 5:**
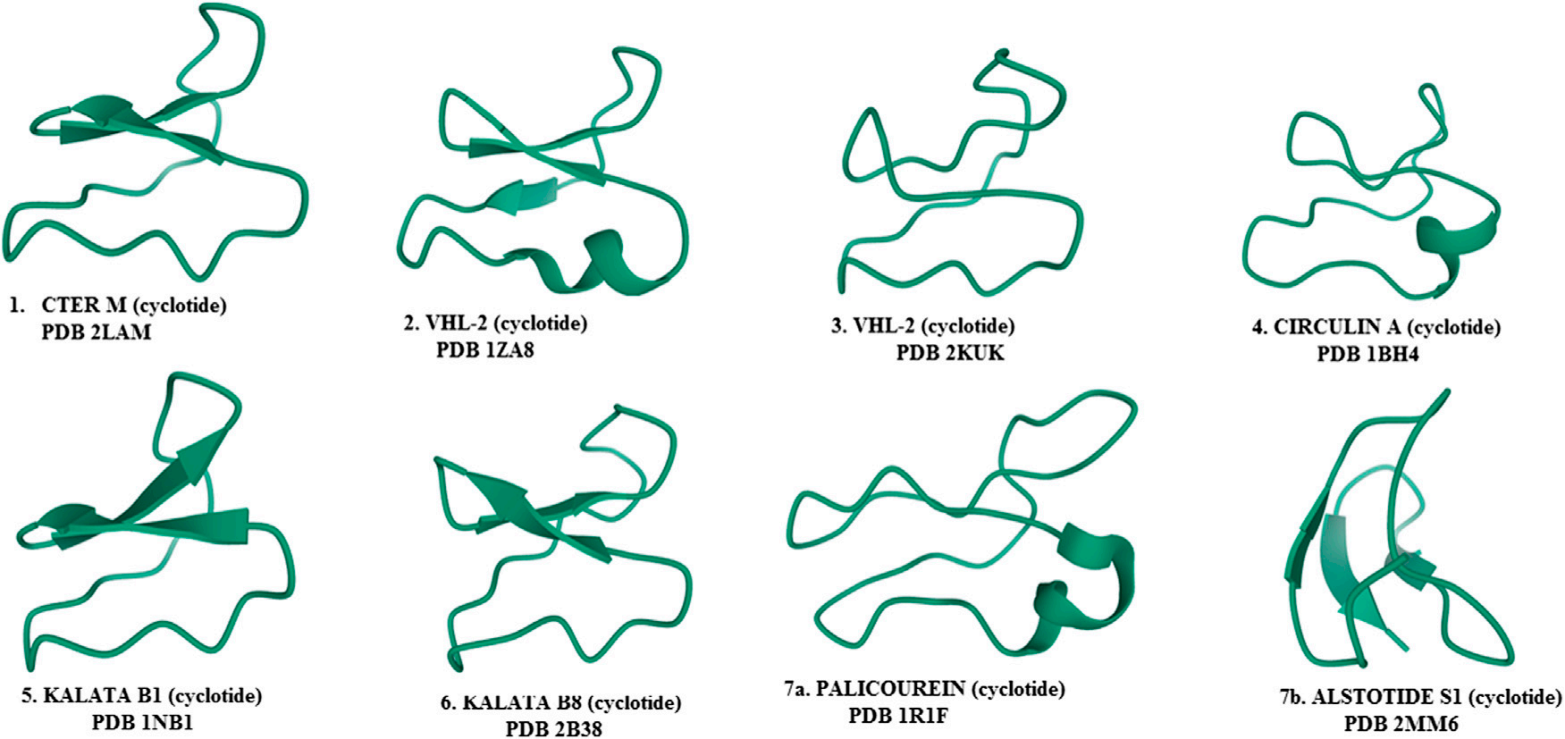
Antiviral cyclotides (cysteine-rich peptides) isolated from plants.

Plant-derived cysteine knot peptides including antimicrobial peptides and defensins whose bioactivities like other types of defensins are able to block viral infection by clustering the viral particles and blocking receptor binding (Nguyen et al., 2016; Weber, 2020). These hormone-like disulphide-stabilized peptides have been described to mediate in the inhibition of viral entry, viral particle disruption, interference with essential cell signaling or viral gene expression, or by other poorly-understood mechanisms. Furthermore, in addition to the direct antiviral activities outlined above, antimicrobial peptides and defensins modulate adaptive immune responses following their ability to attract immune cells (Weber, 2020). Cystine knot polypeptides (Figure 5) are well distributed in tropical African flora within the Apocynaceae, Rubiaceae, Violaceae, Curcubitaceae, Leguminoseae, Poaceae and Fabaceae plant families (Figure 4). Molecular studies have shown that these suites of peptides bind to viral spike and membrane proteins (Nguyen et al., 2016) and may therefore be early acting in preventing viral attachment and entry into the host cell. As some of the most exploited plant families in TAM (with the exception of Violaceae), plants species from them could help in COVID-19 treatment [**Level V**] and therefore deserve further anti-SARS CoV-2 molecular studies. Interestingly, knottin peptidyl therapeutics are stable to extreme conditions and easily extracted under aqueous mediums commonly used in TAM. Unfortunately, despite their emerging therapeutic potentials, research in cysteine knotted polypeptides has not received adequate scientific attention as less than 1% of African flora has been screened for peptide drug discovery (Attah et al., 2016a). Carbohydrate-binding lectin proteins from African Musa species and *Canavalia ensiformis (L.) DC.* Fabaceae have shown interesting broad spectrum antiviral. However, the clinical application of lectin proteins will require further in-depth research to circumvent inherent limitations including toxicity, stability and bioavailability in order to ensure that their druggable targets will offer a therapeutic benefit (Mitchell et al., 2017).

### Therapeutic Convergence in the Use of Antimalarial Plants Against Viral Infections in Africa

Antimalarial drugs derived from medicinal plants used in Traditional African Medicine have been found useful as repurposed drugs in managing other diseases including viral infections such as HIV, Ebola, and other viral hemorrhagic diseases due to lack of effective therapeutic agents. The active constituents of these plants have various mechanisms of action which are often not fully elucidated against malaria parasites. The complexity of these constituents sometimes lead to side effects that have been studied for repurposing them for the treatment of other conditions such as non-malaria infectious diseases (Das, 2015; Haładyj et al., 2018; Wolf et al., 2006). The geographical distribution between malaria and viral diseases where malaria endemic regions of the world such as Africa and Asia appear to experience relatively low cases of COVID-19-related mortalities led to the consideration of a possible therapeutic convergence between antimalarial plants (which have continued to be used against malaria in Africa) and viral pathogens including the dreaded SARS CoV-2. One possible explanation attributable to this unresolved therapeutic convergence is the mechanism of activity of these medicinal plants; several antimalarial phytomedicines which tend to produce more bioactivity as antioxidants, anti-inflammatory and immunomodulatory may function both as antimalarials and antiviral since the underlying mechanism of activity is not directly targeting the pathogen but rather boosting the immunity of the host, effective and efficient resolution of early inflammatory/anti-inflammatory cytokines (Afolayan et al., 2020) and scavenging of generated lethal free radicals (Iheagwam et al., 2020). This school of thought has been put forward to explain why many widely used African phytomedicines have gained more anecdotal claims of efficacy yet they do not easily kill the malaria parasite *in vitro* but produce good *in vivo* activity. For instance, Adebayo et al. (2017) demonstrated the poor *in vitro* but potent *in vivo* antimalarial activity of disulphide-rich peptide fraction of *Morinda lucida* (Adebayo et al., 2017)*.* These antimicrobial peptides have been reported to possess immunostimulating and antioxidant activities (Nguyen et al., 2016) as well as antiviral property (Boas et al., 2019). Apparently, the lethal COVID-19 is reported to be induced by the invasion of SARS CoV-2 into a human host and has been associated with cytokine storm (Jose and Manuel, 2020) and neutrophil-induced oxidative stress (Laforge et al., 2020) which often result in mortality. So, it is reasonable to assume that antimalarial plants widely used in TAM with well documented *in vivo* antioxidant, anti-inflammatory and immunomodulatory potentials might offer some therapeutic benefits in COVID-19 management. A treatize of antimalarial plants used in TAM with documented antioxidant, anti-inflammatory and immunomodulatory activities as well as level of documented evidence has been presented in Supplementary Table S1. However, the authorization of the repurposed use of these botanical antimalarials should be evidence-informed with impressive clinical data and supported by the best evidence. Considering repurposing antimalarial African traditional phytomedicines for COVID-19 management, endemic and naturalized African plants which have shown therapeutic promise as antimalarials following clinical studies should be considered and these include *Vernonia amygdalina*, *Nuclea pobeguinii* (Pobéguin ex Pellegr.) Petit, *Argemone mexicana* L.*, Artemisia annua* L.*, Citrus aurantiifolia* (Christm.) Swingle (Aracil and Green, 2019) and *Morinda lucida* Benth (Rubiaceae). Interestingly, available evidence indicates that these promising antimalarial plants additionally have the potential to tackle oxidative stress, regulate inflammatory response and stimulate the immune system to overcome complications observed in COVID-19 [**Level III**] (Haudecoeur et al., 2018; Asante et al., 2019; Madzuki et al., 2019; Afolayan et al., 2020; Jain et al., 2020; Zibaee et al., 2020). Meanwhile, some of these reports lack quality and will require validation. Bioactive compounds identified in the plants include; for *V. amygdalina* **-** vernolide **(116),** vernodalin **(117),** hydroxyvernolide **(120)** and vernodalol **(123),** vernoniosides B1-B3 and vernoniosides A1-A4 **(124)**; for *N. pobeguinii* - strictosamide **(138),** 19-O-methylangustoline, angustoline **(139)**, *A. Mexicana* - berberine **(140),** tetrahydroberberine, protopine **(141),** benzophenanthridines, 8-acetonyl dihydrosanguiranine, 8-methoxy dihydrosanguiranine **(142)*,*** *pancorine* **(144)*,*** *O-methylzanthoxyline* **(145)*,*** *nor-chelerythrine* **(125)*,*** *arnottianamide* **(146)** *cryptopine* **(147)*,*** *muramine* **(148)*,*** *argemexicaine A, argemexicaine B* **(149)** *;* for *A. annua* - artemisinin **(157)** *; C. aurantiifolia* - apigenin **(156)** and *Morinda lucida* - Morindin **(154),** oruwal **(152),** oruwalol **(155),** oruwacin **(150),** molucidin **(151),** Damnacanthal **(153),** Ursolic acid **(17),** polypeptides (Kraft et al., 2003; Challand and Willcox, 2009; Brahmachari et al., 2013; Haidara et al., 2016; Haudecoeur et al., 2018; Divneet Kaur, 2019). Overwhelming evidence supports the standardization of the leaf and seed of *M. oleifera* for a possible clinical application [**Level III**] as it has demonstrated broad range of antiviral activity in various studies (Biswas D. et al., 2020) while the disulphide-stabilized miniproteins (Morintides), lectins, hevein-like peptides, protein hydrolysates and glucosinolates/isothiocynates isolated from the plant have shown impressive effects, including as antiadhesives, anti-inflammatory, antioxidants and immunomodulatory compounds (Kini et al., 2017; Moura et al., 2017; Coriolano et al., 2018; Fahey et al., 2019; Liang et al., 2019; Sousa et al., 2020). Aside immunomodulation and free radical scavenging, one mechanism of activity of these lectins and stable polypeptides involve the competitive inhibition of adhesion of pathogen proteins to host polysaccharide receptors [**Level III, V**] (Sharon, 1986; Boas et al., 2019). Further *in vivo* and clinical evaluations will be required to assess the specific significance of these reports and in particular the possible role of Moringa-derived products in COVID-19 management.

Traditional African Medicines of the D. R. Congo and Nigeria have developed *N. pobeguinii* and *N. latifolia* for clinical application in malaria therapy which may form a starting point for herbal repurposing for COVID-19 management. For instance, a diherbal preparation containing *N. latifolia* and *Cassia occidentalis* (Manalaria^®^), was authorized for malaria treatment in D.R. Congo which later formed part of the Congolese List of Essential Drugs (Pousset et al., 2006; Memvanga et al., 2015; Haudecoeur et al., 2018). While in Nigeria, aqueous extracts of *N. pobeguinii* (codenamed PR 259 CT1) was successfully taken through preclinical investigation and phase 1 of clinical trials [**Level I**, for malaria] for the treatment of uncomplicated malaria (Mesia et al., 2011; Mesia et al., 2012a; Mesia et al., 2012b) and could offer hope in COVID-19 management after requisite investigative screening and standardization. Furthermore, the aqueous root extract of *N. latifolia* otherwise known as NIPRD AM1^®^, has been clinically studied in uncomplicated malaria and found to be therapeutically helpful as an antimalarial (Gamaniel, 2009) and should therefore be given attention for investigative management of COVID-19 [**Level I**, for malaria]. Nevertheless, such investigation should follow after these chemically complex herbal mixtures have been taken through extensive acute, subacute and chronic toxicity studies as well as the metabolite profiling using modern analytical methods.

*MAMA* Powder and *MAMA* Decoction are authorized indigenous polyherbal antimalarials which have been scientifically formulated by Prof Elujoba, the Head of the Village Chemist located within Obafemi Awolowo University, Ife, Nigeria. *MAMA* Powder contains stem bark of *Alstonia boonei* De Wild (Apocynaceae) and seed of *Picralima nitida* Stapf (Apocynaceae) while *MAMA* Decoction is made up of the leaves of *Mangifera indica* L. (Anacardiaceae), *Alstonia boonei* De Wild (Apocynaceae), *M. lucida* and *Azadirachta indica* A. Juss (Meliaceae) (Odediran et al., 2014). In an *in vivo* experiment using rodents (Adepiti et al., 2014), *MAMA* Decoction showed antimalarial activity at 240 mg/kg without any observable toxic effect when administered up to 2 g/kg body weight. Human observational study has further reinforced the *in vivo* activity while the efficacy claims by treated patients on *MAMA* herbal remedy has multiplied malarial patients’ demand for the herbal medicine. An elaborate preclinical study with superior scientific quality, documentation of chemical fingerprint as well as clinical trial and a possible repurposing for COVID-19 management is encouraged.

*Azadirachta indica* A. Juss. (Neem) (Dongoyaro, Margosa) Meliaceae, is a medicinal plant with more than 140 chemically active compounds isolated from the different parts including its flowers, leaves, seeds, roots, fruits, and bark and had been employed in managing many diseases. The active compounds have been identified as anti-inflammatory, anti-ulcer, anti-hyperglycaemic, immune-modulator, anti-mutagenic, anti-oxidant, antiviral and anti-carcinogenic drugs. The earliest three active constituents to be characterized namely nimbin **(81)**, nimbidin **(126)** and nimbinene **(127)** were described in 1942. Since then several compounds have been isolated and characterized and were shown to be chemically similar and biogenetically derivable from a tetracyclicterpenes. The neem kernel accumulates liminoids responsible for the bitterness and also found in other plant species such as Rutaceae and Simaroubaceae. Their biological activities include pesticides, antifeedants and cytotoxic properties. The leaves yielded quercetin **(73)** and nimbosterol as well as liminoids (nimbin and its derivatives). Quercetin **(73)** and Beta-sitosterol **(85)** were the first flavonoid and phytosterol purified from the fresh leaves of neem and were known to have antifungal and antibacterial activities (Fabricant and Farnsworth, 2001). Although the mechanism of action has not been fully elucidated, it is speculated that the observed therapeutic role of *Azadirachta indica* is due to the rich source of antioxidant and other valuable active compounds which include azadirachtin **(84)**, nimbolinin **(87)**, nimbin **(81)**, nimbidin **(126)**, nimidol **(89)**, salannin **(83)** and quercetin **(73**). An earlier study reported the virucidal activity of the leaf extract of *A. indica* against Coxsackievirus B-4 whose mechanism was proposed to be via interference with an early stage of the virus replication cycle (Badam et al., 1999). In a recent study, the *in vivo* intraperitoneal administration of methanol extracts of *A. indica* at a dose of 25 mg/kg body weight to murine hepatitis virus infected mice significantly reduced the expression of viral Nucleocapsid protein at the acute stage of infection. Since the murine hepatitis virus represents a prototype coronavirus, the therapeutic potential of the flavonoid, phytosterol and terpenoid-rich extracts of *A indica* has been reinforced [**Level V**]. *In vitro*, Neuro-2A cell-line treated with 200 μg/ml methanol extracts of *A. indica* inhibited virus-induced cell-to-cell fusion (Sarkar et al., 2020). More recently, a computational prediction of SARS-CoV-2 structural protein inhibitors from *A.* indica indicated their potential to inhibit the functionality of membrane and envelope proteins [Level IV*] (Borkotoky and Banerjee, 2020). The free radical scavenging activity has been linked to the presence of nimbolide **(88)** and azadirachtin **(84)** while the anti-inflammatory activity is thought to be via the regulation of proinflammatory enzyme activities including cyclooxygenase (COX) and lipoxygenase (LOX) enzyme (Biswas K. et al., 2020) [**Level IV**]. This plant, although a component of some polyherbal antimalarial remedies including *MAMA* Decoction, has not been extensively validated preclinically, clinically and standardized as an anti-infective remedy and therefore deserves further scientific attention especially as a potential herbal remedy in COVID-19 treatment.

Therefore, application of Neem in health management includes the use of its leaf, flower and stem bark in disease prevention because of its strong antioxidant potential (Sithisarn et al., 2005; Priyadarsini et al., 2009). The anti-inflammatory activity has been related to suppression of the functions of macrophages and neutrophils relevant to inflammation by nimbidin. Other findings revealed immunomodulator and anti-inflammatory effect of the stem bark and leave extracts, and antipyretic activities of the seed oil. The antimicrobial activities of Neem include inhibition of growth of organisms such as viruses, bacteria and pathogenic fungi (Ghonmode et al., 2013). The antimalarial activity of extracts using *Plasmodium berghei* revealed reduced level of parasitaemia with the limonoids being the active ingredients (Akin-Osanaiya et al., 2013). Another study using *P. falciparum* also showed significant reduction in both gametocytes and asexual forms of the parasite (Udeinya et al., 2008). Few of these studies lack depth and will require further work to make this plant an interesting candidate for clinical evaluation.

There are several compounds from various African plants that have been proven to have antimalarial properties which may provide researchers with starting points for antiviral drug discovery. Indoles with antimalarial properties have been derived from two plants species growing in Cameroon such as *Penianthus longifolius* Miers (Menispermaceae) and *Glossocalyx longicuspis* Benth (Siparunaceae). The compounds include Palmitine **(130)** from *P. longifolius* Miers, Linodenine from *G. brevipes* Benth. Also from Nigeria plant, there is Fagaronine **(128)** from *Fagara zanthoxyloides* Lam. (Rutaceae) and Alstonine **(129)** from *Picralima nitida* (Stapf) T. Durand and H. Durand (family Apocynaceae). *Triphyophyllum peltatum* (Hutch. and Dalziel) Airy Shaw (Dioncophyllacea) is a tropical African plant from which a potent antimalarial alkaloid, Habropetaline A **(131)** was isolated. The compound showed good effect against *P. falciparum*, without cytotoxicity, with respective IC_50_ values 5.0 and 2.3 ng ml^−1^ for the strains K1 (Chloroquine and pyrimethamine resistant) and NF54 (sensitive to all known drugs). It was found to be almost as active as artemisinin and one of the most potent natural products used against *P. falciparum* (Bringmann et al., 2003). There are several observations that point to the fact that naphthoisoquinoline alkaloids are promising lead compounds for the development of anti-malarial drugs which of course could be tried against viral pathogens. Cryptolepines **(36)** from *Sida acuta* Burm.f. (Malvaceae), a plant growing in Ivory coast showed a good antimalarial activity (Banzouzi et al., 2004). *Cryptolepis sanguinolenta* (Lindl.) Schltr. of the family Periplocaceae growing in diverse regions in Africa, have also exhibited potent anti-malarial properties (Ablordeppey et al., 1990; Cimanga et al., 1999; Barku et al., 2012). Following a recently reported *in silico* experiments, several of these antimalarial alkaloids from African plants have shown interesting predicted inhibition of SARS CoV-2 viral proteins [**Level IV**] (Li et al., 2005; Borquaye et al., 2020) and this support the need for further *in vitro*, *in vivo* and clinical investigation on their therapeutic potential for COVID-19 treatment.

Bisnorterpenes, purified from the roots of *Salacia madagascariensis* Lam. DC. of the family Celastraceae, a shrub found in East Africa whose roots are used in the treatment of malaria fever and menorrhagia specifically in Tanzania for its potent antiprotozoal activity (Murata et al., 2008). Recent *in silico* studies supports the anti-SARS CoV-2 activity [**Level IV**] of bisnorterpenes such as 22-Hydroxyhopan-3-one and 6-Oxoisoiguesterin which have been isolated from endemic African plants with impressive binding affinities for the 3CL^pro^ of coronaviruses of −8.6 and 9.1 kcal mol^−1^ respectively (Gyebi et al., 2020). *Aframomum exscapum* (Sims) Hepper (Zingiberaceae) synthesizes acyclic triterpenes compounds such as S-nerolidol **(157)** isolated from the seeds and represents an important constituent of essential oils used in the treatment of malaria. This compound is also found in *Artemisia herba alba* Asso and in *Cymbopogon citratus* (DC.) Stapf. (Poaceae), and is able to arrest development of the intraerythrocytic stages of malaria (Titanji et al., 2008) and as such may be considered in future search for anti-viral agents including SARS-CoV-2. *Hyptis suaveolens* (L.) Poit. from Nigeria has also yielded abietane-type diterpenoid endoperoxide, a molecule with high anti-plasmodial activity (Chukwujekwu et al., 2005). Sesquiterpenes and sesquiterpene lactones **(51)** derived from *Vernonia* spp. are known to have interesting anti-plasmodial activities. Vernodalin **(132)** is the most active compound in bitter leaf. The plant has many uses in Traditional African Medicine, the leaves of *V. amygdalina* Del. are used in the treatment of various diseases including malaria and viral infections. Recent *in silico* anti-SARS-CoV-2 investigation reported promising activity of terpenes, iridoids and lignans which are able to effectively interact with the host enzyme transmembrane protease serine 2 (TMPRSS2) [**Level IV**]. This enzyme facilitates viral particle entry into host cells, and its inhibition blocks virus fusion with angiotensin-converting enzyme 2 (ACE2). The structural complexity of these plant metabolites and the presence of hydroxyl moieties and aromatic rings significantly improves the inhibition of their molecular target (Rahman et al., 2020). Traditional African Medicine knowledge could be very useful in drug discovery efforts from African medicinal plants, but the quality and reproducibility of such investigation is key. Chinedu and colleagues in a review of plants used in malarial treatment, reported over one hundred indigenous plants which have been employed traditionally in the management of malaria infection in six African Countries namely Nigeria, Ghana, Ethopia, Benin, Cameroon and Togo (Chinedu et al., 2014). Komlaga and colleagues have also evaluated some of the plants employed in the traditional management of malaria in Ghana, namely *Persea americana* Mill (Lauraceae), *Theobroma cacao* L. (Malvaceae) and *Tridax procumbens* (L.) L. (Compositae) and found that they have good antiplasmodial activities to justify their employment in such treatment (Komlaga et al., 2015). African Medicinal plants used in treating malaria may therefore represent promising areas to investigate for their potential in treating viral infections including the novel coronavirus (COVID-19) and HIV. However, since their findings are only preliminary, there is still a long path to clinical application as these remedies must be well standardized, authorized for use and administered by qualified medical personnel to African populations.

### Beyond Claims: Identifying Key COVID-19 Potential Phytotherapies in Africa

**FIGURE 6:**
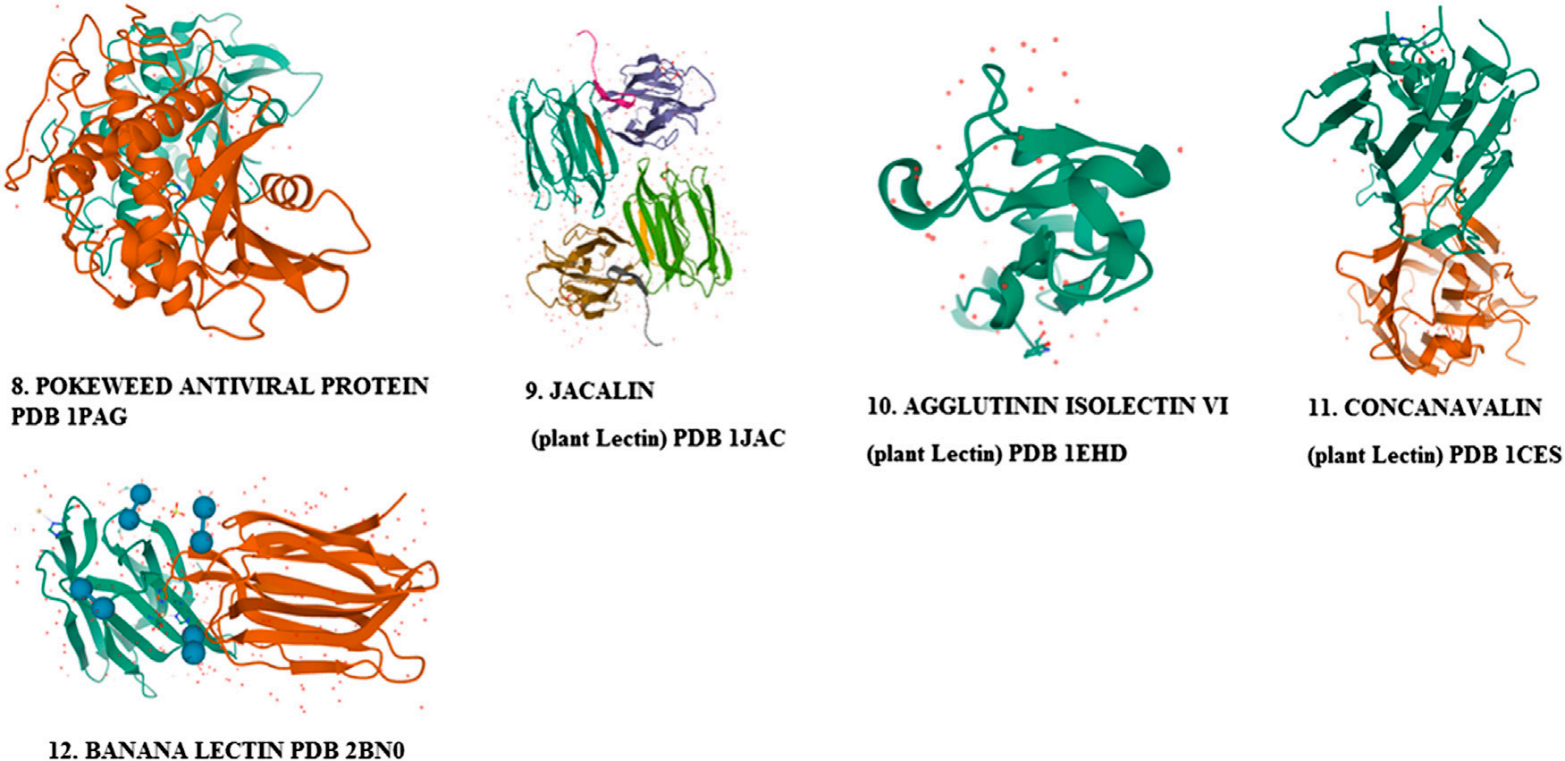
Antiviral proteins isolated from plants.

**FIGURE 7:**
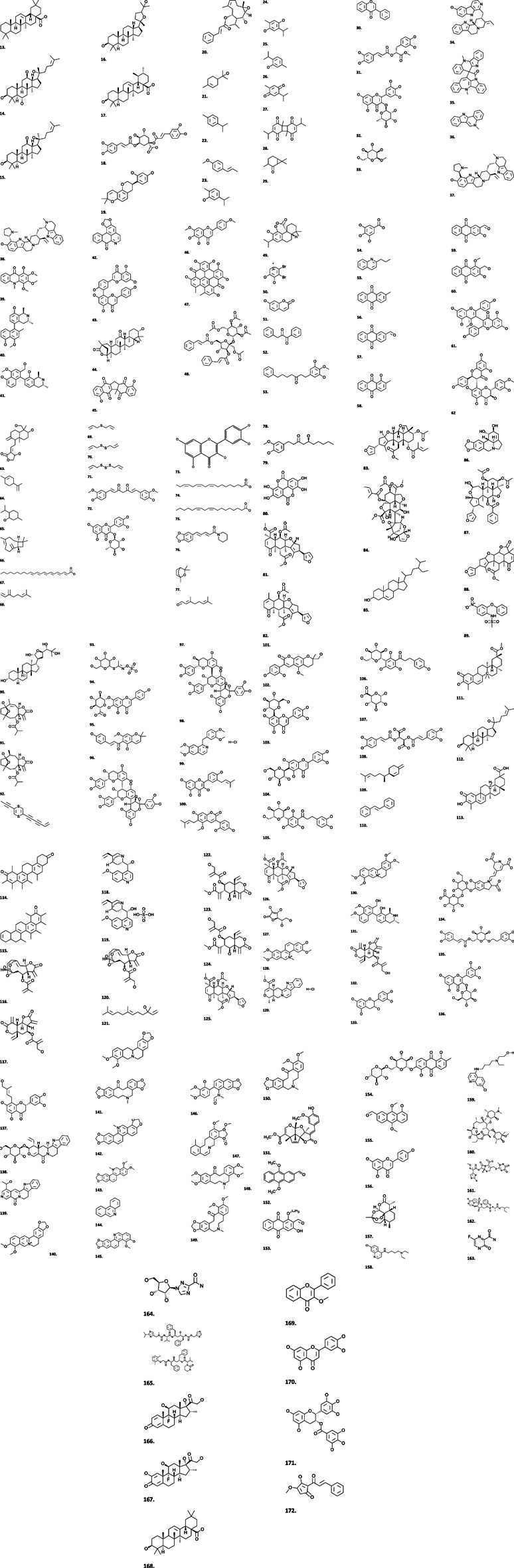
Structures of some plant-derived secondary metabolites with antiviral activities.

Medicinal plants have continued to play an important role in providing primary healthcare needs across the African region particularly during sudden outbreak of deadly diseases like COVID-19. Emerging technologies, including the mining of plant-derived chemical libraries and application of computational techniques including ligand docking and other methods in computer-aided drug design (CADD), are increasingly deployed in rapidly selecting candidate screening compounds for a fast-tracked drug discovery process particularly during emergency situations like the ongoing COVID-19 pandemic. *In silico* analysis reduces the investigational time-line to identify “hits” and the analysis of their suitability in combating pathogenic diseases and thus shortens the drug discovery pipeline (Terstappen and Reggiani, 2001; Pascolutti and Quinn, 2014; Ubani et al., 2020). Documented hits compounds which have demonstrated interesting *in silico* activities against SARS-CoV-2 and isolated from African plants (Figure 7) include amaranthin **(134)** (*Amaranthus tricolor* L.- Amaranthaceae), myricitrin **(32)** (*Myrica cerifera* (L.) Small - Myricaceae), isoflavones **(30)** (*Psorothamnus arborescens* (A.Gray) Barneby - Fabaceae), nigellicine **(21)**, nigellidine **(22)**, nigellone **(28)**, carvacrol **(24)**, hederin **(25)**, thymol **(26)**, thymoquinone **(27)**, thymohyroquinone **(29**) (*Nigella sativa* L.), Calceolarioside B **(135)** (*Fraxinus sieboldiana* Blume - Oleaceae), Licoleafol **(137)** (*Glycyrrhiza uralensis* Fisch. ex DC - Fabaceae), methyl rosmarinate **(31)** (*H. atrorubens* Poit), myricetin 3-O-beta-D glucopyranoside **(136)** (*Camellia sinensis* L. Kuntze - Theaceae). Table 2 presents a full list of these compounds and the plants producing them while Figure 6 presents the chemical structures of the compounds. These *in silico* findings with limited evidence should form the basis for future in-depth *in vitro*, *in vivo* and clinical studies rather than indiscriminate application of preliminary data which could constitute a public health concern.

**TABLE 2:** African Plants with evidence-based *in silico* therapeutic potentials against SARS-CoV-2 [Level IV].

African plant	Country	Plant organ	Bioactive compound tested *In silico*	Viral protein targeted	Binding affinity (Kcal/mol)	References
*Amaranthus tricolor* L. (Amaranthaceae)	Nigeria (Benin), Kenya and Tanzania	Seedlings	Amaranthin **(134)**	^b^SARS-CoV-2 3CLpro	−18.14	Kaur et al. (2006), National Center for Biotechnology Information (2020); ul Qamar et al. (2020), Wu et al. (2006), Xin et al. (2011)
*Camellia sinensis* L. Kuntze (Theaceae)	East Africa (Kenya, etc)	Root barks	Myricetin 3-O-beta-d-glucopyranoside **(136)**	^b^SARS-CoV-2 3CLpro	−18.42	Xu et al. (2008)
*Fraxinus Sieboldiana* blume (Oleaceae)	Sub-saharan Africa	Stem bark	Calceolarioside B **(135)**	^b^SARS-CoV-2 3CLpro	−19.87	Kim et al. (2002), Lin et al. (2008), Lin et al. (2007), National Center for Biotechnology Information (2020)
*Glycyrrhiza uralensis *Fisch. ex DC*.* (Fabaceae)	South Africa, Libya	Leaves	Licoleafol **(137)**	^b^SARS-CoV-2 3CLpro	−19.64	Das et al. (2020), National Center for Biotechnology Information (2020), ul Qamar et al. (2020)
*Hyptis atrorubens* Poit (Lamiaceae)	Nigeria, sub-saharan Africa	Leaves and stem	Methyl rosmarinate **(31)**	^b^SARS-CoV-2 3CLpro	−20.62	Abedini et al. (2013), National Center for Biotechnology Information (2020), ul Qamar et al. (2020); Woo and Piao (2004)
Myrica *Cerifera* L. (Myricaceae)	Nigeria	Root bark	Myricitrin **(32)**	^b^SARS-CoV-2 3CLpro	−22.13	National Center for Biotechnology Information (2020), Paul et al. (1974), ul Qamar et al. (2020)
*Nigella sativa* L*.* (Ranunculaceae)	Algeria	Seed oil	A-terpineol **(21)**	^a^SARS-CoV-2:ACE2 interface	−5.8	Ahmad et al. (2020), Ali and Blunden (2003)
*Nigella sativa* L. (Ranunculaceae)	Algeria	Seed oil	P-cymene **(22)**	^a^SARS-CoV-2:ACE2 interface	−5.8	Ali and Blunden (2003), Malik et al. (1995)
*Nigella sativa* L. (Ranunculaceae)	Algeria	Seed oil	T-anethole **(23)**	^a^SARS-CoV-2:ACE2 interface	−6.2	Ali, B. H. and Blunden (2003), Malik and Zaman (1992)
*Nigella sativa* L. (Ranunculaceae)	Algeria	Seed oil	Carvacrol **(24)**	^a^SARS-CoV-2: ACE2 interface	−7.0	(Arunasree (2010), Azizi et al. (2012), Lima et al. (2013), Landa et al. (2009), Li et al. (2016), Yin et al. (2012)
*Nigella sativa* L. (Ranunculaceae)	Algeria	Seed	Thyhydromoquinone **(25)**	^a^SARS-CoV-2:ACE2 interface	−6.1	Khan et al. (2011), Salim. (2020), Worthen et al. (1998)
*Nigella sativa* L. (Ranunculaceae)	Algeria	Seed oil	Thymol **(26)**	^a^SARS-CoV-2:ACE2 interface	−6.1	Bulugaha and Arachchige. (2012), Islam et al. (2004), Salem (2005)
*Nigella sativa* L*.* (Ranunculaceae)	Algeria	Seed oil	Thymoquinone (TQ) **(27)**	^a^SARS-CoV-2:ACE2 interface	−6.7	Badary et al. (2003), Bulugahapitiya and Arachchige (2012), Houghton et al. (1995), Kacem and Meraihi (2006), Randhawa, (2008), Salem (2005), Salim (2020)
*Nigella sativa* L*.* (Ranunculaceae)	Algeria	Seed	Dithymoquinone (nigellone) **(28)**	^a^SARS-CoV-2:ACE2 interface	−8.6	El-Dakhakhny (1965), Mahmoud et al. (2002), Randhawa (2008), Salem (2005)
*Nigella sativa* L. (Ranunculaceae)	Algeria	Seed	Carone	^a^SARS-CoV-2:ACE2 interface	−6.5	Salem (2005), Salim. (2020)
*Phaseolus vulgaris* L. (Fabaceae)	Nigeria, sub-saharan Africa	Root	3,5,7,3′,4′,5′-hexahydroxy flavanone-3-Obeta-d-glucopyranoside **(33)**	^b^SARS-CoV-2 3CLpro	−19.10	National Center for Biotechnology Information (2020), Rao, (1990), ul Qamar et al. (2020)
*Phyllanthus Emblica* L. (Phyllanthaceae)	Nigeria, Ghana, North Africa	Leaves and branches	(2S)- eriodictyol 7-O-(6″ O'galloyl)-beta-dglucopyranoside **(33)**	^b^SARS-CoV-2 3CLpro	−19.47	National Center for Biotechnology Information (2020), ul Qamar et al. (2020)
*Psorothamnus fremontii *(Torr. ex A.Gray) barneby (Fabaceae)	Uganda, South Africa	Roots	5,7,3′,4′-tetrahydroxy-2'-(3,3-dimethylallyl) isoflavone **(30)**	^b^SARS-CoV-2 3CLpro	−29.57	National Center for Biotechnology Information (2020), ul Qamar et al. (2020)

^a^ SARS-CoV-2:ACE2 interface: Binding affinities of docked compounds were obtained using Autodock/vina with Chloroquine as reference standard scoring a binding energy of -7.2; Dithymoquinone (nigellone) (28) demonstrated the most promising binding energy lower than the reference standard (Ahmad et al., 2020).

^b^ SARS-CoV-2 3CLpro, Molecular Operating Environment (MOE) was used for molecular docking, ligand-protein interaction and drug likeness analyses while the antiviral drug, nelfinavir was used as the standard drug which produced a binding energy of −17.31. All compounds reported showed a lower binding energy than the reference compound used.

Attempts are at present being fast-tracked to discover, repurpose or otherwise develop preventive and treatment options for COVID-19 from the wealth of indigenous knowledge on the use of plants sourced from African plant biodiversity in combating infectious diseases. However, for a phytomedicine to be officially approved and authorized for use, it needs to be scientifically investigated and taken through accelerated clinical trials. The African media, especially the social media, internet, television and radio has been populated with anecdotal claims on COVID-19 herbal vaccines, symptomatic treatment and even cure. Several of these claims are coming from important personalities in the society including religious leaders, traditional/community leaders, Traditional Medical Practitioners (TMPs), research institutions or from establishments producing herbal remedies. Many of these yet-to-be validated claims have originated from Eastern Africa (Madagascar), West Africa (Nigeria) and Central Africa (Cameroon). In fact, Madagascar was the foremost African country to authorize the use of an indigenous herbal remedy known as COVID Organics (CVO) for the prevention and cure of COVID-19. The World Health Organization (WHO) carefully discouraged the official positioning of CVO as a magic bullet for the cure of the disease and emphasized that only evidence-based claims with satisfactory efficacy and safety margins via clinical trials could justify the claims of the government of Madagascar. As a result, the WHO and African CDC are cooperating with and supporting the government to design and conduct clinical trials to validate the efficacy and possible adverse effects of CVO polyherbal formulation. This may involve multi-centre clinical trials involving countries in Africa such as Tanzania, Equatorial Guinea and Congo-Brazzaville that had received the herbal remedy (WHO, 2020).

In Nigeria, the social media, television, internet and radio media have been flooded with claims of symptomatic treatment, cure or prevention of COVID-19. Many of these anecdotal claims flying over the virtual space have provided African researchers starting points for a plant-derived drug discovery studies against COVID-19; many of the claims have originated from eminent Nigerians such as the traditional leader of the Yoruba nation, religious prophets and Priests, Botanists, Biochemists and a host of Nigerian scientists in academia; several of these claims are currently under scientific investigation for adverse effects and efficacy. Officially, the Nigerian government has not approved or authorized the use of any indigenous phytomedicine to combat COVID-19, reason being that no herbal remedy currently claimed to prevent, manage or cure the infectious disease has been taken through a rigorous scientific investigation via clinical trials. Meanwhile, the Nigerian government through the National Agency for Food and Drug Administration and Control (NAFDAC) is now processing not less than 21 herbal formulations for “safe use” under listing status. These polyherbal formulations according to NAFDAC, have been claimed to boost immunity with a parallel anti-infective activity capable of providing relief to symptoms associated with COVID-19. More so, a documented evidence of clinical trial which is required to support efficacy claims is lacking until the time of this writing. However, the Bioresources Development Group (BDG), Abuja, Nigeria; International Center for Ethnomedicine and Drug Development (InterCEDD) Nsukka, Nigeria, has submitted the previously NAFDAC listed IHP Detox tea for clinical trials which is titled: “Efficacy and safety of IHP Detox Tea (a special blend of *Andrographis paniculata* (Burm.f.) Nees (Acanthaceae), *Garcinia kola* Heckel (Clusiaceae) and *Psidium guajava* L. (Myrtaceae)) for treatment of COVID-19): a pilot placebo-controlled randomized trial”. The clinical trial is to be undertaken at the Nigeria Center for disease Control (NCDC) COVID-19 isolation site in Lagos, Nigeria and has been registered with the Pan African Clinical Trials Registry: at www.pactr.org with registration number of PACTR202004761408382. The identified main bioactive phytoconstituents of the *Andrographis paniculata* is andrographolide **(61)** while kolaviron, Garcinia biflavonoids (**59–60)** has been reported in *Garcinia kola* (Lin et al., 2009; Buba et al., 2016).

Other indigenous anti-COVID-19 herbal remedies and polyherbal formulations listed by the Nigerian NAFDAC but still lack clinical trial data and not yet authorized for use by the government but available in the market space include: IHP Garcinia, IHP Detox, IHP Immunovit (products of (InterCEDD, Nigeria), CUGZIN capsule, 290 mg (produced by PaxHerbal, Nigeria) and VIVE active (Rx Agroprocessing, Nigeria). The Nigerian Federal Ministry of Health in collaboration with NAFDAC is supporting three foremost and promising remedies for funding considerations to enable clinical trials in a bid to champion an evidence-informed use of indigenous phytomedicines in Nigeria.

Cameroon is another country located in central Africa whose anti-COVID-19 herbal claims has attracted much attention and use of unauthorized herbal remedies is widespread despite serious concerns expressed by the WHO regarding such uninvestigated anecdotal claims which could place African populations in great risk, create false confidence and discourage them from adherence to recommended global preventive measures. For instance, two phytomedical remedies (Elixir COVID and Adsak COVID) which have been developed from undisclosed indigenous plants have been claimed to reverse the effect of COVID-19, clear the virus from patients’ body fluid while essential oils have been claimed to cure at least 1500 COVID-19 patients. These remedies lack scientific evidence (Africa CDC, 2020; WHO, 2020) and should be holistically validated for a possible clinical application.

### Perspectives on the Therapeutic Potentials of African Plants

Africans may lack access to western repurposed drugs that are now used to manage COVID-19 in developed countries, but they have unlimited access to medicinal plants which can be standardized for effective and safe use. These tropical plants accumulate both primary and secondary metabolites with a broad range of *in silico*, *in vitro* and *in vivo* activities including antiviral properties **(** Tables 1–3). Many of the antiviral primary metabolites such as polysaccharides and antiviral proteins (Figures 5, 6) accumulated in African plants reported in this review have not attracted much research attention and exploitation in antiviral drug discovery. Even of more scientific interest are the highly stable low molecular weight peptides known as cysteine-knot peptides among which, cyclotides (Figure 5; 1–7) are most stable due to their continuous circular configuration, low molecular weight, abundance, sequence variability, oral bioavailability, target specificity, low *in vivo* toxicity and wide distribution in plants families including Violaceae, Rubiaceae, Fabaceae, Curcubitaceae and Solanaceae (Gründemann et al., 2013; Attah et al., 2016b; de Veer et al., 2019). Reported antiviral cyclotides include Cter M **(1)**, vhl-2 **(2)**, cyclotide vhl-1 **(3)**, CIRCULIN A **(4)**, kalata B1**(5)**, kalata B8 **(6)**, Cyclotide Palicourein **(7)** and Alstotide S1 **(8) (** Daly et al., 1999; Daly et al., 2004; Chen et al., 2005; Poth et al., 2011; Wang et al., 2017 **)**. The hydrophobic nature of these interesting peptides appear to be very important for their activity against enveloped viruses (Badani et al., 2014; Wang et al., 2017). Antiviral Kalata B1 and B8 have been isolated from an indigenous plant *Oldenlandia affinis* (Roem. and Schult.) DC. (Rubiaceae) used in Traditional African Medicine to aid delivery in Central Africa (Gran et al., 2000) and as an antimalarial herb in Nigeria (Nworu et al., 2017); cyclotide-rich aqueous extract of *Oldenlandia affinis* DC. represent a potential multitarget peptide drug candidate that awaits scientific investigation against COVID-19. However, phytomedicines containing antiviral Kalata B1 may be contraindicated in pregnancy (Saether et al., 1995) and more useful during the late stage of hyper-inflammation observed in COVID-19 owing to the immunosuppressant activity of Kalata B1 (Gründemann et al., 2013). Meanwhile, the therapeutic potentials of these peptides still lacks clinical evidence to support the interesting *in vitro* and *in vivo* findings.

**TABLE 3:** African Plants which are less widely applied in TAM with *in vivo* and *in vitro* evidence-based antiviral potentials [Level V].

African plant	Country	Plant organ	Bioactive compound isolated	Viral protein targeted	References
*Alangium chinense* (Lour.) harms (Cornaceae)	Cameroon, Ethiopia, tropical Africa.	Roots	Sesquiterpenoids and alkaloids	Coxsackie B3	Zhang et al. (2013)
*Azadirachta indica* A. Juss (Meliaceae)	Ghana	Bark	Bark extract	HSV-1	Martins et al. (2009)
*Azadirachta indica* A. Juss (Meliaceae)	Ghana	NP	Polysaccharides	Poliovirus	Faccin-Galhardi et al. (2012)
*Calophyllum* L. (Calophyllaceae)	Kenya, Madagascar	NP	Coumarin and xanthone	HIV RT^≠^	
*Camellia sinensis* (L.) kuntze (Theaceae)	South Africa	Green tea	Epigallocatechin **(171),** lucidone **(172)**	HBV	Xu et al. (2008)
	Kenya				
	Malawi				
	Rwanda				
	Nigeria				
*Cryptopleura ramosa* (hudson) L. Newton (Delesseriaceae)	South Africa	NP	Sulfated galactans	HSV-1 and HSV-2 replication in vero	Carlucci et al. (1997)
*Ferula narthex *Boiss. (Apiaceae)	North Africa	NP	Sesquiterpenecoumarins **(51)**	Influenza	Lee et al. (2009)
Glycine max (L.) Merr. (Fabaceae)	Zambia, Zimbabwe and South Africa	NP	Rhamnogalacturonan	CMV^≠^ cytotoxicity	Steinmassl and Anderer (1996), Huisman et al. (2001)
*Glycyrrhiza glabra* L. (Fabaceae)	North Africa	Leaflets	Chalones **(52)**	Influenza	Dao et al. (2011)
*Griffithsia* (wrangeliaceae)	South Africa	NP	Griffithsin	HIV clade C	Danaher et al. (2011)
*Hypericum perforatum* L. (hypericaceae)	South Africa	Stem and petals	Hypercin **(47)**	HCV^≠^	Jacobson et al. (2001)
*Ligustrum lucidum* W.T.Aiton (Oleaceae)	South Africa		Oleanolic acid **(168)** and ursolic acid **(17)**	HCV	Kong et al. (2013)
	Algeria				
*Marrubium peregrinum* L. (Lamiaceae)	Northern Africa	NP	Ladanein **(173)** (BJ486K), a flavonoid	All HCV genotypes	Haid et al. (2012)
*Momordica charantia* L. (Cucurbitaceae)	Nigeria	NP	Recombinant MAP 30	HIV	
*Phyllanthus niruri* L. (Phyllanthaceae)	West Africa	Leaf	^b^Niruriside **(48)**	HIV	Dharmaratne et al. (2002), Lee-Huang et al. (1995), Qian-Cutrone et al. (1996)
*Piper longum* L. (Piperaceae)	Madagascar		Longumosides and amide alkaloids	HBV	Jiang et al. (2013)
*Punica granatum *L. (*Lythraceae*)	North Africa		Punicagalin	Enterovirus 71	Mouhajir et al. (2001), Yang et al. (2012)
*Punica granatum* L. (Lythraceae)	South Africa	NP	^a^Polyphenols	Enveloped viruses, Food borne surrogate viruses	Kotwal (2008), Neurath et al. (2004), Neurath et al. (2005), Su et al. (2010), Sundararajan et al. (2010)
*Reynoutria japonica* houtt. (Polygonaceae)	South Africa	Leaves	^c^Resveratrol+	HIV, EBV, HCV	De Leo et al. (2012), Heredia et al. (2000)
*Rubus fruticosus* L. (Rosaceae)	South Africa	NP	Extract	HSV-1^≠^	Danaher et al. (2011)
*Salvia rosmarinus* spenn. (Lamiaceae)	North Africa	Np	Carnosic **(49)**	RSV	Shin et al. (2013)
	Ethiopia				
*Sambucus nigra* L. (Adoxaceae)	Northern Africa	NP	Liquid extract	Influenza	Krawitz et al. (2011)
*Swietenia macrophylla* king (Meliaceae)	West Africa	Stem	^d^3-hydroxy caruilignan (3-HCL-C)	HCV	Wu et al. (2012)
*Woodfordia fruticosa *(L.) kurz (Lythraceae)	Tanzania, Madagascar	Flowers	Gallic acid **(54)**	Enterovirus HCV	Choi et al. (2010)

^NP^Not Provided. ^≠^Only *in vitro* activitiy reported;

^a^ HIV-1 entry inhibitors from pomegranate juice adsorbed onto corn starch. The resulting complex blocks virus binding to CD4 and CXCR4/CCR5 and inhibits infection by primary virus clades A to G and group O; the antiviral effects of pomegranate polyphenols are mediated in different ways depending on the nature of the virus. In the case of influenza virus, elimination of infectivity by pomegranate polyphenols is primarily a consequence of damage to virion integrity, rather than simply a coating of viral particles.

^b^ inhibitory activity against protein binding to RNA.

^c^ protein synthesis inhibition, decreases reactive oxygen species (ROS) levels, and suppressession of the EBV-induced activation of the redox-sensitive transcription factors NF–kB and AP-1.

^d^ 3-HCL-C interfered with HCV replication by inducing IFN-stimulated response element transcription and IFN-dependent anti-viral gene expression. HIV–Human Immunodeficiency Virus; HSV 1–Human Simplex Virus one; HSV 2–Human Simplex Virus two; EBV–Epstein-Barr Virus; CMV–Cytomegalovirus; HBV–Hepatitis B Virus; RSV–Respiratory Syncytial Virus; HCV–Hepatitis C Virus.

Exploring and exploiting medicinal plants for antiviral activity should be premised on the demonstration of prophylactic and/or therapeutic efficacy at an optimal amount in metabolic fluid. Similarly, plants and their bioactive metabolites have been shown to modulate immunological activities making them suitable candidates for biological response modifiers with the potential to alleviate symptoms and prevent death associated with infectious viral outbreak (Kurokawa et al., 2010). Therefore, the Africa Centers for disease Control and Prevention (Africa CDC) has provided standard guidelines for Member States when herbal remedies or medicines are proclaimed or developed in their countries (Africa CDC, 2020)*.*

Since the global R and D community is relentlessly working on getting an effective treatment to stop the COVID-19 pandemic, symptomatic management of the viral symptoms and Prevention of infection through divergent approaches should be encouraged. For instance, evidence-based and documented scientific publications on the antiviral and immunomodulatory potentials of African plants could provide some clues on prevention and management of COVID-19. Examples of such African plants widely used in traditional medicine across the region which have received *in silico* anti-COVID19 screening (Rowaiye et al., 2020) for bioactivity include *M. indica* L.*, Manihot esculenta* Crantz. (Euphorbiaceae)*, A. occidentale* L.*, Uraria picta* (Jacq.) Desv. (Fabaceae) *and Corchorus olitorius* L.(Malvaceae) Others are simply immune boosters including *V. amygdalina* Delile.*, M. oleifera* Lam*, Telfairia occidentalis* Hook.f. (Cucurbitaceae), among others. Findings from this preliminary study have limited evidence until indepth preclinical and clinical studies are done. Some commonly used Nigerian medicinal plants that may have potentials for the symptomatic management of COVID-19 include *Capsicum L* (Solanaceae), *Z. officinale* Roscoe, *Xylopia ethiopica* (Dunal) A.Rich. (Annonaceae)*, C. papaya* L.*, A. cepa* L.*, G. kola* Heckel*, A. sativum* L*.* Several other antiviral plants (Table 1) used in Nigerian ethnomedicine such as *Senna siamea* (Lam.) H.S.Irwin and Barneby (Fabaceae) and *Zephyranthes candida* (Lindl.) Herb (Amaryllidaceae) (Ogbole et al., 2013) could also be of scientific interest for further research. Tannins and glucosinolates **(94)** with broad anti-infective activities (Chodur et al., 2018; Hensel et al., 2020; Nie et al., 2020) from seeds of *M. oleifera* Lam.*,* a popular and widely used tropical plant may equally be of research interest as potential prophylactic and anti-COVID-19 herbal supplement. *B. ferruginea* Benth is another tropical plant for future investigation against COVID-19; it is popular in African ethnomedicine to fight difficult infectious diseases as well as a prophylactic in some anti-infective remedies [**Level V**] (Ozerov et al., 1994; Cimanga et al., 1999). *P. guajava* L. has shown interesting broad spectrum antimicrobial activities, good antiviral property and polyphenolic compounds (catechin-**133**, quercetin-**73** and gallic acid - **54**) derived from the stem bark and leaves have been linked with the reported bioactivity (Sriwilaijaroen et al., 2012; Naseer et al., 2018; Trujillo-Correa et al., 2019). Howbeit, this study lacks *in vivo* and clinical evidence as only preliminary in silico and a more elaborate *in vitro* data has been documented.

A recent CADD-directed fluorogenic enzyme inhibition assay reported corilagin and rhoifolin, two natural products of African origin, with micromolar range inhibitory activity against the main protease (3CLpro) enzyme of the SARS-CoV2 (Loschwitz et al., 2020). Interestingly, the investigation which had commenced with the screening of an over 1.2 million virtual compound library (Olubiyi et al., 2020) identified corilagin and rhoifolin in the top eight compounds with respect to main protease inhibitory activity. Corilagin, an ellagitannin, is widely distributed in several African plants and is known to be present in the plant families including Euphorbiaceae (e.g. *Acalypha wilkesiania* Muell Arg and *Acalypha australis* L., *Euphorbia longana* Lam., *Phyllanthus emblica* L., *P. urinaria* L., *P. tenellus* Roxb., *P. niruri* L., etc), Geraniaceae (*Geranium sibiricum* L.), Combretaceae (*Terminalia catappa* L.), to mention a few. Rhoifolin on the other hand is a tri-substituted flavone and has been reported in *Uraria picta* (Jacq.) DC, a perennial tropical plant with distribution extending through most parts of Sub-Saharan Africa. With *in vitro* inhibitory activities of both natural products in the micromolar range, it is yet to be determined if the reported potencies will extend to *in vivo* situations. But the establishment of the SARS-CoV2 3CLpro inhibitory activities for both natural products further support the potential of African plants to potentially furnish herb-based remedies and lead compounds that can be developed into clinically useful treatment for COVID-19 [**Level III**].

### Coadministration of Phytomedicines and Western Medicines in COVID-19 Management: Drawback of Herb-Drug Interaction

The use of phytomedicines as adjuvants in the therapeutic treatment of diseases has received a drawback due to the occurrence of deleterious herb-drug interactions (Rivera and Loya, 2013). A typical medicinal plant is a biological laboratory of hundreds of bioactive metabolites with significant influence on the pharmacokinetics and pharmacodynamics of drugs when co-administered or used as adjuvants. The co-administration of orthodox drugs alongside herbal medicines may bring about pharmacodynamic interactions that may result in synergistic, additive or antagonistic pharmacological end-points. More so, an important consequence of herb-drug interactions is the pharmacokinetic dimension that alters the level of the drug in systemic circulation. This may result from the activity of the herbs leading to elevation or inhibition of the function of certain drug metabolizing enzymes or efflux transporters. As a rule of the thumb, the bioavailabilities of bioactive compounds are enhanced by the inhibition of drug metabolizing enzymes or efflux transporters; in contrast, induction of drug metabolizing enzymes or efflux transporters reduces drug bioavailability (Patil et al., 2014). Hence, precautionary measures and adequate monitoring (pharmacovigilance) are essential when co-administering drugs with narrow therapeutic window or safety margin with herbs as any variation in plasma concentrations can result in adverse events or treatment failure (McFadden and Peterson, 2011).

Generally, most of the drugs used in humans are metabolized by a class of enzymes known as cytochrome P450 (CYP) (Thomford et al., 2016). The CYP enzyme comprises diverse isoenzymes whose function can be altered by phytochemicals present in phytomedicines. In herb-drug interaction, the herbal drug may induce or inhibit the same isoenzyme that is responsible for the metabolism of the synthetic drug. If the co-administered herbal drug inhibits the isoenzyme, the synthetic drug will not be metabolized; this will lead to high levels of the drug in physiological fluid which consequently results in toxicity. In contrast, if the herbal drug induces the isoenzyme, this could result in rapid metabolism of the drug whose optimal therapeutic concentration in systemic circulation may not be reached leading to treatment failure, and possibly development of resistance (Zhou et al., 2003). Herb-drug interaction can also occur if the same isoenzyme is responsible for the metabolism of both the herbal drug and synthetic drug. For instance, *in vitro* studies have shown that CYP3A4, 3A5 and CYP19 enzymes are inhibited by hypoxoside, an active component of *Hypoxis hemerocallidea* Fisch., C.A. Mey. and Avé-Lall. (Hypoxidaceae); *Hyptis suaveolens* (L.). Poit. (Lamiaceae), *Boerhavia diffusa* L (Nyctaginacea), *Launaea taraxacifolia* (Willd.) Amin ex C. Jeffrey (Asteraceae) and *Myrothamnus flabellifolia* Welw. (Myrothamnaceae) inhibit CYP2B6 activity in a concentration -dependent manner. *Sutherlandia frutescens* (L.) R.Br. (Fabaceae) was shown to inhibit CYP3A4. In other words, the drugs metabolized by these enzymes will become toxic if administered concurrently with these plants commonly used in African Traditional medicine. On cultured cells, *Agarista salicifolia* (Lam.) G.Don (Ericacea), *Turraea holstii* Gürke (Meliaceae) and *Sterculia africana* (Lour.) Fiori (Malvacea) causes more than two-fold induction of CYP3A4 mRNA (Mills et al., 2005). Unfortunately, only limited data exist in Africa regarding *in vivo* herb-drug interaction since most patients do not report intake of herbal medicines to health practitioners during treatment. Yet one common practice in Sub Saharan Africa is the prescription of antimicrobials and the parallel consumption of widely used traditional medicines which are potentially harmful to the liver (Riebensahm et al., 2019).

Western drugs which have so far attracted attention as potential treatment options for COVID-19 include chloroquine **(158)**, hydroxychloroquine **(159)**, azithromycin **(160)**, ceftriaxone **(161)** (for patients with pneumonia), remdesivir **(162)**, favipiravir **(163)**, ribavirin **(164)**, lopinavir–ritonavir **(165)** (used in combination) and of recent dexamethasone that have been shown to reduce mortality rate of COVID- 19 patients. The CYP enzymes such as CYP2C8, CYP3A4, CYP2D6 and CYP1A1 can metabolize chloroquine (Spaldin et al., 1994; Projean, 2003a; Kim et al., 2003; Gil and Gil Berglund, 2007) and catalyzes the dealkylation of chloroquine **(158)** and hydroxychloroquine **(159)** to pharmacologically active compound (McChesney, 1983; Spaldin et al., 1994; Furst, 1996; Projean, 2003b) CYP3A4 is responsible for the metabolism of Dexamethasone (DEX) **(166)** to 6-hydroxyDEX (6OH-DEX) **(167)** (Tomlinson et al., 1997). Remdesivir **(162)** metabolism is mediated by hydrolases, however, it has been shown to exert weak inhibitory effects on CYP3A4, OATP1B1, OATP1B3, bile acid export pump, multidrug resistance-associated protein (Sciences, 2020), and sodium-taurocholate cotransporter protein. It is also established that remdesivir **(162)** is a substrate of CYP2C8, CYP2D6, CYP3A4, OATP1B1, OATP1B3, bile acid export pump, multidrug resistance-associated protein (European Medicines Agency, 2020), and sodium-taurocholate cotransporter protein. Lopinavir and ritonavir **(165)** therapy strongly induces CYP2C19 activity and mildly induces CYP1A2 and CYP2C9. Both intestinal and hepatic CYP3A are inhibited by lopinavir and ritonavir therapy, the former being greatly affected (Yeh et al., 2006). The metabolism of favipiravir **(163)** is mediated by aldehyde oxidase (AO) and xanthine oxidase in the hepatocyte cytosol, and not by CYP450 enzymes. But there are reports which support favipiravir **(163)** as an inhibitor of CYP2C8 (Madelain et al., 2016).

Several widely distributed African medicinal plants with long history in therapeutic use, are employed in the management of different infectious diseases **(** Tables 1–3 **)** and many of them are being repurposed for COVID-19 by herbal firms and local healthcare providers in Africa. Some of such plants e.g *Artemisia* plant species including *Artemisia abrotanum* L. (Asteracea), *Artemisia caruifolia* Buch. -Ham. ex Roxb (Asteracea), *Artemisia pontica L.* (Asteracea), *Artemisia herba-alba* Asso (Compositae)*, Artemisia absinthium* L. (Asteracea), *Artemisia afra* Jacq (Asteraceae) significantly inhibits CYP3A4 (Lazaridi, 2014). Aqueous infusions (3.3 mg/L) of *Artemisia annua* displays significant reduction in the CYP3A4 activity (Lazaridi, 2014). Many of the plants applied in TAM accumulate bioactive polyunsaturated fatty acids (PUFA) such as linoleic **(75)**, linolenic **(74)**, docosahexaenic acid **(67)** which have been reported to have profound inhibitory effect on CYP3A4. Linoleic **(75)** and linolenic acid **(74)** are the most common acids found in plants of the *Artemisia* family. *Artemisia annua* is the main active component of the claimed anti-COVID-19 herbal formulation popularly called COVID organics by Madagascar. In another study, 75, 52 and 5% of CYP3A4 were respectively inhibited by100 μg/mL grapefruit oil, Eucalyptol **(77)** and menthol **(65)** (Zhang and Lim, 2008). Curcumin **(72)** (40 µM), 6-gingerol **(79)** (100 µM), citral **(78)** (250 µM), d-limonene **(64)** (400 µM), β-caryophyllene **(66)** (500 µM), 1,8-cineole **(77)** (1 mM), myrcene (1 mM) shows inhibitory effect on CYP3A4; piperine **(76)** has been shown to enhance bioavailability (Lutgen, 2016). These bioactive plant metabolites which have demonstrated antiviral properties have been reported in several plants used in TAM for the symptomatic treatment of COVID-19 in Nigeria (Table 1 and Figure 2). This means the co-administration of these herbal medicines with orthodox drugs used in COVID-19 management could result in herb-drug interaction that potentially may be hepatotoxic. However, this area of study has not received adequate scientific attention, particularly by *in vivo* experiments and should be of research interest in future.

*Garcinia kola* (bitter kola) is widely used in TAM, the West African sub-region in particular, for a host of conditions including infectious diseases and management of upper respiratory infections (Buba et al., 2016). GARCINIA-IHP, used for symptoms of cold and sore throat, pains, cough, nasal congestion, viral infections and inflammation, is one of the herbal health products marketed by InterceDD Health Products in Nigeria. This herbal formulation is currently under consideration as a repurposed remedy in COVID-19 management after successful clinical trials. As a result of the radical media promotion of claims on *Garcinia kola* in COVID-19 prevention and management which lacks scientific evidence, the seed of the plant has become one of the many other local herbal recipes consumed without any control by a majority of people in West Africa. The correct doses of bitter kola to achieve the best therapeutic effects and without any adverse side effects are often not followed. However, scientific report suggests that *Garcinia kola* induces CYP3A4 transcription by a multiple of 3.7-factors in HepG2 cells at 90 μmol/L (Nwankwo et al., 2000) increasing the possibility for herb-drug interaction. Future investigations will therefore focus on the extensive *in vitro* and *in vivo* anti-SARS CoV-2 activities of extracts of *Garcinia kola*, in depth toxicity studies and a holistic investigation of a possible herb-drug interaction.

*Allium* species (*Allium cepa* and *Allium sativum*) are widely used in Traditional African medicines for the management of infectious diseases and has since been regularly canvassed by some of the COVID-19 infected users in West Africa for the prevention and symptomatic management of COVID-19. These species are among the recipes recommended by the traditional leadership of the Yoruba ethnic nationality in Nigeria and are claimed to be efficacious in COVID-19 prevention and “cure”. Garlic oil, obtained from *A. sativum* bulb contains sulfur-compounds such as diallyl sulfide **(69)** (DAS), diallyl disulfide **(70)** (DADS), and diallyl trisulfide **(71)** (DATS) which induce CYP2B and NAD(P)H quinone oxidoreductase 1 (NQO1). DAS **(69)** facilitate the induction of CYP2B10 mRNA and also activate human CYP2B6 and NQO1 promoters which are primarily regulated by constitutive androstane receptor (CAR) and nuclear factor E2-related factor 2 (Nrf2) transcription factors, respectively (Fisher et al., 2007). Further investigation is therefore needed to unveil the mechanisms of possible herb-drug interaction.

### Pharmacokinetic Considerations in Developing Potential anti-COVID-19 Herbal Medicines

The popular school of thought tends toward the discovery of a single metabolite specific for one macromolecular target. However, modern medicine via the formulation of multi-component medications containing two, three, or more active components is increasingly accepting the limits of the single-molecule hypothesis. Such multi-component medications are of special importance in anti-infective therapies, and in fact have become the obligatory standard of management in malaria, a protozoan infection, tuberculosis a bacterial infection, and acquired immune deficiency syndrome a viral infection. The overarching aim is to employ the combined drug compounds to target multiple macromolecular targets.

Such multi-component systems natively form a core aspect of plant-derived preparations which range from crude extracts, to carefully designed fractional combinations, and to a lower extent pure natural products. Since most component active principles exist in lower amounts than found in mainstream pharmaceuticals, toxicities from these preparations are generally rarer especially when prepared using properly validated quality assurance processes or following some local preparation methods. Additionally benefit results from the presence of multiple natural products capable of modulating multiple aspects of the biochemical process of interest, a property that is of special interest in antiviral and antimalarial management. Interestingly, some of these botanicals have been suggested to produce strong biological effects even at the low concentrations at which they are present in herbal preparations (Johanna et al., 2012). Together with their beneficial ability to prevent resistance development, herb-based preparations should preferably form a core component of the search for treatment of the current COVID-19 pandemic.

Since the pharmacodynamic effects resulting from herb-based preparations eventually depend on the component principles interacting with biological macromolecules, their successful use also depends critically on pharmacokinetics. With respect to the pharmacokinetics of herbal products, the simultaneous presence of multiple structurally distinct natural products in the same preparation presents a definite layer of challenge not seen with single compound pharmaceuticals but that is often of clinical significance (Fugh-Berman, 2000). Each natural product present in such multi-component preparations possess physicochemical attributes that are often divergent such that it is hardly realistic to describe the overall pharmacokinetics based on a single natural product however significant such compound may be. Instead, it is sometimes more prudent to define these parameters for the bulk product based on an all-or-none basis rather than merely trying to extrapolate the properties from data derived for individual natural products. This was aptly demonstrated in a 2013 study showing the strong CYP3A4-inhibiting activity of an African herbal preparation NIPRD-AM1 with antimalarial activities (Bulus Adzu et al., 2013). NIPRD-AM1 was developed in Nigeria and found to be responsible for diminishing by half the enzymatic activity of the cytochrome P450 enzyme, but following co-administration with metronidazole it was reported to exert no effect on the metabolic disposition of the drug (Obodozie et al., 2011). It is certainly not inconceivable to expect to find present individual natural products with varied effects on the hepatic enzymes in such herbal formulations as NIPRD-AM1; it is however the overall effect of the mixture that is of practical importance for quality assurance as well as for clinical applicability especially involving the analysis of interference with different metabolizing enzymes and with the absorption process of drugs that are likely to be co-administered.

Absorption and bioavailability profiling is challenging for herbal mixtures, partly because existing mathematical models can only suitably describe single drug molecules. The AUC and bioavailability for example, are referenced against measured systemic concentrations of individual drugs and can only describe one drug substance at a time. This however is not to downplay the importance of equivalent means of profiling herb-based preparations. In fact, many natural products are associated with deficient molecular properties compromising solubility, membrane permeation and thus absorption and bioavailability. This is however not surprising since the evolutionary process that emerged the natural products inside plants is isolated from processes within the human biological system where a fine balance of molecular features is often required for receptor interaction. Natural products present in plants have no such evolutionary imperative beyond the functions natively played in their host plants. Therefore, a good number of natural products with good *in vitro* biological profiles have been reported with suboptimal physicochemical properties. Interestingly both the pharmacological potency and poor absorption properties of the component natural products are closely tied with their complex chemical structures. Polyphenols are a class of phytochemicals whose immunomodulatory properties can be useful in herbal preparations for COVID19. They are however often characterized by poor absorption and deactivation by gastric conditions (D’Archivio et al., 2010). In the same category is the group of curcuminoids whose antiviral, anti-inflammatory and antioxidant properties should ordinarily qualify as viable components of anti-COVID19 treatment. However, curcuminoids **(72)** are associated with high logP values that are generally above 3.0 for the monomeric forms like curcumin and turmeric, and logP values higher than 7.0 for the dimer forms: this together with the high molecular weight associated with dimerized curcuminoids **(72)** associate these compounds with low bioavailability. In spite of its established diverse pharmacological activities (Sreejayan and Rao, 1996; Goel et al., 2001; Chainani-Wu, 2003; Xu et al., 2008; Mathy-Hartert et al., 2009; Henrotin et al., 2010; Shakibaei et al., 2011; Zhang et al., 2016), only trace amounts of curcumin **(72)** were found in the systemic circulation following oral administration (Bisht et al., 2007). This has expectedly limited clinical applications. And by extension, poor pharmacokinetics *in vivo* may hide otherwise potent anti-SARS-CoV2 preparations and such considerations should be factored in designing anti-SARS-CoV2 activity exploration experiments.

A positive aspect of the use of herb-based preparations in pharmacotherapy is the observation that the derivable pharmacological benefits often emanate from multiple constituent natural products that are sufficiently structurally diverse and yet related to permit the modulation of varied and yet related biochemical processes. It is crucial to point out that the anti-coronavirus activities identified for these natural products were obtained by *in vitro* experiments often involving enzyme-based inhibition assays against isolated viral enzymes. In the absence of biological membranes and the complexity associated within vivo systems, pharmacokinetics is thus unlikely to constitute any real challenge. On the other hand, had these natural products or their multi-component herbal preparations been tested in *in vivo* situations, they might have been thought to lack the anti-coronavirus activities with which they have been credited. Pharmacokinetic factors and experimental design are thus crucial considerations in the identification of potent anti-SARS-CoV2 herb-based products, and it is recommended that testing should in the first stages employ enzyme-based assays while physicochemical/pharmacokinetic liabilities can later be remedied by employing appropriate formulation techniques.

## Conclusion

In this review, several in silico, *in vitro* and few *in vivo* studies have revealed the therapeutic potential of plant-derived bioactive compounds for the treatment of viral infections in TAM, but the data are too preliminary to be adopted in clinical settings. We have shown clearly that available data are not sufficient to encourage the clinical use of TAM against viral infections, COVID-19 in this special case. Despite the huge potential of TAM, one big and notable gap in knowledge of antiviral application of TAM is the lack of well documented human studies with comparative data. This calls for more research effort geared toward the clinical application of TAM during viral epidemics. Among the primary and secondary metabolites documented, polysaccharides, lectins, cyclotides, alkaloids, flavonoids, tannins and terpenes have been widely exploited and studied for the treatment of several viral diseases and virtually tested for efficacy against SARS-CoV-2. However, only a few of these identified compounds such as KB1, KB8 and andrographolide have good scientific evidence; others require a more elaborate evidence while quite a few have evidence that are rather inconclusive or outright poor quality evidence. For instance, the cyclotide Kalata B1 and B8 from *O. affinis* have been supported by a good evidence for antiviral (anti-HIV) as well as immunostimulating activities in addition to their well-documented oral bioavailability, target specificity, low toxicity, desirable stability in body fluid, mechanism of activity and the uterotonic potential of KB1. Homologues of these knottin peptide therapeutics have recently been identified in some endemic tropical African plants including *Rinorea dentata and Rinorea oblongifolia,* thus expanding their natural sources *in planta*.

The emergence of the deadly COVID-19 pandemic has demonstrated the extent of (over 85%) dependence of African populations on medicinal plants for primary healthcare needs. Traditional Chinese Medicine (TCM), which is currently on a progressive pre-clinical and clinical standardization path, has proved to be effective during the outbreak of SARS-CoV and now SARS-CoV-2 as TCM has been well integrated to western medicines with outcomes supporting their continued use and integration. Africa can follow this same path to evidence-based application of indigenous phytomedicines in the prevention and treatment of viral diseases including COVID-19. Findings from this review indicates that extensive clinical studies are urgently needed to evaluate their therapeutic efficacy and adverse effects especially herb-drug interaction. Importantly, the identification of commercially available herbal medicines used in TAM as documented in this review, which have received an initial regulatory approval for use in humans, and active against viruses, might accelerate their repurposed considerations, observational applications, clinical trials and eventual clinical use, particularly during sudden outbreaks of highly pathogenic viruses like the SARS COV-2. However, future research should equally investigate their mechanism of activity in order to improve their formulation, antiviral activity and to reduce the risk of side effects.

While few of the anti-COVID 19 herbal claims are now undergoing scientific investigation for efficacy and safety, many of the claims are content-secretive making it difficult to validate scientifically destroying the prospect for their clinical application. Although no indigenous phytomedicine has been scientifically validated and authorized for use to prevent or treat COVID- 19, yet there is a growing interest in traditional medicines as potential remedies for COVID-19 in Africa. As a follow up, the World Health Organization (WHO) and the Africa Centers for disease Control and Prevention (Africa CDC) launched a 25-member Regional Expert Committee on Traditional Medicine for COVID-19 to support countries in a collaborative effort to conduct clinical trials on traditional medicines in compliance with international standards.

Phytomedicines used in Traditional African Medicines are most often sourced from the wild and less often cultivated, serves over 85% of the populations within the region, yet have not received utmost respect for good clinical practice (GCP) during the production process. Plants used in Traditional African Medicine are, until now substantially and unsustainably collected from the wild. The tropical forests, including the Sub-Saharan tropical African flora, which covers up to half of the world's angiospermic plants have been described to be in danger of a continuous decline at an estimated 16.8 million ha/annum (Purvis et al., 2019). This riotous and unabated competition for the unsustainable depletion of plant resource in Africa which are most often collected for medicinal and non-medicinal purposes constitutes a threat to plant biodiversity and their availability to future generations. Thus, the sustainable use of medicinal plant resource in Africa should encourage their purpose-driven cultivation for use in African ethnomedicine as well as for production of evidence-led phytomedicines. In addition, the establishment of requisite programs for medicinal plant resource utilization and conservation of endemic African plants are opportunities for future studies. This has become urgent as plant cultivation offers pharmacological advantages over collection from the wild due to variation in quality and composition resulting from environmental and genomic mutations (WHO et al., 1993). Furthermore, cultivated plants reduces the possibility for variation in addition to the unlikely event of getting the therapeutic benefit. More so, the sustainable use of plants via cultivation has the potential of reducing the likelihood of adulteration and wrong identification. The efficacy and toxicity assessment of phytomedicines used in Africa for the prevention and treatment of COVID-19 is urgently needed to justify or discourage their local uses. According to WHO Africa*,* “*even if therapies are derived from traditional practice and natural, establishing their efficacy and safety through rigorous clinical trials is critical*”. Therefore, future research should focus on an indepth scientific investigation to demystify the bioactive component and establish chemical fingerprint for such complex herbal phytomedicines and mechanistically study them for an evidence-rooted and verifiable plant-derived medicines with potential for COVID 19 management. Consequently, the integration of Traditional African Medicine into the western-based national healthcare structure and clinical study of potential herb-drug interaction are research outlook for consideration by research institutions in Africa and the government of African countries.

## References

[B1] Abedini A. Roumy V. Mahieux S. Biabiany M. Standaert-Vitse A. Rivière C. BailleulSahpaz F. Neut C. Hennebelle T. (2013). Rosmarinic acid and its methyl ester as antimicrobial components of the hydromethanolic extract of Hyptis atrorubens Poit. (Lamiaceae). Evid. Based Complement. Alternat Med. 2013, 604536. 10.1155/2013/604536 24348709 PMC3855952

[B2] Ablordeppey S. Hufford C. Borne R. Dwuma-Badu D. (1990). 1H-NMR and13C-NMR assignments of cryptolepine, A 3:4-Benz-δ-carboline derivative isolated fromCryptolepis sanguinolenta. Planta Med. 56 (4), 416–417. 10.1055/s-2006-960998 17221441

[B3] Adebayo J. O. Adewole K. E. Krettli A. U. (2017). Cysteine-stabilised peptide extract of *Morinda* lucida (Benth) leaf exhibits antimalarial activity and augments antioxidant defense system in P. berghei- infected mice. J. Ethnopharmacology 207, 118–128. 10.1016/j.jep.2017.06.026 28645782

[B4] Adepiti A. O. Elujoba A. A. Bolaji O. O. (2014). *In vivo* antimalarial evaluation of MAMA decoction on Plasmodium berghei in mice. Parasitol. Res. 113 (2), 505–511. 10.1007/s00436-013-3680-0 24271081

[B5] Afolayan F. I. D. Adegbolagun O. Mwikwabe N. N. Orwa J. Anumudu C. (2020). Cytokine modulation during malaria infections by some medicinal plants. Scientific Afr. 8, e00428. 10.1016/j.sciaf.2020.e00428

[B6] Africa CDC (2020). World Health Organisation, Africa CDC in joint push for COVID-19 traditional medicine research in Africa. Geneva, Switzerland: World Health Organization.

[B7] Ahmad S. Abbasi H. W. Shahid S. Gul S. Abbasi S. W. (2020). Molecular docking, simulation and MM-PBSA studies of nigella sativa compounds: a computational quest to identify potential natural antiviral for COVID-19 treatment. J. Biomol. Struct. Dyn. 12, 1–9. 10.1080/07391102.2020.1775129 PMC7298883 32462996

[B8] Akin-Osanaiya B. C. Nok A. J. Ibrahim S. Inuwa H. M. Onyike E. Amlabu E. (2013). Antimalarial effect of neem leaf and neem stem bark extracts on Plasmodium berghei infected in the pathology and treatment of malaria. Int. J. Res. Biochem. Biophys. 3 (1), 7–14.

[B9] Akram M. Hamid A. Khalil A. Ghaffar A. Tayyaba N. Saeed A. Naveed A. (2014). Review on medicinal uses, pharmacological, phytochemistry and immunomodulatory activity of plants. Int. J. Immunopathol. Pharmacol. 27 (3), 313–319. 10.1177/039463201402700301 25280022

[B10] Ali B. H. Blunden G. (2003). Pharmacological and toxicological properties of Nigella sativa. Phytother. Res. 17 (4), 299–305. 10.1002/ptr.1309 12722128

[B11] Aracil A. Green J. (2019). Plants with antimalarial properties: a systematic review of the current clinical evidence. Eur. J. Integr. Med. 28, 76–85. 10.1016/j.eujim.2019.04.005

[B12] Arunasree K. M. (2010). Anti-proliferative effects of carvacrol on a human metastatic breast cancer cell line, MDA-MB 231. Phytomedicine 17 (8–9), 581–588. 10.1016/j.phymed.2009.12.008 20096548

[B13] Asante D.-B. Henneh I. T. Acheampong D. O. Kyei F. Adokoh C. K. Ofori E. G. (2019). Anti-inflammatory, anti-nociceptive and antipyretic activity of young and old leaves of Vernonia amygdalina. Biomed. Pharmacother. 111, 1187–1203. 10.1016/j.biopha.2018.12.147 30841432

[B14] Asres K. Bucar F. Kartnig T. Witvrouw M. Pannecouque C. De Clercq E. (2001). Antiviral activity against human immunodeficiency virus type 1 (HIV-1) and type 2 (HIV-2) of ethnobotanically selected Ethiopian medicinal plants. Phytother. Res. 15 (1), 62–69. 10.1002/1099-1573(200102)15:1<62::aid-ptr956>3.0.co;2-x 11180526

[B15] Attah A. F. Hellinger R. Sonibare M. A. Moody J. O. Arrowsmith S. Wray S. (2016b). Ethnobotanical survey of Rinorea dentata (Violaceae) used in South-Western Nigerian ethnomedicine and detection of cyclotides. J. Ethnopharmacology 179, 83–91. 10.1016/j.jep.2015.12.038 PMC5858781 26721222

[B16] Attah A. F. Sonibare M. A. Moody J. O. (2016a). Chemical detection of cysteine-rich circular petides in selected tropical Violaceae and Moringaceae families using modified G-250 and mass spectrometry. Niger. J. Nat. Prod. Med. 20, 88–95.

[B17] Awortwe C. Bouic P. J. Masimirembwa C. M. Rosenkranz B. (2013). Inhibition of major drug metabolizing CYPs by common herbal medicines used by HIV/AIDS patients in Africa-- implications for herb-drug interactions. Drug Metab. Lett. 7 (2), 83–95. 10.2174/1872312808666140129123210 PMC4354708 24475926

[B18] Azizi Z. Ebrahimi S. Saadatfar E. Kamalinejad M. Majlessi N. (2012). Cognitive-enhancing activity of thymol and carvacrol in two rat models of dementia. Behav. Pharmacol. 23 (3), 241–249. 10.1097/fbp.0b013e3283534301 22470103

[B19] Badam L. Joshi S. P. Bedekar S. S. (1999). “*In vitro*” antiviral activity of neem (Azadirachta indica. A. Juss) leaf extract against group B coxsackieviruses. J. Commun. Dis. 31 (2), 79–90. 10810594

[B20] Badani H. Garry R. F. Wimley W. C. (2014). Peptide entry inhibitors of enveloped viruses: the importance of interfacial hydrophobicity. Biochim. Biophys. Acta - Biomembranes 1838 (9), 2180–2197. 10.1016/j.bbamem.2014.04.015 PMC7094693 24780375

[B21] Badary O. A. Taha R. A. Gamal El-Din A. M. Abdel-Wahab M. H. (2003). Thymoquinone is a potent superoxide anion scavenger. Drug Chem. Toxicol. 26 (2), 87–98. 10.1081/dct-120020404 12816394

[B22] Badia-Boungou F. Sane F. Alidjinou E. K. Hennebelle T. Roumy V. Ngakegni-Limbili A. C. (2019). Aqueous extracts of Syzygium brazzavillense can inhibit the infection with coxsackievirus B4 *in vitro* . J. Med. Virol. 91 (7), 1210–1216. 10.1002/jmv.25436 30788849

[B23] Banzouzi J.-T. Prado R. Menan H. Valentin A. Roumestan C. Mallié M. (2004). Studies on medicinal plants of Ivory Coast: investigation of *Sida* acuta for *in vitro* antiplasmodial activities and identification of an active constituent. Phytomedicine 11 (4), 338–341. 10.1078/0944711041495245 15185848

[B24] Barku V. Y. A. Opoku-Boahen Y. Dzotsi E. Y. (2012). Isolation and pharmacological activities of alkaloids from Cryptolepis sanguinolenta (Lindl) schlt. Int. Res. J. Biochem. Bioinform 2, 58–61.

[B25] Barnes J. Anderson L. A. Gibbons S. PhillipsonBarnes J. D. J. Anderson L. A. Gibbons S. (2005). Echinaceaspecies (*Echinacea angustifolia*(DC.) hell., *Echinacea pallida*(Nutt.) nutt., *Echinacea purpurea*(L.) Moench): a review of their chemistry, pharmacology and clinical properties. J. Pharm. Pharmacol. 57 (8), 929–954. 10.1211/0022357056127 16102249

[B26] Bekerman E. Einav S. (2015). Infectious disease. Combating emerging viral threats. Science 348 (6232), 282–283. 10.1126/science.aaa3778 25883340 PMC4419706

[B27] Berit T. Mulder L. M. Mayra D. T. Smit J. M. (2020). Tomatidine, a natural steroidal alkaloid shows antiviral activity towards chikungunya virus *in vitro* . Sci. Rep. (Nature Publ Group). 10 (1), 6364. 10.1038/s41598-020-63397-7 PMC7156627 32286447

[B28] Bisht S. Feldmann G. Soni S. Ravi R. Karikar C. Maitra A. (2007). Polymeric nanoparticle-encapsulated curcumin ("nanocurcumin"): a novel strategy for human cancer therapy. J. Nanobiotechnol 5 (1), 3. 10.1186/1477-3155-5-3 PMC1868037 17439648

[B29] Biswas, D. Nandy S. Mukherjee A. Pandey D. K. Dey A. (2020). Moringa oleifera Lam. and derived phytochemicals as promising antiviral agents: a review. South Afr. J. Bot. 129, 272–282. 10.1016/j.sajb.2019.07.049

[B30] Biswas K. Chattopadhyay I. Banerjee R. K. Bandyopadhyay U. (2020b). Biological activities and medicinal properties of neem (Azadirachta indica). Curr. Sci. 82 (11), 1336–1345.

[B31] Boas L. C. P. Campos M. L. Berlanda R. L. A. de Carvalho Neves N. Franco O. L. (2019). Antiviral peptides as promising therapeutic drugs. Cell Mol Life Sci 76 (18), 3525–3542. 10.1007/s00018-019-03138-w 31101936 PMC7079787

[B32] Bordoloi M. Barua N. C. Ghosh A. C. (1996). An artemisinic acid analogue from Tithonia diversifolia. Phytochemistry 41, 557–559. 10.1016/0031-9422(95)00569-2

[B33] Borkotoky S. Banerjee M. (2020). A computational prediction of SARS-CoV-2 structural protein inhibitors from Azadirachta indica (Neem). J. Biomol. Struct. Dyn., 1–11. 10.1080/07391102.2020.1774419 PMC7311162 32462988

[B34] Borquaye L. S. Gasu E. N. Ampomah G. B. Kyei L. K. Amarh M. A. Mensah C. N. (2020). Alkaloids from cryptolepis sanguinolenta as potential inhibitors of SARS-CoV-2 viral proteins: an in silico study. Biomed. Res. Int. 2020, 1. 10.1155/2020/5324560 PMC7512045 33029513

[B35] Bouckaert J. Poortmans F. Wyns L. Loris R. (1996). Sequential structural changes upon zinc and calcium binding to metal-free concanavalin A. J. Biol. Chem. 271 (27), 16144–16150. 10.1074/jbc.271.27.16144 8663112

[B36] Brahmachari G. Gorai D. Roy R. (2013). Argemone mexicana: chemical and pharmacological aspects. Rev. Bras. de Farmacog. 23 (3), 559–575. 10.1590/s0102-695x2013005000021

[B37] Bringmann G. Messer K. Schwöbel B. Brun R. Aké Assi L. (2003). Habropetaline A, an antimalarial naphthylisoquinoline alkaloid from Triphyophyllum peltatum. Phytochemistry 62 (3), 345–349. 10.1016/s0031-9422(02)00547-2 12620347

[B38] Buba C. I. Okhale S. E. Muazzam I. (2016). *Garcinia* kola: the phytochemistry, pharmacology and therapeutic applications. Int. J. Pharmacogn 3 (2), 67–81. 10.13040/IJPSR.0975-8232.IJP.3(2).67-81

[B39] Bulugahapitiya V. P. Arachchige P. K. (2012). Preliminary study on cytotoxic compounds from the seeds of Nigella sativa L. (Black cumin). Ruhuna J. Sci. 1 (1). 10.4038/rjs.v1i0.74

[B40] Bulus Adzu K. B. M. Mustapha K. B. Masimirembwa C. Obodozie O. Kirim R. A. Gamaniel K. S. (2013). Simulation of metabolism-based herb-drug interaction: towards safe and efficacious use of NIPRD-AM1. Avicenna J. Phytomed 3 (3), 201. 25050275 PMC4075716

[B41] Carlucci M. Scolaro L. Errea M. Matulewicz M. Damonte E. (1997). Antiviral activity of natural sulphated galactans on herpes virus multiplication in cell culture. Planta Med. 63, 429–432. 10.1055/s-2006-957727 9342947

[B341] Cascella M. Rajnik M. Cuomo A. Dulebohn S. C. Di Napoli R. (2021). Features, evaluation, and treatment of coronavirus (COVID-19). Statpearls [internet]. 32150360

[B42] Chainani-Wu N. (2003). Safety and anti-inflammatory activity of curcumin: a component of tumeric (Curcuma longa). J. Altern. Complement. Med. 9 (1), 161–168. 10.1089/107555303321223035 12676044

[B43] Challand S. Willcox M. (2009). A clinical trial of the traditional MedicineVernonia amygdalinain the treatment of uncomplicated malaria. J. Altern. Complement. Med. 15, 1231–1237. 10.1089/acm.2009.0098 19922255

[B44] Chan J. F.-W. Kok K.-H. Zhu Z. Chu H. To K. K.-W. Yuan S. (2020a). Genomic characterization of the 2019 novel human-pathogenic coronavirus isolated from a patient with atypical pneumonia after visiting Wuhan. Emerg. Microbes and Infect. 9 (1), 221–236. 10.1080/22221751.2020.1719902 31987001 PMC7067204

[B45] Chan J. F.-W. Yuan S. Kok K.-H. To K. K.-W. Chu H. Yang J. (2020b). A familial cluster of pneumonia associated with the 2019 novel coronavirus indicating person-to-person transmission: a study of a family cluster. Lancet 395 (10223), 514–523. 10.1016/S0140-6736(20)30154-9 31986261 PMC7159286

[B46] Chang Y.-C. Tung Y.-A. Lee K.-H. Chen T.-F. Hsiao Y.-C. Chang H.-C. (2020). Potential therapeutic agents for COVID-19 based on the analysis of protease and RNA polymerase docking. Preprints (February), 1–7. 10.20944/preprints202002.0242.v1

[B47] Chen B. Colgrave M. L. Daly N. L. Rosengren K. J. Gustafson K. R. Craik D. J. (2005). Isolation and characterization of novel cyclotides from viola hederaceae. J. Biol. Chem. 280 (23), 22395–22405. 10.1074/jbc.m501737200 15824119

[B48] Chinedu E. Arome D. Ameh S. (2014). African herbal plants used as anti-malarial agents-A review. PharmaTutor 2 (3), 47–53.

[B49] Chodur G. M. Olson M. E. Wade K. L. Stephenson K. K. Nouman W. Fahey J. W. (2018). Wild and domesticated Moringa oleifera differ in taste, glucosinolate composition, and antioxidant potential, but not myrosinase activity or protein content. Sci. Rep. 8 (1). 10.1038/s41598-018-26059-3 PMC5964143 29789671

[B50] Choi H. J. Song J. H. Park K. S. Baek S. H. (2010). In vitroanti-enterovirus 71 activity of gallic acid fromWoodfordia fruticosaflowers. LettApplMicrobiol 50, 438–440. 10.1111/j.1472-765x.2010.02805.x 20149083

[B51] Chrubasik S. Pittler M. H. Roufogalis B. D. (2005). Zingiberis rhizoma: a comprehensive review on the ginger effect and efficacy profiles. Phytomedicine 12 (9), 684–701. 10.1016/j.phymed.2004.07.009 16194058

[B52] Chukwujekwu J. C. Smith P. Coombes P. H. Mulholland D. A. Van Staden J. (2005). Antiplasmodial diterpenoid from the leaves of Hyptis suaveolens. J. Ethnopharmacology 102 (2), 295–297. 10.1016/j.jep.2005.08.018 16213121

[B53] Cimanga K. De Bruyne T. Apers S. Pieters L. Totté J. Kambu K. (1999). Complement-inhibiting constituents of Bridelia ferruginea stem bark. Planta Med. 65 (3), 213–217. 10.1055/s-1999-14059 17260306

[B55] Colunga Biancatelli R. M. L. Berrill M. Catravas J. D. Marik P. E. (2020). Quercetin and vitamin C: an experimental, synergistic therapy for the prevention and treatment of SARS-CoV-2 related disease (COVID-19). Front. Immunol. 11, 11. 10.3389/fimmu.2020.01451 32636851 PMC7318306

[B57] Coriolano M. C. de Santana Brito J. de Siqueira Patriota L. L. de Araujo Soares A. K. de Lorena V. M. B. Paiva P. M. G. (2018). Immunomodulatory effects of the water-soluble lectin from Moringa oleifera seeds (WSMoL) on human peripheral blood mononuclear cells (PBMC). Ppl 25 (3), 295–301. 10.2174/0929866525666180130141736 29384049

[B58] Cos P. Hermans N. De Bruyne T. Apers S. Sindambiwe J. B. Vanden Berghe D. (2002a). Further evaluation of Rwandan medicinal plant extracts for their antimicrobial and antiviral activities. J. Ethnopharmacology 79 (2), 155–163. 10.1016/S0378-8741(01)00362-2 11801376

[B59] Cos P. Hermans N. De Bruyne T. Apers S. Sindambiwe J. B. Witvrouw M. (2002b). Antiviral activity of Rwandan medicinal plants against human immunodeficiency virus type-1 (HIV-1). Phytomedicine 9 (1), 62–68. 10.1078/0944-7113-00083 11924766

[B60] Cruz G. V. B. Pereira P. V. S. Patrício F. J. Costa G. C. Sousa S. M. Frazão J. B. (2007). Increase of cellular recruitment, phagocytosis ability and nitric oxide production induced by hydroalcoholic extract from *Chenopodium* ambrosioides leaves. J. Ethnopharmacology 111 (1), 148–154. 10.1016/j.jep.2006.11.006 17156956

[B61] Cunningham A. B. (1997). An Africa-wide overview of medicinal plant harvesting. Med. Plants Conserv Heal Care 92, 116.

[B62] da Silva Antonio A. Wiedemann L. S. M. Veiga-Junior V. F. (2020). Natural products’ role against COVID-19. RSC Adv. 10 (39), 23379–23393. 10.1039/d0ra03774e PMC9122563 35693131

[B63] Daly N. L. Gustafson K. R. Craik D. J. (2004). The role of the cyclic peptide backbone in the anti-HIV activity of the cyclotide kalata B1. FEBS Lett. 574 (1–3), 69–72. 10.1016/j.febslet.2004.08.007 15358541

[B64] Daly N. L. Koltay A. Gustafson K. R. Boyd M. R. Casas-Finet J. R. Craik D. J. (1999). Solution structure by NMR of circulin A: a macrocyclic knotted peptide having anti-HIV activity 1 1Edited by P. E. Wright. J. Mol. Biol. 285 (1), 333–345. 10.1006/jmbi.1998.2276 9878410

[B65] Danaher R. J. Wang C. Dai J. Mumper R. J. Miller C. S. (2011). Antiviral effects of blackberry extract against herpes simplex virus type 1. Oral Surg. Oral Med. Oral Pathol. Oral Radiol. Endod. 112, e31–e35. 10.1016/j.tripleo.2011.04.007 PMC3154751 21827957

[B66] Dao T. T. Nguyen P. H. Lee H. S. Kim E. Park J. Lim S. I. (2011). Chalcones as novel influenza A (H1N1) neuraminidase inhibitors from *Glycyrrhiza* inflata. Bioorg. Med. Chem. Lett. 21, 294–298. 10.1016/j.bmcl.2010.11.016 21123068

[B67] Das A. (2015). Anticancer effect of antimalarial artemisinin compounds. Ann. Med. Health Sci. Res. 5 (2), 93–102. 10.4103/2141-9248.153609 25861527 PMC4389338

[B68] Das G. Ghosh S. Garg S. Ghosh S. Jana A. Samat R. (2020). An overview of key potential therapeutic strategies for combat in the COVID-19 battle. RSC Adv. 10 (47), 28243–28266. 10.1039/d0ra05434h PMC9127683 35685027

[B69] De Leo A. Arena G. Lacanna E. Oliviero G. Colavita F. Mattia E. (2012). Resveratrol inhibits Epstein Barr Virus lytic cycle in Burkitt's lymphoma cells by affecting multiple molecular targets. Antiviral Res. 96, 196–202. 10.1016/j.antiviral.2012.09.003 22985630

[B70] de Veer S. J. Kan M.-W. Craik D. J. (2019). Cyclotides: from structure to function. Chem. Rev. 119 (24), 12375–12421. 10.1021/acs.chemrev.9b00402 31829013

[B71] Dharmaratne H. R. W. Tan G. T. Marasinghe G. P. K. Pezzuto J. M. (2002). Inhibition of HIV-1 reverse transcriptase and HIV-1 replication by *Calophyllum* coumarins and xanthones. Planta Med. 68, 86–87. 10.1055/s-2002-20058 11842340

[B72] Divneet Kaur N. K. (2019). A comprehensive review on phytochemistry and pharmacological activities of Vernonia amygdalina. J. Pharmacogn Phytochem. 8 (3), 2629–2636.

[B73] Donma M. M. Donma O. (2020). The effects of Allium sativum on immunity within the scope of COVID-19 infection. Med. Hypotheses 144, 109934. 10.1016/j.mehy.2020.109934 32512493 PMC7265825

[B74] Dzoyem J. P. Tshikalange E. Kuete V. (2013). Medicinal plants market and industry in Africa. Med. Plant Res. Africa Pharmacol. Chem., 859–890. 10.1016/B978-0-12-405927-6.00024-2

[B75] D’Archivio M. Filesi C. Varì R. Scazzocchio B. Masella R. (2010). Bioavailability of the polyphenols: status and controversies. Int. J. Mol. Sci. 11 (4), 1321–1342. 10.3390/ijms11041321 20480022 PMC2871118

[B76] El-Dakhakhny M. (1965). Studies on the Egyptian Nigella sativa L. IV. Some pharmacological properties of the seeds' active principle in comparison to its dihydro compound and its polymer. Arzneimittelforschung 15, 1227–1229. 4380349

[B77] Elujoba A. A. Odeleye O. M. Ogunyemi C. M. (2005). Traditional medicine development for medical and dental primary health care delivery system in Africa. Afr. J Tradit Complement Altern Med 2 (1), 46–61.

[B78] Esimone C. O. Grunwald T. Wildner O. Nchinda G. Tippler B. Proksch P. (2005). *In vitro* pharmacodynamic evaluation of antiviral medicinal plants using a vector-based assay technique. J. Appl. Microbiol. 99 (6), 1346–1355. 10.1111/j.1365-2672.2005.02732.x 16313407

[B79] Esquenazi D. Wigg M. D. Miranda M. M. F. S. Rodrigues H. M. Tostes J. B. F. Rozental S. (2002). Antimicrobial and antiviral activities of polyphenolics from Cocos nucifera Linn. (Palmae) husk fiber extract. Res. Microbiol. 153 (10), 647–652. 10.1016/s0923-2508(02)01377-3 12558183

[B80] European Medicines Agency (2020). Summary on compassionate use remdesivir gilead. Eur. Med. Agency 3 (April), 41 Available at: https://www.ema.europa.eu/en/documents/other/summary-compassionate-use-remdesivir-gilead_en.pdf.

[B81] Fabricant D. S. Farnsworth N. R. (2001). The value of plants used in traditional medicine for drug discovery. Environ. Health Perspect. 109 (Suppl. 1), 69–75. 10.1289/ehp.01109s169 11250806 PMC1240543

[B82] Faccin-Galhardi L. C. Aimi Yamamoto K. Ray S. Ray B. Carvalho Linhares R. E. Nozawa C. (2012). The *in vitro* antiviral property of Azadirachta indica polysaccharides for poliovirus. J. Ethnopharmacology 142 (1), 86–90. 10.1016/j.jep.2012.04.018 22855945

[B83] Fahey J. W. Wade K. L. Stephenson K. K. Shi Y. Liu H. Panjwani A. A. (2019). A strategy to deliver precise oral doses of the glucosinolates or isothiocyanates from moringa oleifera leaves for use in clinical studies. Nutrients 11 (7), 1547. 10.3390/nu11071547 PMC6682957 31323988

[B84] Ferrea G. Canessa A. Sampietro F. Cruciani M. Romussi G. Bassetti D. (1993). *In vitro* activity of a Combretum micranthum extract against herpes simplex virus types 1 and 2. Antiviral Res. 21 (4), 317–325. 10.1016/0166-3542(93)90010-g 8215303

[B85] Fialho L. G. da Silva V. P. Reis S. R. N. I. Azeredo E. L. Kaplan M. A. C. Figueiredo M. R. (2016). Antiviral and immunomodulatory effects of norantea brasiliensis choisy on dengue virus-2. Intervirology 59 (4), 217–227. 10.1159/000455855 28329744

[B86] Fisher C. D. Augustine L. M. Maher J. M. Nelson D. M. Slitt A. L. Klaassen C. D. (2007). Induction of drug-metabolizing enzymes by garlic and allyl sulfide compounds via activation of constitutive androstane receptor and nuclear factor E2-related factor 2. Drug Metab. Dispos 35 (6), 995–1000. 10.1124/dmd.106.014340 17353348

[B87] Fugh-Berman A. (2000). Herb-drug interactions. The Lancet 355, 134–138. 10.1016/s0140-6736(99)06457-0 10675182

[B88] Furst D. E. (1996). Pharmacokinetics of hydroxychloroquine and chloroquine during treatment of rheumatic diseases. Lupus 5 (Suppl. 1), 11–15. 10.1177/0961203396005001041 8803904

[B89] Gamaniel K. (2009). A comparative randomized clinical trial of NIPRD AM1 against a chloroquine and sulphadoxine/pyrimethamine combination in symptomatic but uncomplicated malaria. Afr. J. Tradit Complement. Altern. Med. 6, 411–412.

[B90] Gao S. Ying M. Yang F. Zhang J. Yu C. (2020). Zhang Boli: traditional Chinese medicine plays a role in the prevention and treatment on novel coronavirus pneumonia. in Open access online-first publ res pap COVID-19, Geneva, Switzerland: WHO. 121–124. Available at: http://en.gzbd.cnki.net/GZBT/brief/Default.aspx.

[B91] Ghildiyal R. Prakash V. Chaudhary V. K. Gupta V. Gabrani R. (2020). Phytochemicals as antiviral agents: recent updates. in Plant-derived bioactives. Singapore: Springer, 279–295.

[B92] Ghonmode W. N. Balsaraf O. D. Tambe V. H. Saujanya K. P. Patil A. K. Kakde D. D. (2013). Comparison of the antibacterial efficiency of neem leaf extracts, grape seed extracts and 3% sodium hypochlorite against *E. feacalis* - an *in vitro* study. J. Int. Oral Health 5 (6), 61. 24453446 PMC3895719

[B93] Gil J. Gil Berglund E. (2007). CYP2C8 and antimalaria drug efficacy. Pharmacogenomics 8, 187–198. 10.2217/14622416.8.2.187 17286541 PMC7117598

[B94] Goel A. Boland C. R. Chauhan D. P. (2001). Specific inhibition of cyclooxygenase-2 (COX-2) expression by dietary curcumin in HT-29 human colon cancer cells. Cancer Lett. 172 (2), 111–118. 10.1016/s0304-3835(01)00655-3 11566484

[B95] Goren A. Goldman W. Trainin Z. Goldman S. (2003). US Pat Appl. No 10/076 247.

[B96] Goyal B. R. Goyal R. K. Mehta A. A. (2007). PHCOG rev.: plant review phyto-pharmacology of *Achyranthes aspera*: a review. Pharmacogn Rev. 1 (1).

[B97] Gran L. Sandberg F. Sletten K. (2000). Oldenlandia affinis (R&S) DC. J. Ethnopharmacology 70 (3), 197–203. 10.1016/s0378-8741(99)00175-0 10837983

[B98] Gründemann C. Koehbach J. Huber R. Gruber C. W. (2013). Do plant cyclotides have potential as immunosuppressant peptides?. J. Nat. Prod. 75 (2), 167–174. 10.1021/np200722w PMC3399773 22272797

[B99] Guo Y.-R. Cao Q.-D. Hong Z.-S. Tan Y.-Y. Chen S.-D. Jin H.-J. (2020). The origin, transmission and clinical therapies on coronavirus disease 2019 (COVID-19) outbreak - an update on the status. Mil. Med Res 7 (1), 1–10. 10.1186/s40779-020-00240-0 32169119 PMC7068984

[B100] Gyebi G. A. Ogunro O. B. Adegunloye A. P. Ogunyemi O. M. Afolabi S. O. (2020). Potential inhibitors of coronavirus 3-chymotrypsin-like protease (3CLpro): an in silico screening of alkaloids and terpenoids from African medicinal plants. J. Biomol. Struct. Dyn., 1–13. 10.1080/07391102.2020.1764868 PMC7256353 32367767

[B101] Haid S. Novodomská A. Gentzsch J. Grethe C. Geuenich S. Bankwitz D. (2012). A plant-derived flavonoid inhibits entry of all HCV genotypes into human hepatocytes. Gastroenterology 143 (1), 213–222. 10.1053/j.gastro.2012.03.036 22465429

[B102] Haidara M. Bourdy G. De Tommasi N. Braca A. Traore K. Giani S. (2016). Medicinal plants used in Mali for the treatment of malaria and liver diseases. Nat. Product. Commun. 11 (3), 1934578X1601100. 10.1177/1934578x1601100309 27169180

[B103] Hamidi J. A. Ismaili N. H. Ahmadi F. B. Lajisi N. H. (1996). Antiviral and cytotoxic activities of some plants used in Malaysian indigenous medicine. Pertanika J. Trop. Agric. Sci. 19 (2203), 129–136.

[B104] Han S. B. Kim Y. H. Lee C. W. Park S. M. Lee H. Y. Ahn K. S. (1998). Characteristic immunostimulation by angelan isolated from Angelica gigas Nakai. Immunopharmacology 40 (1), 39–48. 10.1016/S0162-3109(98)00026-5 9776477

[B105] Harata K. Muraki M. (2000). Crystal structures of Urtica dioica agglutinin and its complex with tri-N-acetylchitotriose. J. Mol. Biol. 297 (3), 673–681. 10.1006/jmbi.2000.3594 10731420

[B106] Haudecoeur R. Peuchmaur M. Pérès B. Rome M. Taïwe G. S. Boumendjel A. (2018). Traditional uses, phytochemistry and pharmacological properties of African Nauclea species: a review. J. Ethnopharmacology 212, 106–136. 10.1016/j.jep.2017.10.011 29045823

[B107] Haładyj E. Sikora M. Felis-Giemza A. Olesińska M. (2018). Antimalarials - are they effective and safe in rheumatic diseases?. Reumatologia 56 (3), 164–173. 10.5114/reum.2018.76904 30042604 PMC6052376

[B108] He X. Fang J. Guo Q. Wang M. Li Y. Meng Y. (2020). Advances in antiviral polysaccharides derived from edible and medicinal plants and mushrooms. Carbohydr. Polym. 229, 115548. 10.1016/j.carbpol.2019.115548 31826474

[B109] Henrotin Y. Clutterbuck A. L. Allaway D. Lodwig E. M. Harris P. Mathy-Hartert M. (2010). Biological actions of curcumin on articular chondrocytes. Osteoarthritis and Cartilage 18 (2), 141–149. 10.1016/j.joca.2009.10.002 19836480

[B110] Hensel A. Bauer R. Heinrich M. Spiegler V. Kayser O. Hempel G. (2020). Challenges at the time of COVID-19: opportunities and innovations in antivirals from nature. Planta Med. 86 (10), 659. 10.1055/a-1177-4396 32434254 PMC7356065

[B111] Heredia A. Davis C. Redfield R. (2000). Synergistic inhibition of HIV-1 in activated and resting peripheral blood mononuclear cells, monocyte-derived macrophages, and selected drug-resistant isolates with nucleoside analogues combined with a natural product, resveratrol. JAIDS J. Acquired Immune Deficiency Syndromes 25, 246–255. 10.1097/00126334-200011010-00006 11115955

[B112] Houghton P. Zarka R. de las Heras B. Hoult J. (1995). Fixed oil ofNigella sativaand derived thymoquinone inhibit eicosanoid generation in leukocytes and membrane lipid peroxidation. Planta Med. 61 (1), 33–36. 10.1055/s-2006-957994 7700988

[B113] Hudson J. Graham E. Chan G. Finlayson A. Towers G. (1986). Comparison of the antiviral effects of naturally occurring thiophenes and polyacetylenes. Planta Med. 52, 453–457. 10.1055/s-2007-969252 3562663

[B114] Huisman M. M. H. Fransen C. T. M. Kamerling J. P. Vliegenthart J. F. G. Schols H. A. Voragen A. G. J. (2001). The CDTA-soluble pectic substances from soybean meal are composed of rhamnogalacturonan and xylogalacturonan but not homogalacturonan. Biopolymers 58 (3), 279–294. 10.1002/1097-0282(200103)58:3<279::aid-bip1005>3.0.co;2-1 11169388

[B115] Iheagwam F. N. Israel E. N. Kayode K. O. DeCampos O. C. Ogunlana O. O. Chinedu S. N. (2020). Nauclea latifolia Sm. Leaf extracts extenuates free radicals, inflammation, and diabetes-linked enzymes. Oxidative Med Cell Longevity 2020, 5612486. 10.1155/2020/5612486 PMC7085881 32256953

[B116] Ireland D. C. Wang C. K. L. Wilson J. A. Gustafson K. R. Craik D. J. (2008). Cyclotides as natural anti-HIV agents. Biopolymers 90 (1), 51–60. 10.1002/bip.20886 18008336 PMC6296364

[B117] Islam S. N. Begum P. Ahsan T. Huque S. Ahsan M. (2004). Immunosuppressive and cytotoxic properties of Nigella sativa. Phytother Res. 18 (5), 395–398. 10.1002/ptr.1449 15174000

[B118] Iwu M. M. (2014). Handbook of African medicinal plants. Boca Raton, FL: CRC Press.

[B119] Jacobson J. M. Feinman L. Liebes L. Ostrow N. Koslowski V. Tobia A. (2001). Pharmacokinetics, safety, and antiviral effects of hypericin, a derivative of st. John’s wort plant, in patients with chronic hepatitis C virus infection. Antimicrob. Agents Chemother. 45, 517–524. 10.1128/aac.45.2.517-524.2001 11158749 PMC90321

[B120] Jacques A. S. Arnaud S. S. Fr&ejus O. O. Jacques D. T. (2020). Review on biological and immunomodulatory properties of Moringa oleifera in animal and human nutrition. J. Pharmacogn Phyther 12 (1), 1–9.

[B121] Jain S. Arora P. Popli H. (2020). A comprehensive review on Citrus aurantifolia essential oil: its phytochemistry and pharmacological aspects. Braz. J. Nat. Sci. 3 (2), 354. 10.31415/bjns.v3i2.101

[B122] James E. S. Adolfina R. K. Acquaye D. Elton J. S. Rodolfo J. R. G. Janick J. (2007). Medicinal crops of Africa. in Issues in new crops and new uses. Ashcroft, BC, Canada: ASHS Press.

[B123] Jassim S. A. A. Naji M. A. (2003). Novel antiviral agents: a medicinal plant perspective. J. Appl. Microbiol. 95 (3), 412–427. 10.1046/j.1365-2672.2003.02026.x 12911688

[B124] Jasso-Miranda C. Herrera-Camacho I. Flores-Mendoza L. K. Dominguez F. Vallejo-Ruiz V. Sanchez-Burgos G. G. (2019). Antiviral and immunomodulatory effects of polyphenols on macrophages infected with dengue virus serotypes 2 and 3 enhanced or not with antibodies. Idr Vol. 12, 1833–1852. 10.2147/idr.s210890 PMC6611719 31303775

[B125] Jiang Z.-Y. Liu W.-F. Zhang X.-M. Luo J. Ma Y.-B. Chen J.-J. (2013). Anti-HBV active constituents from Piper longum. Bioorg. Med. Chem. Lett. 23, 2123–2127. 10.1016/j.bmcl.2013.01.118 23434420

[B126] Johanna M. Wrulich O. A. Jenny M. Fuchs D. Ueberall F. (2012). An update on the strategies in multicomponent activity monitoring within the phytopharmaceutical field. BMC Complement. Altern. Med. 12, 18. 10.1186/1472-6882-12-18 22417247 PMC3359261

[B127] Jose R. J. Manuel A. (2020). COVID-19 cytokine storm: the interplay between inflammation and coagulation. Lancet Respir. Med. 395, 1054. 10.1016/S2213-2600(20)30216-2 PMC7185942 32353251

[B128] Jung M. Lee S. Kim H. Kim H. (2012). Recent studies on natural products as anti-HIV agents. Cmc 7 (6), 649–661. 10.2174/0929867003374822 10702631

[B129] Kacem R. Meraihi Z. (2006). Effects of essential oil extracted from Nigella sativa (L.) seeds and its main components on human neutrophil elastase activity. Yakugaku Zasshi 126 (4), 301–305. 10.1248/yakushi.126.301 16596021

[B130] Kapoor R. Sharma B. Kanwar S. S. (2017). Antiviral phytochemicals: an overview. Biochem. Physiol. 6 (2), 7.

[B131] Kaur N. Dhuna V. Kamboj S. S. Agrewala J. Singh J. (2006). A novel antiproliferative and antifungal lectin from *Amaranthus* viridis Linn seeds. Ppl 13 (9), 897–905. 10.2174/092986606778256153 17100645

[B132] Khan A. Chen H. C. Tania M. Zhang D. Z. (2011). Anticancer activities of Nigella sativa (black cumin). Afr. J Tradit Complement Altern Med 8 (5S). 10.4314/ajtcam.v8i5s.10 PMC3252704 22754079

[B133] Kim H. J. Yu Y. G. Park H. Lee Y. S. (2002). HIV gp41 Binding Phenolic Components fromFraxinus sieboldianavar.angustata. Planta Med. 68 (11), 1034–1036. 10.1055/s-2002-35665 12451497

[B134] Kim K.-A. Park J.-Y. Lee J.-S. Lim S. (2003). Cytochrome P450 2C8 and CYP3A4/5 are involved in chloroquine metabolism in human liver microsomes. Arch. Pharm. Res. 26, 631–637. 10.1007/bf02976712 12967198

[B135] Kini S. G. Wong K. H. Tan W. L. Xiao T. Tam J. P. (2017). Morintides: cargo-free chitin-binding peptides from Moringa oleifera. BMC Plant Biol. 17 (1), 68. 10.1186/s12870-017-1014-6 28359256 PMC5374622

[B136] Komlaga G. Cojean S. Beniddir M. A. Loiseau P. M. (2015). The antimalarial potential of three Ghanaian medicinal plants.

[B137] Kong L. Li S. Liao Q. Zhang Y. Sun R. Zhu X. (2013). Oleanolic acid and ursolic acid: novel hepatitis C virus antivirals that inhibit NS5B activity. Antiviral Res. 98, 44–53. 10.1016/j.antiviral.2013.02.003 23422646

[B138] Kotwal G. J. (2008). Genetic diversity-independent neutralization of pandemic viruses (e.g. HIV), potentially pandemic (e.g. H5N1 strain of influenza) and carcinogenic (e.g. HBV and HCV) viruses and possible agents of bioterrorism (variola) by enveloped virus neutralizing compounds (EVNCs). Vaccine 26, 3055–3058. 10.1016/j.vaccine.2007.12.008 18241960

[B139] Kraft C. Jenett-Siems K. Siems K. Jakupovic J. Mavi S. Bienzle U. (2003). *In vitro* antiplasmodial evaluation of medicinal plants from Zimbabwe. Phytother. Res. 17, 123–128. 10.1002/ptr.1066 12601673

[B140] Krawitz C. Mraheil M. A. Stein M. ImirzaliogluC D. E. (2011). Inhibitory activity of a standardized elderberry liquid extract against clinically relevant human respiratory bacterial pathogens and influenza A and B viruses. BMC Complement. Altern. Med. 11, 16. 10.1186/1472-6882-11-16 21352539 PMC3056848

[B141] Kumar V. P. Prashanth K. V. H. Venkatesh Y. P. (2015). Structural analyses and immunomodulatory properties of fructo-oligosaccharides from onion ( Allium cepa ). Carbohydr. Polym. 117, 115–122. 10.1016/j.carbpol.2014.09.039 25498616

[B142] Kumar V. P. Venkatesh Y. P. (2016). Alleviation of cyclophosphamide-induced immunosuppression in Wistar rats by onion lectin ( Allium cepa agglutinin). J. Ethnopharmacol 186, 280–288. 10.1016/j.jep.2016.04.006 27063982

[B143] Kurokawa M. Shimizu T. Watanabe W. Shiraki K. (2010). Development of new antiviral agents from natural products∼!2010-01-17∼!2010-04-12∼!2010-08-27∼!. Toantimj 2 (2), 49–57. 10.2174/1876518101002020049

[B144] Laforge M. Elbim C. Frère C. Hémadi M. Massaad C. Nuss P. (2020). Tissue damage from neutrophil-induced oxidative stress in COVID-19. Nat. Rev. Immunol. 20 (9), 515–516. 10.1038/s41577-020-0407-1 32728221 PMC7388427

[B145] Lamorde M. Tabuti J. R. S. Obua C. Kukunda-Byobona C. Lanyero H. Byakika-Kibwika P. (2010). Medicinal plants used by traditional medicine practitioners for the treatment of HIV/AIDS and related conditions in Uganda. J. Ethnopharmacology 130 (1), 43–53. 10.1016/j.jep.2010.04.004 20451595

[B146] Landa P. Kokoska L. Pribylova M. Vanek T. Marsik P. (2009). *In vitro* anti-inflammatory activity of carvacrol: inhibitory effect on COX-2 catalyzed prostaglandin E2 biosynthesisb. Arch. Pharm. Res. 32 (1), 75–78. 10.1007/s12272-009-1120-6 19183879

[B147] Lazaridi K. (2014). Invloed van de chemische samenstelling van *Artemisia* annua op CYP3A4-activiteit en antioxidant vermogen. Masterproef voorgelegd tot het verkrijgen van de graad van Apotheker. Academiejaar.

[B148] Lee C.-L. Chiang L.-C. Cheng L.-H. Liaw C.-C. Abd El-Razek M. H. Chang F.-R. (2009). Influenza A (H1N1) antiviral and cytotoxic agents fromFerula assa-foetida. J. Nat. Prod. 72, 1568–1572. 10.1021/np900158f 19691312

[B149] Lee-Huang S. Huang P. L. Chen H. C. Huang P. L. Bourinbaiar A. Huang H. I. (1995). Anti-HIV and anti-tumor activities of recombinant MAP30 from bitter melon. Gene 161, 151. 7665070 10.1016/0378-1119(95)00186-a

[B150] Leung P.-C. (2007). The efficacy of Chinese medicine for SARS: a review of Chinese publications after the crisis. Am. J. Chin. Med. 35 (04), 575–581. 10.1142/s0192415x07005077 17708624

[B151] Li S. Chen C. Zhang H. Guo H. Wang H. Wang L. (2005). Identification of natural compounds with antiviral activities against SARS-associated coronavirus. Antiviral Res. 67 (1), 18–23. 10.1016/j.antiviral.2005.02.007 15885816 PMC7114104

[B152] Li Z. Hua C. Pan X. Fu X. Wu W. (2016). Carvacrol exerts neuroprotective effects via suppression of the inflammatory response in middle cerebral artery occlusion rats. Inflammation 39 (4), 1566–1572. 10.1007/s10753-016-0392-5 27324156

[B153] Liang L. Wang C. Li S. Chu X. Sun K. (2019). Nutritional compositions of Indian Moringa oleifera seed and antioxidant activity of its polypeptides. Food Sci. Nutr. 7 (5), 1754–1760. 10.1002/fsn3.1015 31139388 PMC6526633

[B154] Lima E. B. C. Sousa C. N. S. Meneses L. N. Ximenes N. C. Santos Júnior M. A. Vasconcelos G. S. (2015). Cocos nucifera (L.) (Arecaceae): a phytochemical and pharmacological review. Braz. J. Med. Biol. Res. 48 (11), 953–964. 10.1590/1414-431x20154773 26292222 PMC4671521

[B155] Lima M. S. Quintans-Junior L. J. Alcântara de Santana W. Kaneto C. M. Soares M. B. P. Villarreal C. F. (2013). Eur. J. Pharmacol. 699 (1-3), 112–117. 23220159 10.1016/j.ejphar.2012.11.040

[B156] Lin F. L. Wu S. J. Lee S. C. Ng L. T. (2009). Antioxidant, antioedema and analgesic activities ofAndrographis paniculataextracts and their active constituent andrographolide. Phytother. Res. 23 (7), 958–964. 10.1002/ptr.2701 19142986

[B157] Lin S. Liu M. T. Wang S. J. Li S. Yang Y. C. Shi J. G. (2008). [Coumarins from branch of Fraxinus sieboldiana and their antioxidative activity]. Zhongguo Zhong Yao Za Zhi 33 (14), 1708–1710. 18841773

[B158] Lin S. Wang S. Liu M. Gan M. Li S. Yang Y. (2007). Glycosides from the stem bark ofFraxinussieboldiana. J. Nat. Prod. 70 (5), 817–823. 10.1021/np0700467 17461599

[B159] Lone S. A. Ahmad A. (2020). COVID-19 pandemic - an African perspective. Emerg. Microbes Infect. 9, 1300–1308. 10.1080/22221751.2020.1775132 32458760 PMC7473237

[B160] Loschwitz J. Jäckering A. Keutmann M. Olagunju M. Eberle R. J. Coronado M. A. (2020). Novel inhibitors of the main protease of SARS-CoV-2 identified via a molecular dynamics simulation-guided *in Vitro* assay. ChemRxiv 2020. 10.26434/chemrxiv.13200281.v1 PMC8007184 33862474

[B161] Luo H. Tang Q. L. Shang Y. X. Liang S. B. Yang M. Robinson N. (2020). Can Chinese medicine be used for prevention of corona virus disease 2019 (COVID-19)? A review of historical classics, research evidence and current prevention programs. Chin. J. Integr. Med., 1–8. 10.1007/s11655-020-3192-6 PMC7088641 32065348

[B162] Lutgen P. (2016). *Artemisia*, CYP3A4 and scopoletin. MalariaWorld.

[B163] Madelain V. Nguyen T. H. T. Olivo A. De Lamballerie X. Guedj J. Taburet A.-M. (2016). Ebola virus infection: review of the pharmacokinetic and pharmacodynamic properties of drugs considered for testing in human efficacy trials. Clin. Pharmacokinet. 55 (8), 907–923. 10.1007/s40262-015-0364-1 26798032 PMC5680399

[B164] Madzuki I. N. Lau S. F. Abdullah R. Mohd Ishak N. I. Mohamed S. (2019). Vernonia amygdalina inhibited osteoarthritis development by anti‐inflammatory and anticollagenase pathways in cartilage explant and osteoarthritis‐induced rat model. Phytotherapy Res. 33 (7), 1784–1793. 10.1002/ptr.6366 31033070

[B165] Mahmoud M. R. El-Abhar H. S. Saleh S. (2002). The effect of Nigella sativa oil against the liver damage induced by Schistosoma mansoni infection in mice. J. Ethnopharmacology 79 (1), 1–11. 10.1016/s0378-8741(01)00310-5 11744288

[B166] Mahomoodally M. F. (2013). Traditional medicines in Africa: an appraisal of ten potent African medicinal plants. Evid. Based Complement. Alternat Med. 2013, 617459. 10.1155/2013/617459 24367388 PMC3866779

[B167] Malik S. Hasan S. S. Choudhary M. I. Ni C. Z. Clardy J. (1995). Nigellidine—a new indazole alkaloid from the seeds of Nigella sativa. Tetrahedron Lett. 36 (12), 1993–1996.

[B168] Malik S. Zaman K. (1992). Nigellimine: a new isoquinoline alkaloid from the seeds of Nigella sativa. J. Nat. Prod. 55 (5), 676–678.

[B169] Marchetti M. Mastromarino P. Rieti S. Seganti L. Orsi N. (1995). Inhibition of herpes simplex, rabies and rubella viruses by lectins with different specificities. Res. Virol. 146 (3), 211–215. 10.1016/0923-2516(96)80581-4 7481093

[B170] Maroyi A. (2014). Alternative medicines for HIV/AIDS in resource-poor settings: insight from traditional medicines use in sub- saharan Africa. Trop. J. Pharm. Res. 13 (9), 1527–1536. 10.4314/tjpr.v13i9.21

[B171] Martins F. O. Esteves P. F. Mendes G. S. Barbi N. S. Menezes F. S. (2009). Verbascoside isolated from Lepechiniaspeciosa has inhibitory activity against HSV-1 and HSV-2 *in vitro* . Nat. Prod. Commun. 4, 1693–1696. 10.1177/1934578x0900401217 20120109

[B172] Mathy-Hartert M. Jacquemond-Collet I. Priem F. Sanchez C. Lambert C. Henrotin Y. (2009). Curcumin inhibits pro-inflammatory mediators and metalloproteinase-3 production by chondrocytes. Inflamm. Res. 58 (12), 899–908. 10.1007/s00011-009-0063-1 19579007

[B173] McChesney E. W. (1983). Animal toxicity and pharmacokinetics of hydroxychloroquine sulfate. Am. J. Med. 75, 11–18. 10.1016/0002-9343(83)91265-2 6408923

[B174] McFadden R. Peterson N. (2011). Interactions between drugs and four common medicinal herbs. Nurs. Stand. 25, 65–68. 10.7748/ns.25.19.65.s54 21287929

[B175] Meagher J. L. Winter H. C. Ezell P. Goldstein I. J. Stuckey J. A. (2005). Crystal structure of banana lectin reveals a novel second sugar binding site. Glycobiology 15 (10), 1033–1042. 10.1093/glycob/cwi088 15944373

[B176] Mehrbod P. Abdalla M. A. Fotouhi F. Heidarzadeh M. Aro A. O. Eloff J. N. (2018b). Immunomodulatory properties of quercetin-3-O-α-L-rhamnopyranoside from Rapanea melanophloeos against influenza a virus. BMC Complement. Altern. Med. 18 (1), 1–10. 10.1186/s12906-018-2246-1 29903008 PMC6003079

[B177] Mehrbod P. Abdalla M. A. Njoya E. M. Ahmed A. S. Fotouhi F. Farahmand B. (2018a). South African medicinal plant extracts active against influenza A virus. BMC Complement. Altern. Med. 18 (1), 1–10. 10.1186/s12906-018-2184-y 29587734 PMC5872571

[B178] Mehrbod P. Ebrahimi S. N. Fotouhi F. Eskandari F. Eloff J. N. McGaw L. J. (2019). Experimental validation and computational modeling of anti-influenza effects of quercetin-3-O-α-L-rhamnopyranoside from indigenous south African medicinal plant Rapanea melanophloeos. BMC Complement. Altern. Med. 19 (1), 1–11. 10.1186/s12906-019-2774-3 31791311 PMC6888925

[B179] Memvanga P. B. Tona G. L. Mesia G. K. Lusakibanza M. M. Manzo.R. K. Cimanga R. K. (2015). Antimalarial activity of medicinal plants from the Democratic Republic of Congo: a review. J. Ethnopharmacology 169, 76–98. 10.1016/j.jep.2015.03.075 25862959

[B180] Mesia K. Cimanga K. Tona L. Mampunza M. M. Ntamabyaliro N. Muanda T. (2011). Assessment of the short-term safety and tolerability of a quantified 80 % ethanol extract from the stem bark of Nauclea pobeguinii (PR 259 CT1) in healthy volunteers: a clinical phase I study. Planta Med. 77, 111–116. 10.1055/s-0030-1250134 20665369

[B181] Mesia K. Tona L. Mampunza M. Ntamabyaliro N. Muanda T. Muyembe T. (2012b). Antimalarial efficacy of a quantified extract ofNauclea pobeguiniiStem bark in human adult volunteers with diagnosed uncomplicated falciparum malaria. Part 1: a clinical phase IIA trial. Planta Med. 78, 211–218. 10.1055/s-0031-1280359 22095262

[B182] Mesia K. Tona L. Mampunza M. Ntamabyaliro N. Muanda T. Muyembe T. (2012a). Antimalarial efficacy of a quantified extract of Nauclea pobeguinii stem bark in human adult volunteers with diagnosed uncomplicated falciparum malaria. Part 2: a clinical phase IIB trial. Planta Med. 78, 853–860. 10.1055/s-0031-1298488 22538476

[B183] Michelini F. M. Alché L. E. Bueno C. A. (2018). Virucidal, antiviral and immunomodulatory activities of β-escin and Aesculus hippocastanum extract. J. Pharm. Pharmacol. 70 (11), 1561–1571. 10.1111/jphp.13002 30168142

[B184] Mills E. Foster B. C. Heeswijk R. v. Phillips E. Wilson K. Leonard B. (2005). Impact of African herbal medicines on antiretroviral metabolism. AIDS 19, 95–97. 10.1097/00002030-200501030-00013 15627040

[B185] Mitchell C. A. Ramessar K. O'Keefe B. R. (2017). Antiviral lectins: selective inhibitors of viral entry. Antiviral Res. 142, 37–54. 10.1016/j.antiviral.2017.03.007 28322922 PMC5414728

[B186] Monzingo A. F. Collins E. J. Ernst S. R. Irvin J. D. Robertus J. D. (1993). The 2·5 Å structure of pokeweed antiviral protein. J. Mol. Biol. 233 (4), 705–715. 10.1006/jmbi.1993.1547 8411176

[B187] Mouhajir F. Hudson J. B. Rejdali M. Towers G. H. N. (2001). Multiple antiviral activities of endemic medicinal plants used by Berber peoples of Morocco. Pharm. Biol. 39 (5), 364–374. 10.1076/phbi.39.5.364.5892

[B188] Moura M. C. Trentin D. S. Napoleão T. H. Primon-Barros M. Xavier A. S. Carneiro N. P. (2017). Multi-effect of the water-solubleMoringa oleiferalectin againstSerratia marcescensandBacillussp.: antibacterial, antibiofilm and anti-adhesive properties. J. Appl. Microbiol. 123 (4), 861–874. 10.1111/jam.13556 28792661

[B189] Mukherjee H. Ojha D. Bag P. Chandel H. S. Bhattacharyya S. Chatterjee T. K. (2013). Anti-herpes virus activities of *Achyranthes aspera*: an Indian ethnomedicine, and its triterpene acid. Microbiol. Res. 168 (4), 238–244. 10.1016/j.micres.2012.11.002 23218996

[B190] Murata T. Miyase T. Muregi F. W. Naoshima-Ishibashi Y. Umehara K. Warashina T. MkojiKanou G. M. Terada M. Ishih A. (2008). Antiplasmodial triterpenoids fromEkebergia capensis. J. Nat. Prod. 71 (2), 167–174. 10.1021/np0780093 18220356

[B191] Naseer S. Hussain S. Naeem N. Pervaiz M. Rahman M. (2018). The phytochemistry and medicinal value of Psidium guajava (guava). Clin. Phytoscience 4 (1), 1–8. 10.1186/s40816-018-0093-8

[B192] National Center for Biotechnology Information (2020). PubChem compound summary for CID 11610052.

[B193] Nawrot R. Barylski J. Nowicki G. Broniarczyk J. Buchwald W. Goździcka-Józefiak A. (2014). Plant antimicrobial peptides. Folia Microbiol. 59 (3), 181–196. 10.1007/s12223-013-0280-4 24092498 PMC3971460

[B194] Neurath A. R. Strick N. Li Y. Y. D. A. (2004). Punicagranatum (Pomegranate) juice provides an HIV-1 entry inhibitor and candidate topical microbicide. BMC Infect. Dis. 4, 41. 10.1186/1471-2334-4-41 15485580 PMC533885

[B195] Neurath A. R. Strick N. Li Y. Y. D. A. (2005). Punica granatum (pomegranate) juice provides an HIV-1 entry inhibitor and candidate topical microbicide. Ann. New York Acad. Sci. 1056, 311–327. 10.1196/annals.1352.015 16387698

[B196] Neuwinger H. D. (2000). African traditional medicine: a dictionary of plant use and applications. With supplement: search system for diseases. Medpharm.

[B197] Nguyen K. N. T. Nguyen G. K. T. Nguyen P. Q. T. Ang K. H. Dedon P. C. Tam J. P. (2016). Immunostimulating and Gram-negative-specific antibacterial cyclotides from the butterfly pea (Clitoria ternatea). FEBS J. 283 (11), 2067–2090. 10.1111/febs.13720 27007913

[B198] Nie L.-x. Wu Y.-l. Dai Z. Ma S.-c. (2020). Antiviral activity of Isatidis Radix derived glucosinolate isomers and their breakdown products against influenza A *in vitro*/ovo and mechanism of action. J. Ethnopharmacology 251, 112550. 10.1016/j.jep.2020.112550 PMC7126217 31918015

[B199] Nwankwo J. O. Tahnteng J. G. Emerole G. O. (2000). Inhibition of aflatoxin Bl genotoxicity in human liver-derived HepG2 cells by kolaviron biflavonoids and molecular mechanisms of action. Eur. J. Cancer Prev. 9 (5), 351–362. 10.1097/00008469-200010000-00010 11075889

[B200] Nworu C. S. Ejikeme T. I. Ezike A. C. Ndu O. Akunne T. C. Onyeto C. A. (2017). Anti-plasmodial and anti-inflammatory activities of cyclotide-rich extract and fraction of Oldenlandia affinis (R. & S.) D.C. (Rubiaceae). Afr. H. Sci. 17 (3), 827–843. 10.4314/ahs.v17i3.26 PMC5656185 29085411

[B201] Obodozie O. O. Ebeshi B. U. Mustapha K. B. Kirim R. A. Margaret Ekpenyong U. S. I. (2011). The effects of an investigational antimalarial agent, NIPRD-AM1 on the single dose pharmacokinetics of metronidazole in healthy human volunteers. Eur. J. Drug Metab. Pharmacokinet. 35, 103–108. 10.1007/s13318-010-0012-y 21302036

[B202] Odediran S. A. Elujoba A. A. Adebajo A. C. (2014). Influence of formulation ratio of the plant components on the antimalarial properties of MAMA decoction. Parasitol. Res. 113, 1977–1984. 10.1007/s00436-014-3848-2 24658658

[B204] Ogbole O. O. Adeniji J. A. Ajaiyeoba E. O. Adu D. F. (2013). Anti-polio virus activity of medicinal plants selected from the Nigerian ethno-medicine. Acad. Journals 12 (24), 3878–3883.

[B205] Ogbole O. O. Akinleye T. E. Segun P. A. Faleye T. C. Adeniji A. J. (2018). *In vitro* antiviral activity of twenty-seven medicinal plant extracts from Southwest Nigeria against three serotypes of echoviruses. Virol. Journal 15 (1), 110. 10.1186/s12985-018-1022-7 PMC6052623 30021589

[B206] Olubiyi O. O. Olagunju M. Keutmann M. Loschwitz J. Strodel B. (2020). High throughput virtual screening to discover inhibitors of the main protease of the coronavirus sars-cov-2. Molecules 25, 3193. 10.3390/molecules25143193 PMC7396980 32668701

[B207] Omoregie E. S. Pal A. (2016). Antiplasmodial, antioxidant and immunomodulatory activities of ethanol extract of Vernonia amygdalina del. Leaf in Swiss mice. Avicenna J. Phytomed 6 (2), 236. 27222837 PMC4877969

[B208] Osadebe P. Omeje E. (2007). Isolation and characterisation of antiviral and immunomodulatory constituents from Nigerian mistletoe, Loranthus micranthus. Planta Med. 73 (9), 108. 10.1055/s-2007-986890

[B309] Osipiuk J. Jedrzejczak R. Tesar C. Endres M. Stols L. Babnigg G. (2020). The crystal structure of papain-like protease of SARS CoV-2. RSCB PDB.

[B209] Ozerov I. A. Zhinkina N. A. Efimov A. M. Machs E. M. Rodionov A. V. (1994). Antimicrobial activity of bark extracts of Bridelia ferruginea (Euphorbiaceae). J. Ethnopharmacol 43, 185–190. 7990492 10.1016/0378-8741(94)90041-8

[B210] Panraksa P. Ramphan S. Khongwichit S. Smith D. R. (2017). Activity of andrographolide against dengue virus. Antiviral Res. 139, 69–78. 10.1016/j.antiviral.2016.12.014 28034742

[B211] Parvez M. K. Al‐Dosari M. S. Alam P. Rehman M. Alajmi M. F. Alqahtani A. S. (2019). The anti‐hepatitis B virus therapeutic potential of anthraquinones derived from Aloe vera. Phytotherapy Res. 33 (11), 2960–2970. 10.1002/ptr.6471 31410907

[B212] Pascolutti M. Quinn R. J. (2014). Natural products as lead structures: chemical transformations to create lead-like libraries. Drug Discov. Today 19, 215. 10.1016/j.drudis.2013.10.013 24171951

[B213] Patil L. Kulkarni K. Khanvilkar V. (2014). *In vitro* evaluation of herb-drug interactions: a review. Int. J. Pharm. Pharm. Sci. 6, 9–12.

[B214] Paul B. D. Subba Rao G. Kapadia G. J. (1974). Isolation of myricadiol, myricitrin, taraxerol, and taraxerone from Myrica cerifera L. root bark. J. Pharm. Sci. 63 (6), 958–959. 10.1002/jps.2600630638 4852587

[B215] Peumans W. J. Zhang W. Barre A. Houlès Astoul C. Balint-Kurti P. J. Rovira P. (2000). Fruit-specific lectins from banana and plantain. Planta 211 (4), 546–554. 10.1007/s004250000307 11030554

[B216] Pongtuluran O. B. Rofaani E. (2015). Antiviral and immunostimulant activities of Andrographis paniculata. HAYATI J. Biosci. 22 (2), 67–72.

[B217] Poth A. G. Colgrave M. L. Lyons R. E. Daly N. L. Craik D. J. (2011). Discovery of an unusual biosynthetic origin for circular proteins in legumes. Proc. Natl. Acad. Sci. 108 (25), 10127–10132. 10.1073/pnas.1103660108 21593408 PMC3121837

[B218] PoussetQi J. L. W. Yue S.-J. S. (2006). Place des médicaments traditionnels en Afrique. Med. Trop. 66, 606–609. 17286033

[B219] Priyadarsini R. V. Manikandan P. Kumar G. H. Nagini S. (2009). The neem limonoids azadirachtin and nimbolide inhibit hamster cheek pouch carcinogenesis by modulating xenobiotic-metabolizing enzymes, DNA damage, antioxidants, invasion and angiogenesis. Free Radic. Res. 43 (5), 492–504. 10.1080/10715760902870637 19391054

[B220] Projean D. Baune B. Farinotti R. Flinois J.-P. Beaune P. Taburet A.-M. (2003b). *In Vitro* metabolism of chloroquine: identification of Cyp2C8, Cyp3a4, and Cyp2D6 as the main isoforms catalyzing N-desethylchloroquine formation. Drug Metab. Dispos 31, 748–754. 10.1124/dmd.31.6.748 12756207

[B221] Projean D. Morin P.-E. Tu T. M. Ducharme J. (2003a). Identification of CYP3A4 and CYP2C8 as the major cytochrome P450 s responsible for morphine N -demethylation in human liver microsomes. Xenobiotica 33, 841–854. 10.1080/0049825031000121608 12936704

[B222] Purvis B. Mao Y. Robinson D. (2019). Three pillars of sustainability: in search of conceptual origins. Sustain. Sci. 14 (3), 681–695. 10.1007/s11625-018-0627-5

[B223] Qian-Cutrone J. Huang S. Trimble J. Li H. Lin P.-F. Alam M. (1996). Niruriside, a new HIV REV/RRE binding inhibitor fromPhyllanthus niruri. J. Nat. Prod. 59, 196–199. 10.1021/np9600560 8991954

[B224] Rahman N. Basharat Z. Yousuf M. Castaldo G. Rastrelli L. Khan H. (2020). Virtual screening of natural products against type II transmembrane serine protease (TMPRSS2), the priming agent of coronavirus 2 (SARS-COV-2). Molecules 25 (10), 2271. 10.3390/molecules25102271 PMC7287752 32408547

[B225] Rahmasari R. Haruyama T. Charyasriwong S. Nishida T. Kobayashi N. (2017). Antiviral activity of Aspalathus linearis against human influenza virus. Nat. Product. Commun. 12 (4), 1934578X1701200. 10.1177/1934578x1701200432 30520604

[B226] Randhawa M. A. (2008). Black seed, Nigella sativa, deserves more attention. J. Ayub Med. Coll. Abbottabad 20 (2), 1–2. 19385445

[B227] Rao A. S. (1990). Root flavonoids. Bot. Rev. 56 (1), 1–84. 10.1007/bf02858531

[B228] Raza A. Muhammad F. Bashir S. Anwar M. I. Awais M. M. Akhtar M. (2015). Antiviral and immune boosting activities of different medicinal plants against Newcastle disease virus in poultry. World's Poult. Sci. J. 71 (3), 523–532. 10.1017/s0043933915002147

[B229] Rex J. R. S. Muthukumar N. M. S. A. Selvakumar P. M. (2018). Phytochemicals as a potential source for antimicrobial, anti-oxidant and wound healing-A review. MOJ Bioorg. Org Chem 2 (2), 61–70.

[B230] Riebensahm C. Ka D. Sow A. Semmo N. Wandeler G. (2019). A closer look at the spectrum of drug-induced liver injury in sub-Saharan Africa. Expert Rev. Clin. Pharmacol. 12 (9), 875–883. 10.1080/17512433.2019.1638251 31269818

[B231] Rivera J. O. Loya A. M. C. R. (2013). Use of herbal medicines and implications for conventional drug therapy medical sciences. Altern. Integ Med.

[B232] Rowaiye A. B. Olubiyi J. Bur D. Uzochukwu I. C. Akpa A. Esimone C. O. (2020). Silico screening and molecular dynamic simulation studies of potential small molecule immunomodulators of the Kir2Ds2 receptor. BioRxiv. 10.1101/2020.05.10.087148

[B233] Rüngeler P. Lyss G. Castro V. Mora G. Pahl H. Merfort I. (1998). Study of three sesquiterpene lactones fromTithonia diversifoliaon their anti-inflammatory activity using the transcription factor NF-κB and enzymes of the arachidonic acid pathway as targets. Planta Med. 64, 588–593. 10.1055/s-2006-957527 9810261

[B234] Saether O. Craik D. J. Campbell I. D. Sletten K. Juul J. Norman D. G. (1995). Elucidation of the primary and three-dimensional structure of the uterotonic polypeptide kalata B1. Biochemistry 34 (13), 4147–4158. 10.1021/bi00013a002 7703226

[B235] Salem M. L. (2005). Immunomodulatory and therapeutic properties of the Nigella sativa L. seed. Int. Immunopharmacol 5 (13–14), 1749–1770. 10.1016/j.intimp.2005.06.008 16275613

[B236] Salim B. (2020). Identification of compounds from nigella sativa as new potential inhibitors of 2019 novel coronavirus ( covid-19 ): molecular docking study. 19: 1–12.

[B237] Salinas F. M. Vázquez L. Gentilini M. V. O´Donohoe A. Regueira E. Nabaes Jodar M. S. (2019). Aesculus hippocastanum L. seed extract shows virucidal and antiviral activities against respiratory syncytial virus (RSV) and reduces lung inflammation *in vivo* . Antiviral Res. 164, 1–11. 10.1016/j.antiviral.2019.01.018 30711418

[B238] Sankaranarayanan R. Sekar K. Banerjee R. Sharma V. Surolia A. Vijayan M. (1996). A novel mode of carbohydrate recognition in jacalin, a Moraceae plant lectin with a β-prism fold. Nat. Struct. Mol. Biol. 3 (7), 596–603. 10.1038/nsb0796-596 8673603

[B239] Sarkar L. Putchala R. K. Safiriyu A. A. Das Sarma J. (2020). Azadirachta indica A. Juss ameliorates mouse hepatitis virus-induced neuroinflammatory demyelination by modulating cell-to-cell fusion in an experimental animal model of multiple sclerosis. Front. Cel. Neurosci. 14, 14. 10.3389/fncel.2020.00116 PMC7236902 32477069

[B240] Sciences G. Gilead sciences statement on positive data emerging from national institute of allergy and infectious diseases’ study of investigational antiviral remdesivir for COVID-19. Available at: https://WwwGileadCom/News-and-Press/Press-Room/Press-Releases/2020/4/Gilead-Sciences-Statement-on-Positive-Data-Emerging-From-National-Institute-of-Allergy-and-Infectious-Diseases-Study-of-Investigational-Antiviral-Rem. 2020.

[B241] Segun P. A. Ogbole O. O. Akinleye T. E. Faleye T. O. C. Adeniji A. J. (2019). *In vitro* anti-enteroviral activity of stilbenoids isolated from the leaves of Macaranga barteri. Nat. Product. Res., 1–5. 10.1080/14786419.2019.1644505 31343270

[B242] Shakibaei M. Mobasheri A. Buhrmann C. Curcumin synergizes with resveratrol to stimulate the MAPK signaling pathway in human articular chondrocytes *in vitro* . Genes Nutr. 2011:6:171–179. 10.1007/s12263-010-0179-5 21484156 PMC3092909

[B243] Sharon N. O. I. (1986). “Mannose specific bacterial surface lectins,” in Microbial lectins and agglutinins. Editor Mirelman D. (New York, NY: John Wiley Sons, Inc.), 55–82.

[B244] Shikalepo R. Mukakalisa C. Kandawa-Schulz M. Chingwaru W. Kapewangolo P. (2018). *In vitro* anti-HIV and antioxidant potential of *Bulbine* frutescens (Asphodelaceae). J. Herbal Med. 12, 73–78. 10.1016/j.hermed.2017.09.007

[B245] Shin H.-B. Choi M.-S. Ryu B. Lee N.-R. Kim H.-I. Choi H.-E. (2013). Antiviral activity of carnosic acid against respiratory syncytial virus. Virol. J. 10, 303. 10.1186/1743-422x-10-303 24103432 PMC3852111

[B246] Shivaprasad H. N. Kharya M. D. Rana A. C. Mohan S. (2006). Preliminary immunomodulatory activities of the aqueous extract ofTerminalia chebula. Pharm. Biol. 44 (1), 32–34. 10.1080/13880200500530542

[B247] Sindambiwe J. B. Calomme M. Cos P. Totté J. Pieters L. Vlietinck A. (1999). Screening of seven selected Rwandan medicinal plants for antimicrobial and antiviral activities. J. Ethnopharmacology 65 (1), 71–77. 10.1016/S0378-8741(98)00154-8 10350370

[B248] Sithisarn P. Supabphol R. Gritsanapan W. (2005). Antioxidant activity of Siamese neem tree (VP1209). J. Ethnopharmacology 99 (1), 109–112. 10.1016/j.jep.2005.02.008 15848028

[B249] Sodagari H. R. Aryan Z. Abdolghaffari A. H. Rezaei N. Sahebkar A. (2018). Immunomodulatory and anti-inflammatory phytochemicals for the treatment of inflammatory bowel disease (IBD): - turning strong rationale into strong evidence?. J. Pharmacopuncture 21 (4), 294–295. 10.3831/KPI.2018.21.033 30671279 PMC6333189

[B250] Sousa A. M. P. Salles H. O. Oliveira H. D. d. Souza B. B. P. d. Cardozo Filho J. d. L. Sifuentes D. N. (2020). Mo-HLPs: new flocculating agents identified from Moringa oleifera seeds belong to the hevein-like peptide family. J. Proteomics 217, 103692. 10.1016/j.jprot.2020.103692 32068186

[B251] Spaldin V. Madden S. Pool W. Woolf T. Park B. (1994). The effect of enzyme inhibition on the metabolism and activation of tacrine by human liver microsomes. Br. J. Clin. Pharmacol. 38, 15–22. 10.1111/j.1365-2125.1994.tb04316.x 7946932 PMC1364832

[B252] Sreejayan N. Rao M. N. (1996). Free radical scavenging activity of curcuminoids. Arzneimittelforschung 46, 169–171. 8720307

[B253] Sriwilaijaroen N. Fukumoto S. Kumagai K. Hiramatsu H. Odagiri T. Tashiro M. (2012). Antiviral effects of Psidium guajava Linn. (guava) tea on the growth of clinical isolated H1N1 viruses: its role in viral hemagglutination and neuraminidase inhibition. Antiviral Res. 94 (2), 139–146. 10.1016/j.antiviral.2012.02.013 22453134

[B254] Steinmassl M. Anderer F. A. (1996). Enhancement of human NK and LAK cytotoxicity against HCMV-infected cells by rhamnogalacturonan: specificity of reaction. Viral Immunol. 9, 27–34. 10.1089/vim.1996.9.27 8733917

[B255] Su X. Sangster M. Y. D'Souza D. H. (2010). *In vitro* effects of pomegranate juice and pomegranate polyphenols on foodborne viral surrogates. Foodborne Pathog. Dis. 7, 1473–1479. 10.1089/fpd.2010.0583 20807113

[B256] Sulaiman L. K. Oladele O. A. Shittu I. A. Emikpe B. O. Oladokun A. T. Meseko C. A. (2011). In-ovo evaluation of the antiviral activity of methanolic root-bark extract of the African Baobab (*Adansonia* digitata Lin). Afr. J Biotechnol 10 (20), 4256–4258.

[B257] Sundararajan A. Ganapathy R. Huan L. Dunlap J. R. Webby R. J. Kotwal G. J. (2010). Influenza virus variation in susceptibility to inactivation by pomegranate polyphenols is determined by envelope glycoproteins. Antiviral Res. 88, 1–9. 10.1016/j.antiviral.2010.06.014 20637243 PMC7114265

[B357] Surya W. Li Y. Torres J. (2018). Structural model of the SARS coronavirus E channel in LMPG micelles. Biochimica et Biophysica Acta (BBA)-Biomembranes 1860 (6), 1309–1317. 29474890 10.1016/j.bbamem.2018.02.017 PMC7094280

[B258] Swanson M. D. Winter H. C. Goldstein I. J. Markovitz D. M. (2010). A lectin isolated from bananas is a potent inhibitor of HIV replication. J. Biol. Chem. 285, 8646–8655. 10.1074/jbc.m109.034926 20080975 PMC2838287

[B259] Terstappen G. C. Reggiani A. (2001). In silico research in drug discovery. Trends Pharmacol. Sci. 22, 23. 11165668 10.1016/s0165-6147(00)01584-4

[B260] Thomford N. Awortwe C. Dzobo K. Adu F. Chopera D. Wonkam A. (2016). Inhibition of CYP2B6 by medicinal plant extracts: implication for use of efavirenz and nevirapine-based highly active anti-retroviral therapy (HAART) in resource-limited settings. Molecules 21, 211–226. 10.3390/molecules21020211 PMC6273559 26891286

[B261] Titanji V. P. Zofou D. Ngemenya M. N. (2008). The antimalarial potential of medicinal plants used for the treatment of malaria in Cameroonian folk medicine. Afr. J. Tradit Complement. Altern. Med. 5 (3), 302. 20161952 PMC2816552

[B262] Tomlinson E. S. Maggs J. L. Park B. K. Back D. J. (1997). Dexamethasone metabolism *in vitro*: species differences. J. Steroid Biochem. Mol. Biol. 62 (4), 345–352. 10.1016/s0960-0760(97)00038-1 9408089

[B263] Trujillo-Correa A. I. Quintero-Gil D. C. Diaz-Castillo F. Quiñones W. Robledo S. M. Martinez-Gutierrez M. (2019). *In vitro* and in silico anti-dengue activity of compounds obtained from Psidium guajava through bioprospecting. BMC Complement. Altern. Med. 19 (1), 298. 10.1186/s12906-019-2695-1 31694638 PMC6836419

[B264] Ubani A. Agwom F. Morenikeji O. R. Shehu N. Y. Luka P. Umera E. A. (2020). Molecular docking analysis of some phytochemicals on two SARS-CoV-2 targets. BioRxiv.

[B265] Udeinya J. I. Shu E. N. Quakyi I. Ajayi F. O. (2008). An antimalarial neem leaf extract has both schizonticidal and gametocytocidal activities. Am. J. Ther. 15 (2), 108–110. 10.1097/mjt.0b013e31804c6d1d 18356629

[B266] ul Qamar M. T. Alqahtani S. M. Alamri M. A. Chen L. L. (2020). Structural basis of SARS-CoV-2 3CLpro and anti-COVID-19 drug discovery from medicinal plants. J. Pharm. Anal. 10.1016/j.jpha.2020.03.009 PMC7156227 32296570

[B267] UNEP-WCMC (2016). The state of biodiversity in Africa: a mid-term review of progress towards the aichi biodiversity targets. Cambridge, United Kingdom: UNEP-WCMC.

[B268] Vimalanathan S. Kang L. Amiguet V. T. Livesey J. Arnason J. T. Hudson J. (2005). Echinacea purpurea. aerial parts contain multiple antiviral compounds. Pharm. Biol. 43 (9), 740–745. 10.1080/13880200500406354

[B269] Vlietinck A. J. De Bruyne T. Vanden Berghe D. A. (1997). Plant substances as antiviral agents. Curr. Org. Chem. 1 (4), 307–344.

[B369] Walls A. C. Park Y. J. Tortorici M. A. Wall A. McGuire A. T. Veesler D. (2020). Structure, function, and antigenicity of the SARS-CoV-2 spike glycoprotein. Cell 181 (2), 281–292. 32155444 10.1016/j.cell.2020.02.058 PMC7102599

[B270] Wang C.-K. Shih L.-Y. Chang K. (2017). Large-scale analysis of antimicrobial activities in relation to amphipathicity and charge reveals novel characterization of antimicrobial peptides. Molecules 22 (11), 2037. 10.3390/molecules22112037 PMC6150348 29165350

[B271] Weber F. (2020). Antiviral innate immunity: introduction. Ref Modul Life Sci.

[B272] Welch C. R. (2010). Chemistry and pharmacology of kinkéliba (Combretum micranthum), a West African medicinal plant. Doctoral Dissertation. New Brunswick (NJ): Rutgers Univ Sch Brunswick).

[B273] WHO International Union for Conservation of Nature and Natural Resources World Wide Fund for Nature. 1993. Guidelines on the conservation of medicinal plants. Gland, Switzerland: International Union for Conservation of Nature and Natural. Available at: https://apps.who.int/iris/handle/10665/41651.

[B274] WHO (2020). WHO supports scientifically-proven traditional medicine | WHO | Regional Office for Africa. WHO Support Sci Tradit Med Available at: https://www.afro.who.int/news/who-supports-scientifically-proven-traditional-medicine?gclid=CjwKCAjwjLD4BRAiEiwAg5NBFlOWbdSg5OgzIsNBICCwbaCndOvz_Nk8onOJzRLqZw9YhMVHMhRsbxoC9_wQAvD_BwE.

[B275] Wiart C. Kumar K. Yusof M. Y. Hamimah H. Fauzi Z. M. Sulaiman M. (2005). Antiviral properties of ent-labdene diterpenes ofAndrographis paniculata nees, inhibitors of herpes simplex virus type 1. Phytother. Res. 19 (12), 1069–1070. 10.1002/ptr.1765 16372376

[B276] Wolf R. Tufano M. A. Ruocco V. Grimaldi E. Ruocco E. Donnarumma G. (2006). Quinine sulfate inhibits invasion of some bacterial skin pathogens. Int. J. Dermatol. 45 (6), 661–663. 10.1111/j.1365-4632.2006.02696.x 16796622

[B277] Woo E.-R. Piao M. S. (2004). Antioxidative constituents fromlycopus lucidus. Arch. Pharm. Res. 27 (2), 173–176. 10.1007/bf02980102 15022718

[B278] World Health Organization (2020). Naming the coronavirus disease (COVID-19) and the virus that causes it. Geneva, Switzerland: World Health Organization.

[B279] Worthen D. R. Ghosheh O. A. Crooks P. A. (1998). The *in vitro* anti-tumor activity of some crude and purified components of blackseed, Nigella sativa L. Anticancer Res. 18 (3A), 1527–1532. 9673365

[B280] Wu J. Luo X. Guo H. Xiao J. Tian Y. (2006). Transgenic cotton, expressing *Amaranthus* caudatus agglutinin, confers enhanced resistance to aphids. Plant Breed. 125 (4), 390–394. 10.1111/j.1439-0523.2006.01247.x

[B281] Wu S.-F. Lin C.-K. Chuang Y.-S. Chang F.-R. Tseng C.-K. Wu Y.-C. (2012). Anti-hepatitis C virus activity of 3-hydroxy caruilignan C from Swietenia macrophylla stems. J. Viral Hepat. 19, 364–370. 10.1111/j.1365-2893.2011.01558.x 22497816

[B282] Xin Y. Xiangrong Z. Mingju Z. Wenchao G. Yingchuan T. Qizhong X. (2011). Transgenic potato overexpressing the *Amaranthus* caudatus agglutinin gene to confer aphid resistance. Crop Sci. 51 (5), 2119–2124. 10.2135/cropsci2010.11.0650

[B283] Xu J. Wang J. Deng F. Hu Z. Wang H. (2008). Green tea extract and its major component epigallocatechin gallate inhibits hepatitis B virus *in vitro* . Antiviral Res. 78, 242–249. 10.1016/j.antiviral.2007.11.011 18313149

[B284] Yang Y. Xiu J. Zhang L. Qin C. Liu J. (2012). Antiviral activity of punicalagin toward human enterovirus 71 *in vitro* and *in vivo* . Phytomedicine 20 (1), 67–70. 10.1016/j.phymed.2012.08.012 23146421

[B285] Yeh R. F. Gaver V. E. Patterson K. B. Rezk N. L. Baxter-Meheux F. Blake M. J. (2006). Lopinavir/ritonavir induces the hepatic activity of cytochrome P450 enzymes CYP2C9, CYP2C19, and CYP1A2 but inhibits the hepatic and intestinal activity of CYP3A as measured by a phenotyping drug cocktail in healthy volunteers. J. Acquir Immune Defic Syndr. 42 (1), 52–60. 10.1097/01.qai.0000219774.20174.64 16639344

[B286] Yin Q.-h. Yan F.-x. Zu X.-Y. Wu Y.-h. Wu X.-p. Liao M.-c. (2012). Anti-proliferative and pro-apoptotic effect of carvacrol on human hepatocellular carcinoma cell line HepG-2. Cytotechnology 64 (1), 43–51. 10.1007/s10616-011-9389-y 21938469 PMC3261448

[B287] Younas M. Hano C. Giglioli-Guivarc'h N. Abbasi B. H. (2018). Mechanistic evaluation of phytochemicals in breast cancer remedy: current understanding and future perspectives. RSC Adv. 8 (52), 29714–29744. 10.1039/c8ra04879g PMC9085387 35547279

[B288] Zakaryan H. Arabyan E. Oo A. Zandi K. (2017). Flavonoids: promising natural compounds against viral infections. Arch. Virol. 162 (9), 2539–2551. 10.1007/s00705-017-3417-y 28547385 PMC7087220

[B289] Zhang J. Tang Q. Zimmerman-Kordmann M. Reutter W. Fan H. (2002). Activation of B lymphocytes by GLIS, a bioactive proteoglycan from Ganoderma lucidum. Life Sci. 71 (6), 623–638. 10.1016/S0024-3205(02)01690-9 12072151

[B290] Zhang W. Lim L.-Y. (2008). Effects of spice constituents on P-Glycoprotein-Mediated transport and CYP3A4-mediated metabolism *in Vitro* . Drug Metab. Dispos 36, 1283–1290. 10.1124/dmd.107.019737 18385293

[B291] Zhang Y. Liu Y.-B. Li Y. Ma S.-G. Li L. Qu J. (2013). Sesquiterpenes and alkaloids from the roots of Alangium chinense. J. Nat. Prod. 76, 1058–1063. 10.1021/np4000747 23734721

[B292] Zhang Z. Leong D. J. Xu L. He Z. Wang A. Navati M. Friedman J. M. (2016). Curcumin slows osteoarthritis progression and relieves osteoarthritis-associated pain symptoms in a post-traumatic osteoarthritis mouse model. Arthritis Res. Ther. 18 (1), 1–12. 10.1186/s13075-016-1025-y 27260322 PMC4891896

[B293] Zhou S. Gao Y. Jiang W. Huang M. Xu A. Paxton J. W. (2003). Interactions of herbs with cytochrome P450. Drug Metab. Rev. 35 (1), 35–98. 10.1081/dmr-120018248 12635815

[B294] Zibaee E. Kamalian S. Tajvar M. Amiri M. S. Ramezani M. Moghadam A. T. Emami S. A. (2020). Citrus species: a review of traditional uses, phytochemistry and pharmacology. Cpd 26 (1), 44–97. 10.2174/1381612825666191127115601 31775593

