# Corrigendum: Therapeutic Potentials of Antiviral Plants Used in Traditional African Medicine With COVID-19 in Focus: A Nigerian Perspective

**DOI:** 10.3389/fphar.2021.721060

**Published:** 2021-07-20

**Authors:** Alfred Francis Attah, Adeshola Adebayo Fagbemi, Olujide Olubiyi, Hannah Dada-Adegbola, Akinseinde Oluwadotun, Anthony Elujoba, Chinedum Peace Babalola

**Affiliations:** ^1^ Department of Pharmacognosy and Drug Development, Faculty of Pharmaceutical Sciences, University of Ilorin, Ilorin, Nigeria; ^2^ Department of Pharmaceutical Chemistry, Faculty of Pharmacy, University of Ibadan, Ibadan, Nigeria; ^3^ Department of Pharmaceutical Chemistry, Obafemi Awolowo University, Ile-Ife, Nigeria; ^4^ Institute of Biological Information Processing, Structural Biochemistry (IBI-7), Forschungszentrum Jülich, Jülich, Germany; ^5^ Department of Medical Microbiology and Parasitology, College of Medicine, University of Ibadan, Ibadan, Nigeria; ^6^ Nestle Nigeria Plc, Lagos, Nigeria; ^7^ Department of Pharmacognosy, Faculty of Pharmacy, Obafemi Awolowo University, Ile-Ife, Nigeria; ^8^ Centre for Drug Discovery, Development and Production, University of Ibadan, Ibadan, Nigeria; ^9^ College of Basic Medical Sciences, Chrisland University, Abeokuta, Nigeria

**Keywords:** COVID-19, phytomedicines, Traditional African Medicine, herbal immuno-stimulants, herb-drug interaction

In the original article, there was a mistake in Supplementary Table S1 as published. This table is totally missing; instead, Figure 7 (chemical structures 13–172) has been replaced here. Meanwhile, this same Figure 7 has incompletely been presented within the original article where only structures 13–23 appeared within the article without a link or statement directing readers to the remaining structures. The corrected Supplementary Table S1 appears below.

**FIGURE 7:**
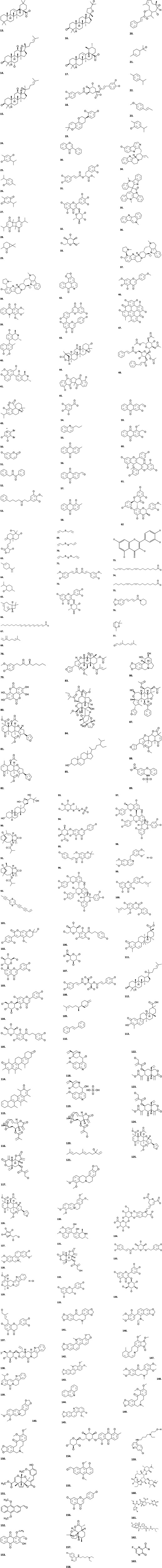
Structures of some plant-derived secondary metabolites with antiviral activities.

The authors apologize for this error and state that this does not change the scientific conclusions of the article in any way. The original article has been updated.

