# Supplementary material for: Therapeutic Potentials of Antiviral Plants Used in Traditional African Medicine With COVID-19 in Focus: A Nigerian Perspective
Source: Front Pharmacol. 2021 Apr 26;12:596855. doi: 10.3389/fphar.2021.596855 (PMC8108136; doi:10.3389/fphar.2021.596855)
Supplement: Supplementary file 1 [file Table1.pdf]

Supplementary table S1. Antimalarial plants used in Traditional African Medicine (TAM): Documented antioxidant assays, immunomodulatory, anti-Inflammatory activities, level of evidence (LEV I-V*) and knowledge gaps

African plants≠/country	Antimalarial activity (*in vitro/vivo*)	Antioxidant assay[a]	Immunomodulatory activity	Anti-inflammatory activity	LEV I-V*/ Reference
Acacia nilotica (L.) Delile Fabaceae<br><br>South Sudan, Nigeria	*In vitro*	Leaves, pods and bark extracts all showed antioxidant activity with the leaves extracts possessing the highest activity	Aqueous extracts of leaves showed immunomodulatory activity	Diterpenes isolated from bark of Acacia nilotical produced in vitro anti-inflammatory activity	[c,e,f]**LEV III**/ (Eldeen et al., 2010; Sadiq et al., 2017)
Acacia seyal Delile Fabaceae<br><br>West Africa	*In vivo*	The methanol fraction produced interesting DPPH free radical scavenging activity	NR	The methanolic crude extracts showed good anti-inflammatory activities at a dose of 300 mg/kg	[c]**LEV II**/ (Elnour et al., 2018; Nguta and Mbaria, 2013)
Acanthospermum hispidum DC. Compositae<br><br>Nigeria	*In vitro*	Phenolic rich fractions possessed strong antioxidant activity by inhibiting hydroxyl radical, hydrogen peroxide and nitric oxide scavenging.	*A. hispidum* enhances the proliferation of T lymphocytes in the porcine immune system.	NR	[e,f]**LEV IV & V*** / (Gomathi et al., 2013; Koukouikila-Koussounda et al., 2013; Summerfield and Saalmüller, 1998)
Adansonia digitata L. Malvaceae<br><br>Namibia, Nigeria, Africa	*In vivo*	Extracts produced antioxidant activity and inhibited α -glucosidase.	Aqueous and Methanol extracts modulate immune response	Fruit pulp produced anti-inflammatory activity	[c,e,]**LEV II, IV** **(**Braca et al., 2018; Diallo et al., 2002; Musila et al., 2013; Ramadan et al., 1994; Sharma and Rangari, 2016**)**

Allium sativum L. Amaryllidaceae<br><br>Nigeria	*In vivo*	All test forms displayed in vitro antioxidant activity with fried garlic being the highest.	*A. sativum* maintained the homeostasis of the immune system to produce beneficial effects on the immune cells, mainly via the regulation of proliferation and cytokine gene expression.	Allicin from *A. sativum* inhibited Th1 proinflammatory cytokines<br><br>Allicins produced an inhibitory effect on NF-κB activation.	[e,f]**LEV IV & V*** /(Bruck et al., 2005; Dorhoi et al., 2006; Hodge et al., 2002; Moutia et al., 2018; Queiroz et al., 2009; Ruslan and Baba, 2018)
Aloe viridiflora Reynolds Asphodelaceae<br>South Africa, Namibia	*In vitro*	In vitro antioxidant activity	Modulate immune response	Chronic anti-inflammatory activity	[c,e,f]**LEV II, IV, V*/ (**Cock 2015; Patel and Patel, 2013; Salehi et al., 2018; Van Zyl et al., 2002**)**
Alstonia boonei De Wild. Apocynaceae<br><br>Nigeria	*In vivo*	It displayed a good DPPH (2,2-diphenyl-1-picrylhydrazyl) radical scavenging activity (41.58 ± 1.43 %).	NR	The alcoholic extract demonstrated significant protection of the paw against the induced inflammation.	[c,f]**LEV II & V*** / (Akinmoladun et al., 2007; Imam et al., 2017; Osadebe, 2002)
Anacardium occidentale L. Anacardiaceae<br><br>Nigeria, West Africa	*In vitro*	Extract displayed an interesting in vitro antioxidant activity.	NR	Unripe cashew apple juice exhibited a good anti-inflammatory activity by a significant inhibition of ear edema.	[c,e,f]**LEV II, IV & V***/ (Gimenez et al., 2019; Kamath and Rajini, 2007; da Silveira Vasconcelos et al., 2015)
Ananas comosus (L.) Merr. Bromeliaceae	*In vivo*	The aqueous extract displayed a good DPPH activity while the	In vitro and in vivo experiment demonstrated that bromelain limit the	Extract controlled secretion of tumour necrosis factor-α,	[c]**LEV II**/ (Kargutkar and Brijesh 2018;

Nigeria		methanol extract showed a good ABT activity	severity of angina pectoris, transient ischemic attacks and prevented aggregation of human blood platelets and decreased the symptoms in hypertensive patients.	interleukin-1β and prostaglandins, Carrageenan-induced acute paw edema was still markedly decreased by it.	Priya, 2014; Putri et al., 2018; Uzor et al., 2020)
Annickia chlorantha (Oliv.) Setten & Maas<br>Annonaceae<br><br>Nigeria, Liberia	*In vivo*	Free radical scavenging potentials of the extracts were found to be proportional to their respective phenolic and flavonoid contents	Aqueous extract exerted a transient immune modulation in rat	Methanol extracts produced anti-inflammatory effect in mice	[c]**LEV II**/ (Adebajo et al., 2013; Adesokan and Akanji 2010; Olanlokun and Akomolafe 2013; Olivier et al., 2015; Otimenyin and Uguru 2006)
Annona senegalensis Pers.<br>Annonaceae<br><br>Nigeria, Mali	*In vivo; In vitro*	Extract and fractions exhibited good in vitro antioxidant activities at higher concentrations	Extract may stimulate immune response	Root bark extract displayed anti-inflammatory potential	[c,e,]**LEV I**I, IV/ (Adzu et al., 2005; Ajaiyeoba et al., 2006; Diallo et al., 2002; Ngbolua et al., 2014; Omeke et al., 2019)
Anthonotha macrophylla P. Beauv<br>Fabaceae<br><br>West Africa	*In vitro*	NR	NR	NR	[e,f]**LEV IV & V***/ (Zirihi et al., 2005)
Azadirachta indica A.Juss.<br>Meliaceae	*In vivo*	NR	The extract displayed marked increase in	Neem oil displayed significant anti-	[c]**LEV II** / (Jagadeesh and

Nigeria			phagocytic index. The extract also showed increase in antibody titer against the ovalbumin and protection towards the cyclophosphamide induced myelosuppression in dose dependent manner.	inflammatory effect in both acute as well as chronic inflammation	Srinivas, 2014; Oseni and Akwetey, 2012)
Bauhinia thonningii Schum. Fabaceae<br><br>Nigeria, Mali	*In vitro*	Extracts displayed strong in vitro antioxidant activities.	Promotes healing and restores health	Isolated polyphenols showed antiinflammatroy potentials as also produced by aqueous and methanol extracts	[c,e,f]**LEV II**, IV, V*/ (Diallo et al., 2002; Ibewuike et al., 1997; Madara et al., 2010; Moriasi et al., 2020; Olela et al., 2020)
Bersama abyssinica Fresen Melianthaceae<br><br>Ethiopia, West Africa	*In vitro*	In vitro antioxidant activity observed in the water fraction	NR	The antiinflammatory activities<br>of the leaves supports the bioactive compounds in present	[e,f]**LEV IV & V***/ (Kifle and Enyew, 2020; Lather et al., 2010; Zekeya et al., 2014; Zirihi et al., 2005)
Bridelia ferruginea Benth. Phyllanthaceae<br><br>Nigeria	*In vivo*	The aqueous extract of stem bark inhibited the formation of TBARS induced by the pro-oxidant, sodium nitroprusside, it reverses the effect of lipid peroxidation in the liver and brain tissue of albino-Wistar rats both	Albino rats fed with aqueous extract of stem bark significant decreases in the level of hemoglobin (Hb), packed cell volume and percent monocyte counts while significant increases were observed in percent	Stem bark extract showed inhibition of lipopolysaccaride-induced septic shock and vascular permeability	[c,e]**LEV II, IV**/ (Kolawole and Adesoye 2010; Mbah et al., 2012; Olajide et al., 2003; Olarewaju et al., 2013; Oloyede and Babalola,

		at a concentration of 0.33 mg/mL, with IC_{50} values of 3.00 ± 1.58 mg/mL and 2.99 ± 1.59 mg/mL for the liver and brain homogenates respectively.	neutrophil and lymphocyte counts.		2012; Shittu et al., 2020)
Cajanus cajan (L.) Millsp.<br><br>Fabaceae<br><br>Nigeria, West Africa	*In vitro*	In the beta-carotene-linoleic acid test, extracts produced a comparable inhibition capacity to the positive control.<br>The butanol fraction displayed DPPH radical scavenging potential	Hexane extract induced a decrease in TNF-α and IL-6, as well as significant decrease in IgG serum levels	The hexane extract (200 and 400 mg/kg) retarded carrageenan-induced inflammation by 85 and 95%, respectively.	[c,e]**LEV II, IV**/ (Ajaiyeoba et al., 2013; Hassan et al., 2016; Wu et al., 2009)
Canna indica L.<br><br>Cannaceae<br><br>Nigeria, West Africa	*In vitro*	Rhizomes have been investigated as a good source of antioxidants, showing significant activity in food and biological model systems	Ethanol extract stimulates HG in U937 monocytes resulting in activation of p38 MAPK, ERK1/2, and JNK	Ethanol extract inhibits the production of inflammatory mediators including NO, IL1β, and PGE2 from LPS-induced RAW 264.7 macrophages	[d]**LEV III (**Al-Snafi, 2015b; Ayusman et al., 2020; Ménan et al., 2006**)**
Canthium glaucum Hiern<br>Rubiaceae<br><br>Nigeria	*In vivo*	NR	NR	NR	[c]**LEV I**I/ (Musila et al., 2013)
Capsicum frutescens L**.**<br><br>Solanaceae<br><br>Nigeria, West Africa	*In vivo*	DPPH assay, using *n*-hexane and chloroform extracts demonstrated 26.9% and 30.9% free radical scavenging abilities, respectively, at	Administration of capsicum extract (1 and 10 mug/mL) and capsaicin (3 and 30 muM) facilitated the suppression of interleukin (IL)-2, interferon (IFN)-gamma,	NR	[c,e,f]**LEV II, IV, V* (**Gurnani et al., 2016; Habte and Assefa, 2020; Takano et al., 2007**)**

		the concentration of 1 mg/mL.	IL-4 and IL-5 production in cultured murine Peyer's patch (PP) cells in vitro and ex vivo.		
Carica papaya L. Caricaceae<br><br>Nigeria	*In vitro*	NR	Leaf extract facilitate the upregulation of immunomodulatory genes.	The anti-inflammatory activity of an ethanolic extract of leaves produced significant reduction in the amount of granuloma formed	[d]**LEV III**/ (Bamidele et al., 2008; Imaga et al., 2010; Kovendan et al. 2012; Noriko et al., 2010)
Cassia occidentalis Leguminosae<br><br>West Africa	*In vitro*	Extracts produced a dose dependent regulation of oxidative stress markers. The extract produced a marked reduction in malondialdehyde (MDA) levels of murine hepatic microsomes at 100 microg/mL (56%).	An improved macrophage activity and more H_2O_2 production ($P<0.05$) in cells of birds given 0.75% *Cassia occidentalis*.	Extract reduced carrageenan-induced inflammation in mice at a dose of 250 mg/kg.<br><br>The ethyl acetate extract of roots showed a pronounced anti-inflammatory activity.	[c]**LEV II** / (Al-Snafi 2015a; Ntchapda et al., 2015; Patel et al., 2014; Sreejith et al., 2010; Zirihi et al., 2005)
Ceiba pentandra (L.) Gaertn. Malvaceae<br><br>Nigeria	*In vitro*	The extracts of *C. pentandra* revealed relatively high levels of total phenolics and flavonoids. Extracts demonstrated dose dependent reducing power activity.	NR	Seed oil exhibited anti-inflammatory activity by showing percentage of membrane stability that compared favourably with that of standard Diclofenac in a concentration depended manner.	[c,e]**LEV II, IV**/ (Jasso-Miranda et al., 2019; Loganayaki et al., 2013; Rao 2014)

Chromolaena odorata (L.) R.M.King & H.Rob.<br>Compositae<br>Nigeria	*In vivo*		Soluble polysaccharides (PoS) fraction showed immunostimulatory activity via stimulation of PBMC and production of IFN-γ in a dose-dependent manner.	Aqueous extract displayed anti-inflammatory activity and inhibited formaldehyde induced arthritis.	[c,d]**LEV II, III**/ (Akinmoladun et al., 2007; Ezenyi et al., 2014; Thaddée et al., 2015; Owoyele, 2005)
Chrysophyllum albidum G.Don Sapotaceae<br>Nigeria	*In vitro*	Myricetin rhamnoside (an extracted compound from the ethyl ether fraction) exhibited an excellent radical scavenging activity	ethanol extract of peel significantly inhibits tumor necrosis factor-alpha, interleukin-6 levels and reduced immunopositive expression of COX-2 and NF-κB.	ethanol extract of peel suppressed inflammatory responses in carrageenan-induced air pouch	[c,d]**LEV II, III**/ (Adebayo et al., 2011; Adedapo, 2020; Mb et al., 2018)
Citrus aurantiifolia (Christm.) Swingle<br>Rutaceae<br>Nigeria	*In vivo*	The oil displayed an interesting in-vitro antioxidant activity.	Extract inhibited the proliferation of phytohemagglutinin (PHA) activated mononuclear cells at 250 and 500 µg/mL.	NR	[c,d]**LEV II, III**/ (Al-Aamri et al., 2018; Ettebong et al., 2019; Gharagozloo, 2001)
Citrus aurantium L.<br>Rutaceae<br>Nigeria	*In vitro*	The peel, flowers and leaf oils of all exhibited antioxidant activity with the essential oils in the old leaves having the most antioxidant activity.	The essential oil inhibited the production of interleukin-6 (IL-6) (98.11 ± 1.62%), tumor necrosis factor-α (TNF-α) (41.84 ± 1.52%), and interleukin-1β (IL-1β) (56.09 ± 2.21%) as well	The essential oil markedly decreased the expression levels of cyclooxygenase-2 (COX-2) gene and protein.	[d]**LEV III**/ (Chun-Yan et al., 2017; Sanei-Dehkordi et al., 2016; Sarrou et al., 2013)

			as their gene expression level.		
Citrus paradisi Macfad.<br>Rutaceae<br>Nigeria	*In vitro*	The glyceric extract demonstrated a good in vitro antioxidant activity.	Peels possessed immunostimulation activity via augmentation of proliferation of mouse splenocytes (Tlymphocytes).	In acute colitis, *C. paradisi*, was found to be efficacious for the management of inflammatory bowel disease.	[c,d]**LEV II, III**/ (Diab 2016; Giamperi et al., 2004; Ivoke et al., 2013; Rafeeq, 2016)
Coccinia barteri Hook. F.<br>Cucurbitaceae<br>Nigeria	*In vivo*	Extract produced in vitro antioxidant activity	NR	NR	[c]**LEV II**/ (Hamid et al., 2017; Orabueze et al., 2020)
Cochlospermum planchonii Hook.f. ex Planch. Bixaceae<br>Ivory Coast, West Africa	*In vitro; in vivo*	Broad spectrum of in vitro antioxidant activity	NR	Extract caused a biphasic inhibition of carrageenan-induced paw edema	[c,e,f]**LEV I**I, **IV, V***[/] **(**(Anaga and Oparah, 2009; Dakuyo et al., 2015; Oumar et al., 2014; Yerbanga et al., 2012**)**
Cryptolepis sanguinolenta (Lindl.) Schlechter<br>Apocynaceae<br>Tanzania, Angola, West Africa	*In vitro; in vivo*	Extracts produced inhibition of xanthine oxidase and scavenged superoxide anions	Polysaccharides were assessed to strongly inhibit MSP1 (Malaria antigen)-induced overproduction of IL-1β, IL-6 and TNF-α in in vitro immunological assays.	Cryptolepine alkaloid inhibited lipopolysaccharide (LPS)-induced microvascular permeability in mice in a dose-related fashion.	[c,d]**LEV II, III**/ (Cimanga et al., 1997; Cimanga et al., 2000; Francine et al., 2018; Olajide et al., 2003; Wright et al., 1996)
Curcuma longa L.<br>Zingiberaceae<br>Nigeria	*In vivo*	The essential oil and ethanol oleoresin of fresh and dry rhizomes	Extracts showed a significant increase of NO, IL-2, IL-6, IL-10, IL-12, interferon (IFN)	An aqueous based extractand fractions showed potent inhibitory effect towards	[c,d,f]**LEV II, III**,**V*** / (Chinampudur et al., 2013;

		of *C. longa* Linn. have antioxidant properties.	gamma, tumor necrosis factor (TNF) alpha and MCP-1 production in unstimulated mouse splenocytes and mouse macrophages.	release of PGE_2 and IL-12 levels in LPS stimulated mouse splenocytes.	Lwin et al., 2017; Singh et al., 2010)
Cymbopogon citratus (DC.) Stapf<br>Poaceae<br><br>Nigeria	*In vivo*	Essential oil demonstrated free radical scavenging activity in peripheral blood mononuclear cells at all test concentrations.	*C. citratus* inhibited IL-6 release and LPS action after macrophages incubation with LPS.	Leaves infusion and its flavonoid-rich and tannin-rich fractions in the acute inflammation model displayed percentage oedema inhibition	[c,d]**LEV II, III** / (Chukwuocha, et al., 2016; Jamuna et al., 2017; Sforcin et al., 2015)
Dichrostachys cinerea (L) Wight et Arn<br>Fabaceae<br><br>Cape Verde, Somalia, Namibia	*In vivo*	Aqueous fraction showed potent antioxidant activity by ferric ions reducing power, DPPH assay and ferrous ions chelating activity	Extracts produced immunomodulatory activity on human peripheral blood mononuclear cells	Stem bark, leaf, and root were effective in acute model as revealed by anti-inflammatory screening	[c]**LEV II**/ (Hurinanthan 2009; Nguta and Mbaria, 2013; Susithra and Jayakumari, 2018)
Diospyros mespiliformis Hochst. ex A.DC.<br><br>Ebenaceae<br><br>Nigeria	*In vivo*	NR	NR	The methanol extract of *D. mespiliformis* (50 and 100 mg/kg i.p.) gave a significant activity ($P<0.05$) against all the anti-inflammatory models used.	[c,f]**LEV II, V***/ (Adzu and Salawu, 2009; Adzu et al., 2002)
Enantia chlorantha Oliv.[g]<br><br>Annonaceae<br><br>Nigeria	*In vivo*	Extract showed a free radical scavenging activity.	NR	NR	[c,f]**LEV II, V***/ (Agbaje and Onabanjo, 1991; Olanlokun and

					Akomolafe, 2013)
Erigeron floribundus Asteraceae<br><br>West Africa	*In vitro*	*E. floribundus* essential oil showed a significant ferric reducing antioxidant power (tocopherol-equivalent antioxidant capacity	Aqueous leaf<br><br>extract of *E. floribundus* has a stimulating effect on the blood levels of neutrophil cells, total lymphocytes and TCD4+ cells. Extract inhibited the immune-deficiency induced by methylprednisolone.	The extract markedly reduced the rat paw oedema volume at 50 mg/kg and above.	[c]**LEV IV** / (Asongalem et al., 2004; Petrelli et al., 2016; Yapo et al., 2011; Zirihi et al., 2005)
Eucalyptus globulus Labill Myrtaceae<br><br>Nigeria, Southern Africa	*In vitro*	The essential oils of *E. globulus* produced high levels of monoterpenes (eucalyptol) with significant in vitro antioxidant activity.	*E. globulus* oil dose-dependently stimulated phagocytosis and immune modifying effects	Essential oils produced neutrophil-dependent and independent anti-inflammatory activities	[d,e,f]**LEV III, IV, V***/ (Méndez et al., 2019; Sadlon and Lamson, 2010; Zofou et al., 2011)
Euphorbia hirta Euphorbiaceae<br><br>West Africa	*In vitro*	Methanol extract of *E. hirta* produced an interesting DPPH.<br><br>Ethanol extract exhibited good superoxide scavenging activity	leaf extract stimulated specific immune response in fish while higher concentrations stimulated the production of antibodies only up to the 5th day.	*E. hirta*-treated mice had a significant reduction in the levels of pro-inflammatory cytokines, down regulated cell activation markers and co-stimulatory molecules, and up regulated anti-inflammatory cytokines.	[c]**LEV II** / (Ahmad et al., 2014; Asha et al., 2016; Chen et al., 2015; Ismail et al., 2019; Pratheepa and Sukumaran, 2014; Zirihi et al., 2005)
Ficus capensis Thunb. Moraceae	*In vitro*	Leaf extracts (essential oil,	Aqueous extract of leaf in rats demonstrated a dose-dependent increase in	The extract inhibited ACE (IC_{50} = 52.17), AChE (IC_{50} = 172.60	[c]**LEV II** / (Akomolafe et al., 2016;

West Africa		methanol-water and water) produced DPPH-informed antioxidant activity; Extract rich in phenolic compounds scavenged NO and OH radicals, chelated Fe^{2+} and inhibited Fe^{2+} lipid peroxidation	leukocyte mobilization, with doses 150 and 250 mg/kg giving total leukocyte count of 4.44±0.39×10 9 and 6.10±0.86×10 9 /L, respectively.	µg/mL) and arginase (IC_{50} = 112.50 µg/mL) activities in a dose-dependent manner.	Daikwo and Tende, 2012; Muanda et al., 2010; Zirihi et al., 2005)
Flueggea virosa (Roxb. ex Willd.) Royle<br><br>Phyllanthaceae<br><br>Zimbabwe, Mali	*In vitro*	Extracts produced good in vitro antioxidant activity	NR	Isolated trinorditerpenes possess antiinflammatroy potentials	[e,f]**LEV** IV, V*/ (Chao et al., 2014; Chauke et al., 2012; Kaou et al., 2008)
Gossypium barbadense L.<br><br>Malvaceae<br><br>Nigeria	*In vivo*	Extract showed free radical scavenging activity and reducing power.	NR	Aqueous leaf extract significantly modulated and improved the pH, mucin content, glutathione (reduced) as well as gastric activities of superoxide dismutase and catalase in ulcerated rats.	[c,d,f]**LEV II, V***/ (Ade-Ademilua and Okpoma, 2018; Sabiu et al., 2017; Salako and Awodele, 2012)
Gossypium hirsutum Cav.[g]<br><br>Malvaceae<br><br>Nigeria	*In vivo*	The antioxidant effect of gossypol from Gossypium spp has been documented	NR	NR	[c,f]**LEV II, V***/ (Ade-Ademilua and Okpoma, 2018; Al-Snafi, 2018)
Guiera senegalensis J.F. Gmel.<br>Combretaceae<br><br>Africa	*In vitro*	Tannins and terpenes from *G. senegalensis*	Aqueous extract of *G. senegalensis* appear to elicit immunomodulatory properties.	Crude extracts produced antiinflammatroy activity	[c,e,f]**LEV II, IV, V***/ (Benoit et al., 1996; Bouchet et al.,

		exhibited radical scavenging activity			1998; Jigam et al., 2011; Parvez et al., 2018; Sahabi and Abubakar 2018)
Gymnosporia senegalensis (Lam.) Loes.<br>Celastraceae<br>Tanzania, Mali	*In vitro; in vivo*	Extract and its fractions produced in vitro<br>antioxidant activities	NR	The dichloromethane fractions showed inhibition of TNF-α synthesis by cells.<br>Extract and its fractions markedly reduced inflammatory cells and improved glutathione level in lung homogenate	[c,e,f]**LEV II, IV, V***/ (Gessler et al., 1994; Gessler et al., 1995; Kpoyizoun et al., 2020; Makgatho et al., 2018)
Haematostaphis barteri Hook.f.<br>Anacardiaceae<br>South Sudan, West Africa	*In vivo*	The aqueous extract produced antioxidant activity via reducing power and nitric oxide assays.	NR	Hihg doses showed anti-inflammatory activity in animal models	[c,f]**LEV II, V***/ (Boampong et al., 2015)
Harungana madagascariensis Lam. ex Poir.<br>Hypericaceae<br>Nigeria	*In vitro*	The metal chelating activity was higher in the methanolic extract which also showed greater ferric reducing power and was richer in phenolics (132.24±0.61 mgGAE/g) and flavonoids.	NR	The leaf inhibited formaldehyde-induced arthritis in vivo	[c,e,f]**LEV II, IV, V*** / (Antia et al., 2015; Medewase and Ezike, 2018; Ndjakou et al., 2007)
Heliotropium indicum L.<br>Boraginaceae<br>Nigeria	*In vitro*	The ethanolic extract showed marked reducing power and free radical scavenging. It	The dried leaves extract (200mg/mL) markedly raised the in vitro phagocytic index and	Ethanol extract inhibited both the clinical scores of inflammation and inflammatory cells	[c,d,f]**LEV II, III**/(Ashoka et al., 2009; Kyei et al., 2016;

		was also concentration dependant.	lymphocyte viability in all assays. They also made a marked increase in antibody titer, carbon clearance and delayed type hypersensitivity in mice.	infiltration. Also, the protein level and the concentrations of TNF-α, PGE2 and MCP-1 in the aqueous humor were also markedly reduced.	Santhosha et al., 2015)
Irvingia gabonensis (Aubry-Lecomte ex O'Rorke) Baill. Irvingiaceae<br><br>Nigeria, West Africa	*In vitro*	Petroleum ether extract had the best total antioxidant capacity at 500 µg/mL as the activity was not markedly different from ascorbic acid. Lupeol showed an increased antioxidant activity across the concentrations when compared with standards (ascorbic acid) in a DPPH and FRAP analytical assessments.	NR	The noxious effect of sodium arsenite (SA)-induced hepatic pro-inflammatory cytokines and haematological derangements in Wistar rats was ameliorated by post-treatment and concomitant treatment with ethanol leaf extract.	[c,f]**LEV II & V*** / (Abdurahman and Abdulhakim, 2020; Ekpe et al., 2019; Ewere et al., 2020; Zirihi, et al., 2005)
Jatropha curcas Linn. Euphorbiaceae<br><br>Nigeria, West Africa	*In vitro; in vivo*	It exhibited a strong antioxidant activity.	Leaves have potent immunomodulatory activity on humoral and cell-mediated immune response in chicks	Anti-inflammatory activity of *Jatropha curcas* roots in mice and rats has been reported	[c,e]**LEV II**, IV (Abd-Alla et al., 2009; Abiodun, et al., 2011; Airaodion and Ogbuagu, 2020; Mujumdar and Misar 2004; Rofida, 2015)
Jatropha gossypifolia L. Euphorbiaceae<br><br>Nigeria, West Africa	*In vitro*	In vitro DPPH radical scavenging activity of oils obtained from the	1-phenylnaphthalene lignans content of this plant has been reported to show promise as	A significant in vitro anti inflammatory activity was documented	[d,e]**LEV III, IV**/ (Akshada et al., 2008; Deo et al., 2012; Gbeassor

		leaves and stem have been reported	immunomodulatory agents	for various solvent extracts	et al., 1989; Nagaharika and Rasheed, 2013; Okoh et al., 2016; Onyegbule et al., 2019)
Khaya grandifoliola A. Juss. Meliaceae Nigeria	*In vitro*	The methylenechloride methanol and water extracts inhibited microsomal lipid peroxidation.	Polysaccharide fractions isolated from stem bark strongly inhibited MSP1 (Malaria antigen)-induced overproduction of IL-1β, IL-6 and TNF-α by PBMCs in the in vitro immunological assays.	The methanol extract of stem bark significantly inhibited the carrageenan-induced paw oedema	[c,e,f]**LEV II, III, V*** (Abiodun and CHING, 2009; Bickii et al., 2000; Francine et al., 2018; Njayou et al., 2013)[0]
Lippia multiflora Moldenke Tropical Africa	*In vitro*	Phenylethanoid glycosides of *L. multiflora* identified as major antioxidants	NR	Essential oils obtained from the plant showed anti-inflammatory effects	[c,d]**LEV II, III**/ (Abena et al., 2003; Arthur et al., 2011; Benoit et al., 1996)
Mangifera indica L. Anacardiaceae Nigeria	*In vivo*	It expressed a potent scavenging activity on hydroxyl radicals and hypochlorous acid while still acting as an iron chelator. The extract also showed a marked inhibitory effect on the peroxidation of rat-brain phospholipid and inhibited DNA damage by copper-phenanthroline system.	Ethanol extract produced an increase in humoral antibody (HA) titre and delayed type hypersensitivity (DTH) in mice.	Extract reduced myeloperoxidase (MPO) activity. The extract also inhibited tumor necrosis factor alpha (TNFalpha) serum levels.	[c,f]**LEV II, III/** (Gabino et al., 2004; Malann et al., 2014; Martínez et al., 2000; Tanveer et al., 2005; Wright et al., 1996; Zirihi et al., 2005)

Mareya micrantha (Benth). Müll. Arg.[g]<br><br>Euphorbiaceae<br><br>West Africa	*In vitro*	NR	NR	NR	(Zirihi et al., 2005)
Microglossa pyrifolia (Lam.) Kuntze[g]<br><br>Compositae<br><br>West Africa	*In vitro*	5,7,4 - trihydroxy-3,8,3-trimethoxyflavone isolated from *M. pyrifolia* produced antioxidant activity	NR	NR	(Akimanya et al., 2015; Zirihi et al., 2005)
Milicia excelsa (Welw.) C.C.Berg<br>Moraceae<br><br>Nigeria	*In vivo*	Extract showed a good free radical scavenging potential.	NR	The crude extract and ethyl acetate fraction showed anti-inflammatory potentials.	[c,e,f]**LEV II, IV, V* (**Akinpelu et al., 2019; Areola et al., 2016; Ayepola et al., 2018**)**
Millettia zechiana Harms[g]<br><br>Leguminosae<br><br>West Africa	*In vitro*	NR	NR	NR	(Zirihi et al.,, 2005)
Momordica foetida Schumach.<br>Cucurbitaceae<br>Tropical Africa, Namibia, Swaziland, South Africa	*In vitro; in vivo*	The aqueous extract has shown good antioxidant activity	NR	NR	[c,e,f]**LEV I**I, IV/ (Waako et al., 2005)
Mondia whitei<br><br>(Hook.f.) Skeels<br>Apocynaceae	*In vivo*	Extract showed in vitro antioxidant activity	Hexane fraction of *Mondia whitei* had a reversible androgenic effect and potentiates the	NR	[c,f]**LEV II, V***/ (Fred-Jaiyesimi and Ogunjobi 2013; Owolabi et al., 2019;

Nigeria			action of norepinephrine on rat vas deferens.		Watcho et al., 2005)
Morinda lucida Benth.<br>Rubiaceae<br><br>Nigeria	*In vivo*	The leaf and root oils exhibited different antioxidant abilities which were concentration dependent.	Hydroethanolic leaf extract reduced pro-inflammatory cytokines interleukin (IL)-1β and tumor necrotic factor, but elevated levels of anti-inflammatory cytokine IL-10 *in vitro*.	Hydroethanolic leaf extract revealed profound activity against localized and systemic inflammation in inverse dose-dependent manner and caused reduction in nitric oxide and prostaglandin E2	[c,d]**LEV II, III**/ (Frederick et al., 2020; Okoh et al., 2011; Owolabi et al., 2019)
Morinda morindoides (Baker) Milne-Redh.<br>Rubiaceae<br><br>Nigeria	*In vitro*	Ethanol and dichloromethane extracts reduced DPPH, inhibited lipid peroxidation and markedly minimised and normalized the activity of SOD, CAT and the concentration of NO	NR	Extracts showed anti-inflammatory effects	[c,e,f]**LEV II, IV, V***/ (Kipré et al., 2015; Lucien et al., 2015; Mohammed et al., 2020)
Moringa oleifera Lam.<br>Moringaceae<br><br>Nigeria, West Africa	*In vivo*	The leaf extracts exhibited interesting DPPH radical scavenging and FRAP total reducing power activities	Extracts produced immunomodulatory effects on cyclophosphamide induced toxicity in mice.	The leaf and seed extracts (11.1–100 g/mL) exhibited significant anti-inflammatory activities via inhibition of NO production.	[c,f]**LEV II, V***/ (Gupta et al., 2010; Mulisa et al., 2018; Xu et al., 2019)
Nauclea latifolia Blanco<br><br>Rubiaceae	*In vitro*	The whole fruit produced a remarkable in vitro antioxidant activity.	NR	Ethanoland aqueous extracts of leaves demonstrated anti-inflammatory activities	[d,f]**LEV III, V*/** (Benoit-Vical et al., 1998; Franklyn et al.,

Nigeria					2020; Oyedeji-Amusa and Ashafa 2019)
Nauclea pobeguinii (Pobég. ex Pellegr.) Merr. ex E.M.A. Petit<br>Rubiaceae<br><br>Sub-Saharan Africa	*In vitro; in vivo*	Produced a significant reduction in the production of intra- and extracellular reactive oxygen species	Extracts decreased T cell proliferation	Extracts alleviated joint inflammation and pain sensitivity	[b,c,e]**LEV I, II, IV**/**(** Lusakibanza et al., 2010; Mesia et al., 2011; Mesia et al., 2012**)**
Ocimum gratissimum Forssk.<br>Lamiaceae<br><br>Nigeria	*In vivo*	Methanol extract showed a DPPH scavenging activity and reductive potential comparable to gallic acid and ascorbic acid respectively.	Aqueous extract increased the decreased adherence, chemotaxis, phagocytosis, and intracellular killing of bacteria in nicotine-treated macrophages. It protects the murine peritoneal macrophages	The aqueous leaves extract exhibited anti-inflammatory effect which was significant ($P<0.001$) at all the doses tested.	[c,d]**LEV II, III** / (Akinmoladun, et al., 2007; Kefe et al. 2016; Santanu et al., 2011; Tanko et al., 2008)
Periploca nigrescens Afzel<br>Apocynaceae<br><br>Nigeria, West Africa	*In vitro*	Extracts, at 50 mg/mL produced marked ($P < 0.05$) inhibitory effects on Fe2+/ascorbate-induced<br>lipid peroxidation in rat liver mitochondria.<br><br>There were marked increases in	NR	*Extract* produced a marked ($p<0.05$) inhibition of various types of inflammation (carrageenan oedema, cotton pellet granuloma, and formaldehyde arthritis).	[c,f]**LEV II & V*** / (Akinrinmade et al., 2016; Ayoola et al., 2011; Owoyele et al., 2009; Zirihi et al., 2005)

		malondialdehyde and significant reductions in reduced glutathione and glutathione S-transferase activity with IR injury, while pre-treatment with methanol extract prevented these effects.			
Persea americana Mill<br>Lauraceae<br><br>Nigeria, West Africa	*In vivo; In vitro*	Seed extracts found to be high in total phenolics resulting in good antioxidant activity	Lipid-Rich seed extract regulates innate immune response in bovine mammary epithelial cells	The extract exhibited a dose-dependent inhibition of carrageenan-induced rat paw oedema.	[c,d]**LEV II, III**/ (Adeyemi et al., 2002; Báez-Magaña et al., 2019; Komlaga et al., 2015; Kosińska et al., 2012; Onyishi, 2020)
Philenoptera cyanescens (Schum. & Thonn.) Roberty[g]<br><br>Fabaceae<br><br>Cameroon, West Africa	NR<br>Ecxept in Nigerian antimalarial ethnomedicine	NR	NR	NR	(Chinedu et al., 2014)
Phyllanthus amarus Schumach. & Thonn.<br>Phyllanthaceae<br><br>Nigeria, West Africa)	*In vivo*	Higher total polyphenol, flavonoids and flavonol were seen in the methanol fraction of the extract and higher radical cation scavenging (TEAC) activity was observed in the aqueous extract.	*Extract* attenuated elevated oxido-nitrosative stress (Nrf2 and iNOs), immune-inflammatory makers (HO-1, TNF-α, IL-1β, and TGF-β1), Th2 cytokines (IL-4 and IL-6) levels.	*Extract* exerted various anti-inflammatory activities via perturbation of the NF-κB, MAPKs, PI3K/Akt, and Wnt signaling networks.	[c,d,f]**LEV II, III, V*** / (Harikrishnan, et al., 2020; Ojezele et al., 2017; Olabiyi et al., 2020; Wu et al., 2019)
Physalis angulate L.<br>Solanaceae	*In vivo*	Isolated withanolide modulates LPS binding	Extracts possessed stimulatory activity on B	The anti-inflammatory activity of the extract	[c,f]**LEV II, V*/** (Lusakibanza, et

Nigeria		to TLR4 receptor and up-regulates the antioxidant activity	cells and less effect on T cells and augmented the antibody response in BALB/c and C3H/HeJ mice.	against carrageenan-induced paw edema was dose-dependent	al., 2010; Ukwubile et al., 1992)
Picralima nitida (Stapf) T. Durand & H. Durand Apocynaceae<br><br>Nigeria, Africa	*In vivo*	Methanol extract showed superior in vitro antioxidant activity	Butanol extract elicited the most potent immunosuppressive effects	Pseudo-akuammigine alkaloid from the seeds of *P. nitida* possess anti-inflammatory activity	[c]**LEV II**/ (Biruksew et al., 2018; Duwiejua et al., 2002; Stoilova et al., 2007; Teugwa et al., 2013)
Plectranthus barbatus Andrews Lamiaceae<br><br>Kenya	*In vivo*	Strong in vitro antioxidant activity	Extract enhanced the stimulation of protective immune responses resulting in anti-HIV-1 activity	Extracts reduced the production of pro-inflammatory cytokines	[c,d]**LEV II, III**/ (Kapewangolo and Meyer, 2018; Kapewangolo, et al.,2013; Kiraithe et al., 2016)
Psidium guajava L. Myrtaceae<br><br>Nigeria	*In vivo*	The leaves extracts of different guava cultivars showed more scavenging effects on free radicals than the commercial guava tea extracts and dried fruit extract	Fish (8.33 ± 1.02 g) that were injected with guava leaf extracts (10 or 100 µg/fish) intraperitoneally demonstrated increased activity in lymphocyte proliferation, nitric oxide production, and respiratory burst until the 14th day and declined afterward.	Strongly inhibited lipopolysaccharide (LPS)-induced production of nitric oxide and prostaglandin E2 in a dose-dependent manner. Extracts demonstrated anti-inflammatory activity in 2 different animal models	[c,e,f]**LEV II**/ (Chen and Yen, 2007; Malacrida and Jorge, 2013; Master and Abraham, 2017; Mi et al., 2014)

Pycnanthus angolensis (Welw.) Warb.<br>Myristicaceae<br><br>West Africa	*In vitro*	Against DPPH, purified sargachromenol and sargahydroquinoic acid was enough evidence that the compounds are good free radical scavengers.	Myristeoleic acid have been characterized from the seeds; cetyl myristoleate (CMO), a derivative of myristeoleic acid, is effective in the treatment of joint inflammatory disease	Crude seed extract and isolated compounds (sargachromenol, sargahydroquinoic acid) inhibited LPS-induced COX-2 mRNA expression	[c,f]**LEV II & V*** / (Leonard, 2010; Simon et al., 2008; Zirihi et al., 2005)
Rauvolfia vomitoria Afzel**.**<br>Apocynaceae<br><br>(Nigeria)	*In vivo*	Alkaloids and ethanol aqueous extracts of *R. vomitoria* displayed a high antioxidant activity.	NR	Alkaloid rauvomine C isolated from the stems showed significant anti-inflammatory activities on NO production in LPS-induced RAW264.7 mouse macrophages with IC_{50} value of 10.76 μM.	[c,d]**LEV II, III**/ (Erasto et al., 2011; Guanqun et al., 2020; Momoh et al., 2014)
Sambucus nigra L.<br>Adoxaceae<br><br>North Africa	*In vitro*	Anthocyanin flavonoids are constituents of *S. nigra* known for antioxidant properties	Extract caused an increase in the cytokine production using monocytes from 12 healthy human donors. There was a dose-dependent increase in secretion of proinflammatory cytokines	Extracts produced the inhibition of proinflammatory activities of major periodontal pathogens	[b,d]**LEV I, III**/ (Barak and Halperin, 2001; Benoit et al., 1996; Peter and Michael, 1998)
Sclerocarya birrea (A.Rich.) Hochst.<br>Anacardiace*ae*<br><br>Madagascar, Nigeria, Africa	*In vitro; In vivo*	The extracts from varied parts showed antioxidant activities.	NR	The aqueous and methanol stem-bark extracts (500 mg/kg p.o.) reduced rat paw oedema. However, the methanol extract of the plant produced a greater anti-	[c,e,f]**LEV II, IV, V***/ (Armentano et al., 2015; Mariod and Abdelwahab, 2012; Ojewole et al., 2010)

				inflammatory effect than its aqueous extract	
Securidaca longepedunculata Fresen.<br><br>Polygalaceae<br><br>Nigeria, Ghana, Kenya, South Africa	*In vivo*	Extract exhibited strong in vitro antioxidant activity	Enhanced non-specific immune function *in vitro*	Extract exhibited strong anti inflammatory activity	[c,e,f]**LEV II**, III, V*/ (Muanda et al., 2010; Nguta, 2019; Ottendorfer et al., 1994)
Senna podocarpa (Guill. & Perr.) Lock[g]<br><br>Fabaceae<br><br>Nigeria	NR.<br>Only antimalarial ethnomedicinal uses reported	The high level of flavonoids, tannins and phenolic content in the leaf of *S. podocarpa* correlates with the concentration-dependent DPPH radical scavenging activity of the aqueous extract	NR	NR	[f]**LEV V*** (Adebesin et al., 2013)
Senna siamea (Lam.) H.S.Irwin & Barneby<br><br>Fabaceae<br><br>Nigeria	*In vitro*	In vivo antioxidant activity of alcoholic extract at a dose of 50–150 mg/kg of body weight exhibited antioxidant activity	NR	Various solvent extracts of *S. siamea* showed significant and dose-dependent anti-inflammatory effects	[c,e]**LEV II, IV**/(Kamagaté et al., 2014; Kaur et al., 2006; Ntandou et al., 2010); [304]
Solanum nigrum L.<br><br>Solanaceae<br><br>Nigeria	*In vitro*	In vitro antioxidant activity of *S. nigrum* correlated well with the total phenolic content	Polysaccharides extracted from *S. nigrum* displayed immunomodulatory effects via the TLR4-MyD88 signaling pathway	Berries of *S. nigrum* are high in steroidal glycosides with anti-inflammatory activity	[d]**LEV III**/ (Haddad et al., 2017; Loganayaki et al., 2010; Pu et al., 2020; Xiang et al., 2018)
Sorghum bicolour (L.) Moench. Poaceae<br><br>Nigeria, West Africa	*In vivo*	Phenol contents of correlated highly with the antioxidant activity	Ethanol extracts of seed and leaf sheath modulates the immune functions in macrophages	African *Sorghum bicolor* leaf sheaths showed anti-inflammatory potentials	[c]**LEV II**/ (Awika et al., 2003; Benson et

		using ORAC, ABTS and DPPH			al., 2013; Cho et al., 2016)
Sphenocentrum jollyanum Pierre<br>Menispermaceae<br><br>Nigeria	*In vivo*	Leaf extract displayed in vivo antioxidant activity in mice	NR	Methanol extracts and isolated furanoditerpene showed anti-inflammatory effect	[c]**LEV II**/ (Moody et al., 2006; Olorunnisola and Afolayan, 2011, 2013)
Strychnos spinosa Lam.<br>Loganiaceae<br><br>West Africa	*In vitro*	Extract showed dose dependent antioxidants activity with the highest radical inhibition of 43.47±2.50 % at 100 μg/mL compared to 90.12±1.61 % of the standard (Ascorbic acid).	NR	The water, chloroform and n-butanol fractions displayed lipoxygenase activity	[e,f]**LEV IV & V*** / (Ndarubu et al., 2020; Sadau and Eloff, 2014; Zirihi et al., 2005)
Tamarindus indica (L.)<br>Fabaceae<br><br>Nigeria	*In vivo*<br>.	Polyphenolic compounds extracted from defatted seed showed antioxidant activity using $O_2^{\bullet-}$, $OH^{\bullet}$,$DPPH^{\bullet}$, $ABTS^{\bullet+}$ and FRAP	Polysaccharide-rich seed extract produced immunopotentiating activity in mice	Leaf and seed elicited anti-inflammatory activities in an in vivo model	[c]**LEV II**/ (Aravind et al., 2012; Bhadoriya et al., 2012; Nguta and Mbaria, 2013; Siddhuraju, 2007; Suralkar et al., 2012)
Terminalia catappa Linn.<br>Combretaceae<br><br>Nigeria, West Africa	*In vitro*	Extracts exhibited good in vitro antioxidant properties	Immunomodulatory activity has been reported which could be attributed to the phenolic compounds	Antiinflammatory activities linked to Phenolic compounds identified	[c,d]**LEV II**, III/ (Abiodun et al., 2011; Abiodun et al.,, 2016; Chyau et al., 2006)
Theobroma cacao L.<br>Malvaceae	*In vitro*	The content of flavonoids such as	The effect of cocoa flavonoids on	Cocoa exerts regulatory activity on the secretion	[c,d]**LEV II, III**/ (Komlaga et al.,

Nigeria, Ivory-Coast, West Africa		epicatechin, catechin and procyanidins in cocoa is linked with the potent antioxidant acitivitiy	adaptive immunity has been reported and *in vivo* studies support the immunomodulating effect of cocoa	of inflammatory mediators from macrophages and other leucocytes *in vitro*.	2015; Ramiro-Puig and Castell, 2009)
Tithonia diversifolia (Hemsl.) A.Gray<br>Asteraceae<br><br>Nigeria	*In vitro*	Potent in vitro antioxidant activity of the plant extracts tested	20–100 mg/kg of the saponin extract of *T. diversifolia* enhanced the immune function	Methanol extract of the leaves at 50–200 mg/kg produced dose-related inhibition of carrageenan-induced paw oedema	[c,e]**LEV II, IV**/ (da Gama et al., 2014; Ejelonu et al., 2017; Goffin et al., 2002)
Trema orientalis (L.) Blume<br>Cannabaceae<br><br>Nigeria	*In vitro; in vivo*	The methanol extract showed strong in vitro antioxidant activity while aqueous extract showed a weaker activity	NR	Methanol leaf extract produced a potent anti-inflammatory activity in vivo	[c,e]**LEV II, IV**/ (Olanlokun et al., 2017; Oyebola et al., 2008)
Tridax procumbens (L.) L.<br>Compositae<br><br>Nigeria, West Africa	*In vitro*	The extracts produced antioxidant activity against DPPH and ABTS free radicals.	Aqueous extracts elicited immunomodulatory effect in experimental animals	The standardized ethylacetate, methanol and 70% ethanol extracts of the shoot exhibited marked inhibition of rat paw edema at a medium dose of 200 mg/kg	[c,e]**LEV II, IV**/ (Jachak et al., 2011; Komlaga et al., 2015; Tiwari et al., 2004)
Vangueria infausta Burch.<br>Rubiaceae<br><br><br>South and East Africa	*In vitro; in vivo*	Quercetin-3-O-glucoside and quercetin isolated from methanol extract of leaves revealed free radical (DPPH), H_2O_2 scavenging activities and reducing power potential as compared to rutin.	NR	crude ethanol extract and flavonoid fraction exhibited potent anti-inflammatory activity.	[c,e,f]**LEV II**, **IV, V***[/]**(** Abosi et al.,2006; Nundkumar and Ojewole, 2002**)**

Vernonia amygdalina Delile<br>Asteraceae<br>Nigeria, West Africa	*In vivo*	Extracts produced a dose-dependent decrease ($p<0.05$) in some oxidative stress indices including nitric oxide and lipid peroxidation levels	Extracts showed some immunomodulatory activity in mice	Ethanol extracts inhibited proinflammatory cytokines	[c]**LEV II**/ (Challand and Willcox, 2009; Kraft et al., 2003; Omoregie and Pal, 2016)
Vernonia bipontini Vatke.[s]<br>Compositae<br>Ethiopia, Eritrea	*In vivo*	NR	NR	NR	(Assefa et al., 2007)
Vitex doniana Sweet<br>Lamiaceae<br>Nigeria, West Africa	*In vitro*	Extracts produced in vitro antioxidant activity in a concentration-dependent manner.	Polysaccharides from the plant showed immunomodulatory properties	Leaves of the plant showed anti-inflammatory activity	[e,f]**LEV IV, V***/**(** Abiodun et al., 2011; Agbafor and Nwachukwu, 2011; Dénou et al., 2019; Iwueke et al., 2006**)**
Withania somnifera (L.) Dunal.<br>Solanaceae<br>Africa	*In vivo*	The root, stem and leaves extracts produced a significant scavenging effect on DPPH, chelating activity and reducing power with the scavenging effect of the extracts comparable to standard ascorbic acid.	*W. somnifera* root powder showed immunosuppressive effect following in vitro and in vivo investigations	*W.somnifera* showed a significant inhibition of TNFα in the adult zebrafish	[c,e]**LEV II, IV**/ (Dikasso et al., 2006; Rasool and Varalakshmi, 2006; Sivamani et al., 2014; Yadava et al., 2011)
Ximenia americana L.<br>Olacaceae<br>East Africa	*In vitro*	Methanol extracts and bioactive compounds of the fruit of *X. Americana*	Ethanol extracts produced in vitro	Aqueous ethanol extract of root bark of *X.americana* possesses	[c,e,f]**LEV II, IV, V***/(Almeida et al., 2016; Olabisi et al.,

		showed antioxidant activity<br><br>.	immunomodulating properties	anti- inflammatory properties	2011; Shettar et al., 2015)
Xylopia aethiopica (Dunal) A.Rich.<br>Annonaceae<br><br>Nigeria	*In vivo*	Treatment with 200, 400 and 600 mg/kg of aqueous extract effectively reduced ethanol induced raised activity of the malondialdehyde levels and increased the activity of total antioxidant capacity in the rats, but the effects was not dose dependent.	The extract showed activity towards 5-LOX, resulting in marked inhibition at concentrations ranging from 16 to 250 μg/mL (IC_{50} = 85 μg/mL).	Administration of X. aethiopica suppressed paw oedema at 100 and 300 mg kg^{-1} to 72.39±4.38% and 60.81±3.25% of the inflamed control response respectively.	[c,e,f]**LEV II, IV, V*/** (Boampong, et al., 2013; Chuks-Oguine et al., 2020; Macedo et al., 2020; Obiri and Osafo, 2013)
Zanthoxylum chalybeum Engl.<br>Rutaceae<br><br>Kenya, Tanzania	*In vivo*	Extracts displayed good in vitro antioxidant activity.	NR	Antiinflammatory activity via inhibition of cyclooxygenase activity	[c,e,f]**LEV II**, IV, V*/ (Kiraithe et al., 2016; Matu and Van Staden, 2003; Tufts et al., 2015)

[NR]Not reported; [≠] The taxonomic description of each plant may slightly differ from the reported one because it is based on new data that clarifies the plant taxonomy and documented on The Plant List (http://www.theplantlist.org/) as well as World Flora Online (http://www.worldfloraonline.org/).

[a]Since chemical anti-oxidant assays are of no pharmacological relevance, all FRAP, ABTS, DPPH and other in vitro chemical antioxidant assays documented here have been used to define the chemical profile of each of the plant presented. There is therefore no evidence of therapeutic benefits for such documented in vitro antioxidant assays.

[b]LEV I – Evidence from at least one clinical study.

[c]LEV II - Inferences supported by in vivo experiments.

[d]LEV III - Detailed mechanistic and other in vitro evaluations support the conclusion

[e]LEV IV - Evidence from preliminary in vitro screening

[f]LEV V* - Findings are based on limited or very poor quality evidence

[g]Very limited antimalarial evidence with a gap in knowledge in anti-inflammatory and immunomodulatory properties

REFERENCES

1. Sadiq, M. B., Tharaphan, P., Chotivanich, K., Tarning, J., & Anal AK. In vitro antioxidant and antimalarial activities of leaves, pods and bark extracts of Acacia nilotica (L.) Del. *BMC Complement Altern Med*. 2017:17(1), 372.

2. Eldeen, I. M. S., Van Heerden, F. R., & Van Staden J. In vitro biological activities of niloticane, a new bioactive cassane diterpene from the bark of Acacia nilotica subsp. kraussiana. *J Ethnopharmacol*. 2010:128(3), 555-560.

3. Nguta, J. M., & Mbaria JM. Brine shrimp toxicity and antimalarial activity of some plants traditionally used in treatment of malaria in Msambweni district of Kenya. *J Ethnopharmacol*. 2013:148(3), 988-992.

4. Elnour, A. A. M., Mirghani, M. E. S., Kabbashi, N. A., Md Alam, Z., & Musa KH. Study of antioxidant and anti-Inflammatory crude methanol extract and fractions of Acacia seyal Gum. *Am J Pharmacol Pharmacother*. 2018:5(1), 3.

5. Koukouikila-Koussounda, F., Abenab, A. A., Nzounganic, A., Mombouli, J. V., Ouambae, J. M., Kunf, J., & Ntoumia F. In vitro evaluation of antiplasmodial activity of extracts of Acanthospermum hispidum DC (Asteraceae) and Ficus thonningii blume (Moraceae), two plants used in traditional medicine in the Republic of Congo. *African J Tradit Complement Altern Med*. 2013:10(2), 270-276.

6. Gomathi, V., Palanisamy, P., & Jaykar B. Preliminary phytochemical and in-vitro antioxidant activity of the whole plant of Acanthospermum Hispidum DC. *Int J Med Pharm*. 2013:1, 22-32.

7. Summerfield, A., & Saalmüller A. Interleukin-2 dependent selective activation of porcine γδ T lymphocytes by an extract from the leaves of Acanthospermum hispidum. *Int J Immunopharmacol*. 1998:20(1-3), 85-98.

8. Musila, M. F., Dossaji, S. F., Nguta, J. M., Lukhoba, C. W., & Munyao JM. In vivo antimalarial activity, toxicity and phytochemical screening of selected antimalarial plants. *J Ethnopharmacol*. 2013:146(2), 557-561.

9. Braca, A., Sinisgalli, C., De Leo, M., Muscatello, B., Cioni, P. L., Milella, L., ... & Sanogo R. Phytochemical profile, antioxidant and antidiabetic activities of Adansonia digitata L.(Baobab) from Mali, as a source of health-promoting compounds. *Molecules*. 2018:23(12), 3104.

10. Diallo, D., Sogn, C., Samaké, F. B., Paulsen, B. S., Michaelsen, T. E., & Keita A. Wound healing plants in Mali, the Bamako region. An ethnobotanical survey and complement fixation of water extracts from selected plants. *Pharm Biol*. 2002:40(2), 117-128.

11. Sharma, A., & Rangari V. Immunomodulatory activity of methanol extract of Adansonia digitata L. *Trop J Pharm Res*. 2016:15(9), 1923-1927.

12. Ramadan, A., Harraz, F. M., & El-Mougy SA. Anti-inflammatory, analgesic and antipyretic effects of the fruit pulp of Adansonia digitata.

FITOTERAPIA-MILANO-,. 1994:65, 418-418.

13. Ruslan, M.S. & Baba MS. In vivo antimalarial assessment and toxicity evaluation of garlic (Allium sativum) in plasmodium berghei NK65-induced mice. *Malaysian Appl Biol*. 2018:47(5), 17-24.

14. Queiroz, Y. S., Ishimoto, E. Y., Bastos, D. H., Sampaio, G. R., & Torres EA. Garlic (Allium sativum L.) and ready-to-eat garlic products: in vitro antioxidant activity. *Food Chem*. 2009:115(1), 371-374.

15. Hodge, G., Hodge, S., & Han P. Allium sativum (garlic) suppresses leukocyte inflammatory cytokine production in vitro: potential therapeutic use in the treatment of inflammatory bowel disease. *Cytom J Int Soc Anal Cytol*. 2002:48(4), 209-215.

16. Moutia, M., Habti, N., & Badou A. In Vitro and In Vivo Immunomodulator Activities of Allium sativum L. *Evidence-Based Complement Altern Med*. 2018.

17. Dorhoi, A., Dobrean, V., Zăhan, M., & Virag P. Modulatory effects of several herbal extracts on avian peripheral blood cell immune responses. *Phyther Res An Int J Devoted to Pharmacol Toxicol Eval Nat Prod Deriv*. 2006:20(5), 352-358.

18. Bruck, R., Aeed, H., Brazovsky, E., Noor, T., & Hershkoviz R. Allicin, the active component of garlic, prevents immune-mediated, concanavalin A-induced hepatic injury in mice. *Liver Int*. 2005:25(3), 613-621.

19. Van Zyl, R. L., Viljoen, A. M., & Jäger AK. In vitro activity of Aloe extracts against Plasmodium falciparum. *South African J Bot*. 2002:68(1), 106-110.

20. Salehi, B., Albayrak, S., Antolak, H., Kręgiel, D., Pawlikowska, E., Sharifi-Rad, M., ... & Varoni EM. Aloe genus plants: from farm to food applications and phytopharmacotherapy. *Int J Mol Sci*. 2018:19(9), 2843.

21. Patel, K., & Patel DK. Medicinal importance, pharmacological activities, and analytical aspects of aloin: *A concise report J Acute Dis*. 2013:2(4), 262-269.

22. Cock IE. The genus aloe: phytochemistry and therapeutic uses including treatments for gastrointestinal conditions and chronic inflammation. In Novel natural products: therapeutic effects in pain, arthritis and gastro-intestinal diseases. *Springer, Basel*. 2015:(pp. 179-235).

23. Osadebe PO. Anti inflammatory properties of the root bark of Alstonia boonei. *Niger J Nat Prod Med*. 2002;(January):6(1):39-41.

24. Imam, A. A., Ezema, M. D., Muhammad, I. U., Atiku, M. K., Alhassan, A. J., Idi, A., ... & Mohammed A. In vivo Antimalarial Activity of Solvents Extracts of Alstonia boonei Stem Bark and Partial Characterization of Most Active Extract (s). *Annu Res Rev Biol*. 2017:1-11.

25. Akinmoladun, A. C., Ibukun, E. O., Afor, E., Akinrinlola, B. L., Onibon, T. R., Akinboboye, A. O., ... & Farombi EO. Chemical constituents and antioxidant activity of Alstonia boonei. *African J Biotechnol*. 2007:6(10).

26. Kamath, V., & Rajini PS. The efficacy of cashew nut (Anacardium occidentale L.) skin extract as a free radical scavenger. *Food Chem*. 2007:103(2), 428-433.

27. da Silveira Vasconcelos, M., Gomes-Rochette, N. F., de Oliveira, M. L. M., Nunes-Pinheiro, D. C. S., Tomé, A. R., Maia de Sousa, F. Y., ... & de Melo DF. Anti-inflammatory and wound healing potential of cashew apple juice (Anacardium occidentale L.) in mice. *Exp Biol Med*. 2015:240(12), 1648-1655.

28. Gimenez VMM, Alvarenga TA, Groppo M, et al. Antiplasmodial evaluation of Anacardium occidentale and alkyl-phenols. *Rev Bras Farmacogn*. 2019;29(1):36-39. doi:10.1016/j.bjp.2018.11.003

29. Priya Saxena DP. Cardioprotective potential of hydro-alcoholic fruit extract of Ananas comosus against isoproterenol induced myocardial infraction in Wistar Albino rats. *J Acute Dis*. 2014:3: 228-234.

30. Uzor, P. F., Ishiwu, B. U., & Nwodo NJ. In vivo antimalarial effect of Ananas comosus (L) Merr (Bromeliaceae) fruit peel, and gas chromatography-mass spectroscopy profiling: A possible role for polyunsaturated fatty acid. *Trop J Pharm Res*. 2020:19(1), 137-145.

31. Putri, D. A., Ulfi, A., Purnomo, A. S., & Fatmawati S. Antioxidant and antibacterial activities of Ananas comosus peel extracts. *Malaysian J Fundam Appl Sci*. 2018:14(2), 307-11.

32. Kargutkar, S., & Brijesh S. Anti-inflammatory evaluation and characterization of leaf extract of Ananas comosus. *Inflammopharmacology*. 2018:26(2), 469-477.

33. Adebajo, A. C., Aliyu, A. F., Odediran, S. A., Nwafor, P. A., Nwoko, T. N., Umana, S. U., & Adeoye AO. In vivo antiplasmodial activities of four Nigerian medicinal plants. *Planta Med*. 2013:79(13), PE3.

34. Olanlokun, J. O., & Akomolafe SF. Antioxidant potentials of various solvent extracts from stem bark of Enantia chlorantha. *J Biomed Sci Eng*. 2013:6, 877-884.

35. Otimenyin, S. O., & Uguru MO. Acute toxicity studies, anti-inflammatory and analgesic activities of the methanolic extract of the stem bark of Enantia chlorantha and Nauclea latifolia. *J Pharm Bioresour*. 2006:3(2), 111-115.

36. Adesokan, A. A., & Akanji MA. Haematological changes following chronic administration of aqueous extract of Enantia Chlorantha to albino rats. *Trop J Heal Sci*. 2010:17(1).

37. Olivier, D. K., Van Vuuren, S. F., & Moteetee AN. Annickia affinis and A. chlorantha (Enantia chlorantha)–a review of two closely related medicinal plants from tropical Africa. *J Ethnopharmacol*. 2015:176, 438-462.

38. Omeke, C. P., Udodeme, H. O., Nwafor, F. I., & Ezugwu CO. Antioxidant and Hepatoprotective Properties from the Extract and Fractions of Annona senegalensis Pers (Annonaceae) Stem Bark Grown in Nigeria. *European J Med Plants*. 2019:1-13.

39. Ajaiyeoba, E., Falade, M., Ogbole, O., Okpako, L., & Akinboye D. In vivo antimalarial and cytotoxic properties of Annona senegalensis extract. *African J Tradit Complement Altern Med*. 2006:3(1), 137-141.

40. Ngbolua, K. N., Mudogo, V., Mpiana, P. T., Tshibangu, D. S. T., Tshilanda, D. D., & Masengo CA. In vitro and in vivo anti-malarial and cytotoxic activities of ethanolic extracts of Annona senegalensis Pers (Annonaceae) from Democratic Republic of the Congo. *Jounal Mod Drug Discov Drug Deliv Res*. 2014:2(2).

41. Adzu, B., Abubakar, M. S., Izebe, K. S., Akumka, D. D., & Gamaniel KS. Effect of Annona senegalensis rootbark extracts on Naja nigricotlis nigricotlis venom in rats. *J Ethnopharmacol*. 2005:96(3), 507-513.

42. Zirihi, G. N., Mambu, L., Guédé-Guina, F., Bodo, B., & Grellier P. In vitro antiplasmodial activity and cytotoxicity of 33 West African plants used for treatment of malaria. *J Ethnopharmacol*. 2005:98(3), 281-285.

43. Jagadeesh. K, Srinivas. K SPR. Anti Inflammatory Effect of Azadirachta Indica (Neem) In Albino Rats-An Experimental Study. *IOSR J Pharm*. 2014;(January; 1):Pp 34-38.

44. Oseni, L. A., & Akwetey GM. An in-vivo evaluation of antiplasmodial activity of aqueous and ethanolic leaf extracts of Azadirachta indica in Plasmodium berghei infected balb/c mice. *Int J Pharm Sci Res*. 2012:3(5), 1406-1410.

45. Olela, B., Mbaria, J., Wachira, T., & Moriasi G. Acute Oral Toxicity and Anti-inflammatory and Analgesic Effects of Aqueous and Methanolic Stem Bark Extracts of Piliostigma thonningii (Schumach.). *Evidence-Based Complement Altern Med*. 2020.

46. Moriasi, G., Ireri, A., & Ngugi MP. In vitro antioxidant activities of the aqueous and methanolic stem bark extracts of Piliostigma thonningii (Schum.). *J evidence-based Integr Med*. 2020:25, 2515690X20937988.

47. Ibewuike, J. C., Ogungbamila, F. O., Ogundaini, A. O., Okeke, I. N., & Bohlin L. Antiinflammatory and antibacterial activities of C-methylflavonols from Piliostigma thonningii. *Phyther Res An Int J Devoted to Med Sci Res Plants Plant Prod*. 1997:11(4), 281-284.

48. Madara, A. A., Ajayi, J. A., Salawu, O. A., & Tijani AY. Anti-malarial activity of ethanolic leaf extract of Piliostigma thonningii Schum.(Caesalpiniacea) in mice infected with Plasmodium berghei berghei. *African J Biotechnol*. 2010:9(23), 3475-3480.

49. Kifle, Z. D., & Enyew EF. Evaluation of In Vivo Antidiabetic, In Vitro α-Amylase Inhibitory, and In Vitro Antioxidant Activity of Leaves Crude Extract and Solvent Fractions of Bersama abyssinica Fresen (Melianthaceae). *J Evidence-Based Integr Med*. 2020:25.

50. Zekeya N, Chacha M, Shahada F K, A. Analysis of phytochemical composition of Bersama abyssinica by gas chromatography - mass spectrometry. *J Pharmacogn Phytochem*. 2014:3(4):246-252.

51. Lather A, Gupta V, Tyagi V K V, S. G. Phytochemistry and pharmacological activities of Bersama engleriana Guerke – An overview. *Int Res J Pharmacy*. 2010:1(1): 89-94.

52. Mbah, C. C., Akuodor, G. C., Anyalewechi, N. A., Iwuanyanwu, T. C., & Osunkwo UA. In vivo antiplasmodial activities of aqueous extract of Bridelia ferruginea stem bark against Plasmodium berghei berghei in mice. *Pharm Biol*. 2012:50(2), 188-194.

53. Oloyede, O. I., & Babalola SO. IN VITRO ANTIOXIDANT ACTIVITY OF ETHANOLIC EXTRACT OF BRIDELIA FERRUGINEA (STEM BARK). *Acad Res Int*. 2012:2(3), 246.

54. Shittu, O., Opeyemi, O. A., Salawu, M. K., Ashiru, A. A., Medaiyese, S. A., Asogwa, N., ... & Ajibaye O. Alterations in histological, biochemical and hematological parameters in Plasmodium berghei NK-65 infected balb/c mice treated with Bridelia ferruginea stem bark extract. *J Complement Integr Med 1(ahead-of-print)*. 2020.

55. Olarewaju, O. I., Oloyede, O. I., Ojo, O. A., & Onikanni SA. Effects of aqueous extract of Bridelia ferruginea stem bark on some haematological parameters of albino rats. *Innov Pharm Pharmacother*. 2013:1(2), 70-75.

56. Kolawole, O. M., & Adesoye AA. Evaluation of the antimalarial activity of Bridelia ferruginea benth bark. *Can J Pure Appl Sci,*. 2010:4, 1039-1044.

57. Olajide, O. A., Okpako, D. T., & Makinde JM. Anti-inflammatory properties of Bridelia ferruginea stem bark: Inhibition of lipopolysaccaride-induced septic shock and vascular permeability. *J Ethnopharmacol*. 2003:88(2-3), 221-224.

58. Hassan, E. M., Matloub, A. A., Aboutabl, M. E., Ibrahim, N. A., & Mohamed SM. Assessment of anti-inflammatory, antinociceptive, immunomodulatory, and antioxidant activities of Cajanus cajan L. seeds cultivated in Egypt and its phytochemical composition. *Pharm Biol*. 2016:54(8), 1380-1391.

59. Wu, N., Fu, K., Fu, Y. J., Zu, Y. G., Chang, F. R., Chen, Y. H., ... & Gu CB. Antioxidant activities of extracts and main components of pigeonpea [Cajanus cajan (L.) Millsp.] leaves. *Molecules*. 2009:14(3), 1032-1043.

60. Ajaiyeoba, E. O., Ogbole, O. O., Abiodun, O. O., Ashidi, J. S., Houghton, P. J., & Wright CW. Cajachalcone: An antimalarial compound from Cajanus cajan leaf extract. *J Parasitol Res*. 2013.

61. Ayusman, S., Duraivadivel, P., Gowtham, H. G., Sharma, S., & Hariprasad P. Bioactive constituents, vitamin analysis, antioxidant capacity and α-glucosidase inhibition of Canna indica L. rhizome extracts. *Food Biosci*. 2020:35, 100544.

62. Al-Snafi AE. Therapeutic properties of medicinal plants: a review of their immunological effects (part 1). *Asian J Pharm Res*. 2015:5(3), 208-216.

63. Ménan, H., Banzouzi, J. T., Hocquette, A., Pélissier, Y., Blache, Y., Koné, M., ... & Valentin A. Antiplasmodial activity and cytotoxicity of plants used in West African traditional medicine for the treatment of malaria. *J Ethnopharmacol*. 2006:105(1-2), 131-136.

64. Habte, G., & Assefa S. In vivo antimalarial activity of crude fruit extract of Capsicum frutescens var. minima (Solanaceae) against Plasmodium berghei infected mice. 2020.

65. Gurnani, N., Gupta, M., Mehta, D., & Mehta BK. Chemical composition, total phenolic and flavonoid contents, and in vitro antimicrobial and

antioxidant activities of crude extracts from red chilli seeds (Capsicum frutescens L.). *J Taibah Univ Sci*. 2016:10(4), 462-470.

66. Takano F, Yamaguchi M, Takada S, Shoda S, Yahagi N, Takahashi T OT. Capsicum ethanol extracts and capsaicin enhance interleukin-2 and interferon-gamma production in cultured murine Peyer's patch cells ex vivo. *Life Sci*. 2007:80(17):1553–1563.

67. Bamidele V Owoyele, Olubori M Adebukola, Adeoye A Funmilayo AOS. Anti-inflammatory activities of ethanolic extract of Carica papaya leaves. *Inflammopharmacology*. 2008;(Aug):16(4):168-73.

68. Kovendan K, Murugan K, Panneerselvam C, et al. Antimalarial activity of Carica papaya (Family: Caricaceae) leaf extract against Plasmodium falciparum. *Asian Pacific J Trop Dis*. 2012;2(SUPPL.1):2, S306-S311. doi:10.1016/S2222-1808(12)60171-6

69. Imaga, N. A., Gbenle, G. O., Okochi, V. I., Adenekan, S., Duro-Emmanuel, T., Oyeniyi, B., ... & Ekeh FC. Phytochemical and antioxidant nutrient constituents of Carica papaya and Parquetina nigrescens extracts. *Sci Res Essays*. 2010:5(16), 2201-2205.

70. Noriko Otsuki, Nam H Dang, Emi Kumagai, Akira Kondo, Satoshi Iwata CM. Aqueous extract of Carica papaya leaves exhibits anti-tumor activity and immunomodulatory effects. *J Ethnopharmacol*. 2010;(Dec):2010 Feb 17;127(3):760-7.

71. Ntchapda, F., Barama, J., Azambou, D. R. K., Etet, P. F. S., & Dimo T. Diuretic and antioxidant activities of the aqueous extract of leaves of Cassia occidentalis (Linn.) in rats. *Asian Pacific J Trop Med*. 2015:8(9), 685-693.

72. Sreejith, G., Latha, P. G., Shine, V. J., Anuja, G. I., Suja, S. R., Sini, S., ... & Rajasekharan S. Anti-allergic, anti-inflammatory and anti-lipidperoxidant effects of Cassia occidentalis Linn. *Indian J Exp Biol*. 2010:48(5),494-8.

73. Al-Snafi AE. The therapeutic importance of Cassia occidentalis-An overview. *Indian J Pharm Sci Res*. 2015:5(3), 158-171.

74. Patel, N. K., Pulipaka, S., Dubey, S. P., & Bhutani KK. Pro-inflammatory cytokines and nitric oxide inhibitory constituents from Cassia occidentalis roots. *Nat Prod Commun*. 2014:9(5).

75. Jasso-Miranda, C., Herrera-Camacho, I., Flores-Mendoza, L. K., Dominguez, F., Vallejo-Ruiz, V., Sanchez-Burgos, G. G., ... & Reyes-Leyva J. Antiviral and immunomodulatory effects of polyphenols on macrophages infected with dengue virus serotypes 2 and 3 enhanced or not with antibodies. *Infect Drug Resist*. 2019:12, 1833.

76. Loganayaki, N., Siddhuraju, P., & Manian S. Antioxidant activity and free radical scavenging capacity of phenolic extracts from Helicteres isora L. and Ceiba pentandra L. *J Food Sci Technol*. 2013:50(4), 687-695.

77. Rao CRKR. Lipid Profiling by GC-MS and Anti-inflammatory Activities of Ceiba pentandra Seed Oil. *J Biol Act Prod from Nat*. 2014;(March):4(1):62-70.

78. Ezenyi, I. C., Salawu, O. A., Kulkarni, R., & Emeje M. Antiplasmodial activity-aided isolation and identification of quercetin-4'-methyl ether in Chromolaena odorata leaf fraction with high activity against chloroquine-resistant Plasmodium falciparum. *Parasitol Res*. 2014:113(12), 4415-4422.

79. Akinmoladun, A. C., Ibukun, E. O., Afor, E., Obuotor, E. M., & Farombi EO. Phytochemical constituent and antioxidant activity of extract from the leaves of Ocimum gratissimum. *Sci Res Essays*. 2007:2(5), 163-166.

80. Victor B. Owoyele JOA& AOS. Anti-inflammatory activity of aqueous leaf extract of Chromolaena odorata. *Inflammopharmacology*. 2005:479–484.

81. Thaddée Boudjeko, Rosette Megnekou, Alice Louise Woguia, Francine Mediesse Kegne, Judith Emery Kanemoto Ngomoyogoli, Christiane Danielle Nounga Tchapoum and OK. Antioxidant and immunomodulatory properties of polysaccharides from Allanblackia floribunda Oliv stem bark and Chromolaena odorata (L.) King and H.E. Robins leaves. *BMC Res Notes*. 2015:8: 759.

82. Mb U, Eo O, Aa F, Adamu Z, An A. Biomedical and Pharmaceutical Sciences Evaluation of the Antimalarial and Liver Function Potentials of Methanol Extract of Chrysophyllum albidum Stem Bark in Plasmodium berghei - Infected Mice. *J Biomed Pharm Sci*. 2018;1(1):1-5.

83. Adebayo, H. A., Abolaji, A. O., Kela, R., Ayepola, O. O., Olorunfemi, T. B., & Taiwo OS. Antioxidant activities of the leaves of Chrysophyllum Albidum G. *Pak J Pharm Sci*. 2011:24(4), 545-551.

84. Adedapo AMAVBBOOABB-AFOA; AD. Chrysophyllum albidum fruit peel attenuates nociceptive pain and inflammatory response in rodents by inhibition of pro-inflammatory cytokines and COX-2 expression through suppression of NF-κB activation. *Nutr Res*. 2020;77(May):73-84 Pages 73-84.

85. Ettebong, E., Ubulom, P., & Etuk A. Antiplasmodial activity of methanol leaf extract of Citrus aurantifolia (Christm) Swingle. *J Herbmed Pharmacol*. 2019:8(4), 274-280.

86. Al-Aamri, M. S., Al-Abousi, N. M., Al-Jabri, S. S., Alam, T., & Khan SA. Chemical composition and in-vitro antioxidant and antimicrobial activity of the essential oil of Citrus aurantifolia L. leaves grown in Eastern Oman. *J Taibah Univ Med Sci*. 2018:13(2), 108-112.

87. M Gharagozloo AG. Immunomodulatory effect of concentrated lime juice extract on activated human mononuclear cells. *J Ethnopharmacol*. 2001;(Sep):77(1):85-90.

88. Chun-Yan Shen, Jian-Guo Jiang, Wei Zhu and QO-Y. Anti-inflammatory Effect of Essential Oil from Citrus aurantium L. var. amara Engl. *J Agric Food Chem*. 2017:65, 39, 8586–8594.

89. Sarrou, E., Chatzopoulou, P., Dimassi-Theriou, K., & Therios I. Volatile constituents and antioxidant activity of peel, flowers and leaf oils of Citrus aurantium L. growing in Greece. *Molecules*. 2013:18(9), 10639-10647.

90. Sanei-Dehkordi, A., Sedaghat, M. M., Vatandoost, H., & Abai MR. Chemical compositions of the peel essential oil of Citrus aurantium and its natural larvicidal activity against the malaria vector Anopheles stephensi (Diptera: Culicidae) in comparison with Citrus paradisi. *J arthropod-borne Dis*. 2016:10(4), 577.

91. Diab K. In Vitro Studies on Phytochemical Content, Antioxidant, Anticancer, Immunomodulatory , and Antigenotoxic Activities of Lemon, Grapefruit, and Mandarin Citrus Peels. *Asian Pacific J cancer Prev APJCP*. 2016;(January):17(7):3559-3567.

92. Rafeeq Alam Khan NM and ZF. Anti-inflammatory effects of Citrus sinensis L., Citrus paradisi L. and their combinations. *Pak J Pharm Sci*. 2016;(May):29(3):843-852.

93. Ivoke, N., Ogbonna, P. C., Ekeh, F. N., Ezenwaji, N. E., Atama, C. I., Ejere, V. C., ... & Eyo JE. Effects of grapefruit (Citrus paradisi MACF) (Rutaceae) peel oil against developmental stages of Aedes aegypti (Diptera: Culicidae). *Southeast Asian J Trop Med Public Health*. 2013:44, 970-978.

94. Giamperi, L., Fraternale, D., Bucchini, A., & Ricci D. Antioxidant activity of Citrus paradisi seeds glyceric extract. *Fitoterapia*. 2004:75(2), 221-224.

95. Orabueze, C. I., Obi, E., Adesegun, S. A., & Coker HA. Potential antimalarial activity of Coccinia barteri leaf extract and solvent fractions against Plasmodium berghei infected mice. *J Ethnopharmacol*. 2020:248, 112334.

96. Hamid, A. A., Oguntoye, S. O., Mukadam, A. A., Zubair, A. O., Fagbohun, E. O., Adeyemo, J., ... & Olaniyi B. Chemical constituents, antimicrobial and antioxidant properties of the aerial parts of Coccinia barteri. *Chem Int*. 2017:3, 428-441.

97. Dakuyo, Z., Meda, A. L., Ollo, D., Kiendrebeogo, M., Traore-Coulibaly, M., Novak, J., ... & Willcox M. SAYE: The story of an antimalarial phytomedicine from Burkina Faso. *J Altern Complement Med*. 2015:21(4), 187-195.

98. Yerbanga, R. S., Lucantoni, L., Lupidi, G., Dori, G. U., Tepongning, N. R., Nikiéma, J. B., ... & Habluetzel A. Antimalarial plant remedies from Burkina Faso: their potential for prophylactic use. *J Ethnopharmacol*. 2012:140(2), 255-260.

99. Anaga, A. O., & Oparah N. Investigation of the methanol root extract of Cochlospermum planchonii for pharmacological activities in vitro and in vivo. *Pharm Biol*. 2009:47(11), 1027-1034.

100. Oumar, Y. S., Nathalie, G. K., Souleymane, M., Karamoko, O., Alexis, B. G., David, G. J., & Adama C. In vitro antioxidant activity of extracts of the root Cochlospermum planchonii Hook. f. ex. Planch (Cochlospermaceae). *J Pharmacog Phytochem*. 2014:3(4), 164-170.

101. Wright, C. W., Phillipson, J. D., Awe, S. O., Kirby, G. C., Warhurst, D. C., Quetin-Leclercq, J., & Angenot L. Antimalarial activity of cryptolepine and some other anhydronium bases. *Phyther Res*. 1996:10(4), 361-363.

102. Cimanga, K., De Bruyne, T., Pieters, L., Vlietinck, A. J., & Turger CA. In vitro and in vivo antiplasmodial activity of cryptolepine and related alkaloids from Cryptolepis sanguinolenta. *J Nat Prod*. 1997:60(7), 688-691.

103. Francine Mediesse Kengne, Kouamo Mangoua, Kenji Obadiah MS. Evaluation of in vitro antioxidant and immunomodulatory activities of polysaccharide fractions of Khaya grandifoliola C.D.C (Welw) stem bark and Cryptolepis sanguinolenta (Lindl.) Schltr leaves. *AFRICAN J Biotechnol*. 2018;(January).

104. Cimanga, K., Li, Y., De Bruyne, T., Apers, S., Cos, P., Bakana, P., ... & Vlietinck AJ. Inhibitors of xanthine oxidase and scavengers of superoxide anions from Cryptolepis sanguinolenta (Lindl.) Schlechter (Periplocaceae). *Pharm Pharmacol Commun*. 2000:6(7), 321-325.

105. Chinampudur V. Chandrasekaran, Kannan Sundarajan, Jothie R. Edwin, Giligar M. Gururaja, Deepak Mundkinajeddu and AA. Immune-stimulatory

and anti-inflammatory activities of Curcuma longa extract and its polysaccharide fraction. *Pharmacogn Res*. 2013;(Apr-Jun):5(2): 71–79.

106. Lwin, K. M., Mon, H. M., & Myint KH. Evaluation of the antimalarial activity of Curcuma longa Linn., singly and in combination with Eupatorium odoratum Linn. *J Ayurvedic Herb Med*. 2017:3(1), 11-14.

107. Singh, G., Kapoor, I. P. S., Singh, P., De Heluani, C. S., De Lampasona, M. P., & Catalan CA. Comparative study of chemical composition and antioxidant activity of fresh and dry rhizomes of turmeric (Curcuma longa Linn.). *Food Chem Toxicol*. 2010:48(4), 1026-1031.

108. Chukwuocha UM, Fernández-Rivera O, Legorreta-Herrera M. Exploring the antimalarial potential of whole Cymbopogon citratus plant therapy. *J Ethnopharmacol*. 2016;193:517-523. doi:10.1016/j.jep.2016.09.056

109. Jamuna, S., Sadullah, S., Ashokkumar, R., Shanmuganathan, G., & Mozhi SS. Potential antioxidant and cytoprotective effects of essential oil extracted from Cymbopogon citratus on OxLDL and H2O2 LDL induced Human Peripheral Blood Mononuclear Cells (PBMC). *Food Sci Hum Wellness*. 2017:6(2), 60-69.

110. Sforcin TFB and JM. Lemongrass and citral effect on cytokines production by murine macrophages. *J Ethnopharmacol*. Volume 137(Issue 1, 1 September):Pages 909-913.

111. Rita Garcia, Joao Pinto Ferreira, Gustavo Costa, Telmo Santos, Fabio Branco, Margarida Caramona, Rui de Carvalho, Augusto Manuel Dinis, Maria Teresa Batista MC-B and IVF. Evaluation of Anti-inflammatory and Analgesic Activities of Cymbopogon citratus In vivo-Polyphenols Contribution. *Res J Med Plants*,. 2015:9: 1-13.

112. Susithra, E., & Jayakumari S. Analgesic and anti-inflammatory activities of Dichrostachys cinerea (L.) Wight and Arn. *Drug Invent Today*. 2018:10(3).

113. Hurinanthan V. Immune modulatory effect of Dichrostachys cinerea, Carpobrotus dimidiatus, Capparis tomentosa and Leonotis leonurus. *(Doctoral Diss*. 2009. http://hdl.handle.net/10321/455.

114. B Adzu, S Amos, S Dzarma, I Muazzam KSG. Pharmacological evidence favouring the folkloric use of Diospyros mespiliformis Hochst in the relief of pain and fever. *J Ethnopharmacol*. 2002;(Oct):82(2-3):191-5.

115. Adzu, B., & Salawu OA. Screening Diospyros mespiliformis extract for antimalarial potency. *Int J Biol Chem Sci*. 2009:3(2).

116. Agbaje, E. O., & Onabanjo AO. The effects of extracts of Enantia chlorantha in malaria. *Ann Trop Med Parasitol*. 1991:85(6), 585-590.

117. Petrelli, R., Orsomando, G., Sorci, L., Maggi, F., Ranjbarian, F., Biapa Nya, P. C., ... & Bramucci M. Biological activities of the essential oil from Erigeron floribundus. *Molecules*. 2016:21(8), 1065.

118. Asongalem, E. A., Foyet, H. S., Ngogang, J., Folefoc, G. N., Dimo, T. H. É. O. P. H. I. L. E., & Kamtchouing PIERRE. Analgesic and antiinflammatory activities of Erigeron floribundus. Journal. *Ethnopharmacol*. 2004:91(2-3), 301-308.

119. Yapo, F. A., Yapi, F. H., Ahiboh, H., Hauhouot-Attounbre, M. L., Guédé, N. Z., Djaman, J. A., & Monnet D. Immunomodulatory effect of the aqueous extract of Erigeron floribundus (Kunth) Sch Beep (Asteraceae) Leaf in Rabbits. *Trop J Pharm Res*. 2011:10(2).

120. Zofou, D., Tene, M., Ngemenya, M. N., Tane, P., & Titanji VP. In vitro antiplasmodial activity and cytotoxicity of extracts of selected medicinal plants used by traditional healers of Western Cameroon. *Malar Res Treat*. 2011.

121. Méndez, G. L., Conde, C. G., & Alarcón MET. Extraction, description and antioxidant activity of essential oil from Eucalyptus globulus Labill. *Rev Cuba Farm*. 2019:52(1), 1-12.

122. Sadlon, A. E., & Lamson DW. Immune-modifying and antimicrobial effects of Eucalyptus oil and simple inhalation devices. *Altern Med Rev*. 2010:15(1), 33-43.

123. Ismail, A., Mohamed, M., Kwei, Y. F., & Yin KB. Euphorbia hirta methanolic extract displays potential antioxidant activity for the development of local natural products. *Pharmacognosy Res*. 2019:11(1), 78.

124. Asha, S., Thirunavukkarasu, P., Mani, V. M., & Sadiq AM. Antioxidant activity of Euphorbia hirta Linn leaves extracts. *European J Med Plants*. 2016:1-14.

125. Pratheepa, V., & Sukumaran N. Effect of Euphorbia hirta plant leaf extract on immunostimulant response of Aeromonas hydrophila infected Cyprinus carpio. *Peer J*. 2014:2, e671.

126. Ahmad, S. F., Attia, S. M., Bakheet, S. A., Ashour, A. E., Zoheir, K. M., & Abd-Allah AR. Anti-inflammatory effect of Euphorbia hirta in an adjuvant-induced arthritic murine model. *Immunol Invest*. 2014.

127. Chen, J., Er, H., Mohamed, S., & Chen Y. In vitro anti-inflammatory activity of fractionated Euphorbia hirta aqueous extract on rabbit synovial fibroblasts. *Biomed J*. 2015:38(4).

128. Daikwo OA, Tende JASMOEEDIA sherif. The Effect of Aqueous Extract of Leaf of Ficus capensis Thunb (Moraceae) on in Vivo Leukocyte Mobilization in Wistar Rats. *Br J Pharmacol Toxicol*. 2012;(June):3(3).

129. Muanda, N. F., Dicko, A., & Soulimani R. Chemical composition and biological activities of Ficus capensis leaves extracts. *J Nat Prod*. 2010:3(1), 147-160.

130. Akomolafe, S. F., Oboh, G., Oyeleye, S. I., & Boligon AA. Aqueous extract from Ficus capensis leaves inhibits key enzymes linked to erectile dysfunction and prevent oxidative stress in rats' penile tissue. *NFS J*. 2016:4, 15-21.

131. Chao, C. H., Cheng, J. C., Hwang, T. L., Shen, D. Y., & Wu TS. Trinorditerpenes from the roots of Flueggea virosa. *Bioorganic Med Chem Lett*. 2014:24(2), 447-449.

132. Kaou, A. M., Mahiou-Leddet, V., Hutter, S., Aïnouddine, S., Hassani, S., Yahaya, I., ... & Ollivier E. Antimalarial activity of crude extracts from nine

African medicinal plants. *J Ethnopharmacol*. 2008:116(1), 74-83.

133. Chauke, A. M., Shai, L. J., Mphahlele, P. M., & Mogale MA. Radical scavenging activity of selected medicinal plants from Limpopo province of South Africa. *African J Tradit Complement Altern Med*. 2012:9(3), 426-430.

134. S. Sabiu, E.O. Ajani, A.A. Ajao, T.O.Sunmonu, A.S. Ibraheem, R. Ibrahim, H. Mustapha AOA. Biomembrane stabilization and antiulcerogenic properties of aqueous leaf extract of Gossypium barbadense L. (Malvaceae) Autho. *Beni-Suef Univ J Basic Appl Sci*. 2017;Volume 6,(Issue 4, December,):Pages 301-309.

135. Ade-Ademilua, O. E., & Okpoma MO. Gossypium hirsutum L. and Gossypium barbadense L.: differences in phytochemical contents, antioxidant and antimicrobial properties. *Ife J Sci*. 2018:20(1), 77-88.

136. Salako, O. A., & Awodele O. Evaluation of the antimalarial activity of the aqueous leaf extract of Gossypium barbadense (Malvaeceae) in mice. *Drugs Ther Stud*. 2012:2(1), e2-e2.

137. Al-Snafi AE. Chemical constituents and pharmacological activities of Gossypium herbaceum and Gossypium hirsutum-A review. *IOSR J Pharm*. 2018:8(5), 64-80.

138. Benoit, F., Valentin, A., Pelissier, Y., Diafouka, F., Marion, C., Kone-Bamba, D., ... & Bastide JM. In vitro antimalarial activity of vegetal extracts used in West African traditional medicine. *Am J Trop Med Hyg*. 1996:54(1), 67-71.

139. Sahabi, S. M., & Abubakar SD. Bone Marrow Changes Induced by Guiera Senegalensis in Acetic Acid-induced Colitis in Wistar Rats. *Res Int Arch Curr*. 2018:1-8.

140. Jigam, A. A., Akanya, H. O., Dauda, B. E., & Ogbadoyi EO. Antiplasmodial, analgesic and anti-inflammatory effects of crude Guiera senegalensis Gmel (Combretaceae) leaf extracts in mice infected with Plasmodium berghei. *J Pharmacogn Phyther*. 2011:3(10), 150-154.

141. Parvez, M. K., Alam, P., Arbab, A. H., Al-Dosari, M. S., Alhowiriny, T. A., & Alqasoumi SI. Analysis of antioxidative and antiviral biomarkers β-amyrin, β-sitosterol, lupeol, ursolic acid in Guiera senegalensis leaves extract by validated HPTLC methods. *Saudi Pharm journal,*. 2018:26(5), 685-693.

142. Bouchet, N., Barrier, L., & Fauconneau B. Radical scavenging activity and antioxidant properties of tannins from Guiera senegalensis (Combretaceae). *Phyther Res An Int J Devoted to Pharmacol Toxicol Eval Nat Prod Deriv*. 1998:12(3), 159-162.

143. Gessler, M. C., Nkunya, M. H., Mwasumbi, L. B., Heinrich, M., & Tanner M. Screening Tanzanian medicinal plants for antimalarial activity. *Acta Trop*. 1994:56(1), 65-77.

144. Gessler, M. C., Tanner, M., Chollet, J., Nkunya, M. H. H., & Heinrich M. Tanzanian medicinal plants used traditionally for the treatment of malaria: in vivo antimalarial and in vitro cytotoxic activities. *Phyther Res*. 1995:9(7), 504-508.

145. Makgatho, M. E., Nxumalo, W., & Raphoko LA. Anti-mycobacterial,-oxidative,-proliferative and-inflammatory activities of dichloromethane leaf extracts of Gymnosporia senegalensis (Lam.) Loes. *South African J Bot*. 2018:114, 217-222.

146. Kpoyizoun, P. K., Metowogo, K., Kantati, Y. T., Missebukpo, A., Dare, T., Lawson-Evi, P., ... & Aklikokou KA. Antiinflammatory and antioxidant evaluation of Maytenus senegalensis hydroalcoholic roots extract fractions in allergic asthma. *J Phytopharm*. 2020:9(4), 252-257.

147. Boampong, J. N., Karikari, A. A., & Ameyaw EO. In vivo antiplasmodial and in vitro antioxidant properties of stem bark extracts of Haematostaphis barteri. *Asian Pac J Trop Biomed*. 2015:5(6), 446-450.

148. Medewase, J. O and Ezike AC. Preliminary investigation of the anti-inflammatory activity of Harungana madagascariensis leaf extract Lam.Expoir (Hypericaceae). *Acad Journals*. 2018;(30th May – 2nd June,).

149. Ndjakou Lenta B, Ngouela S, Fekam Boyom F, et al. Anti-plasmodial activity of some constituents of the root bark of Harungana madagascariensis LAM. (Hypericaceae). *Chem Pharm Bull*. 2007;55(3):464-467. doi:10.1248/cpb.55.464

150. Antia, B. S., Ita, B. N., & Udo UE. Nutrient composition and in vitro antioxidant properties of Harungana madagascariensis stem bark extracts. *J Med Food*. 2015:18(5), 609-614.

151. Santhosha, D., Ramesh, A., Hemalatha, E., & Nagulu M. Phytochemical screening and Antioxidant activity of ethanolic extract of Heliotropium indicum. *Int Res J Pharm*. 2015:6(8), 567-72.

152. Ashoka, M., Shasty, C. S., Sridevi, K., & Gopkumar P. Stimulation of immune function activity of the extract of Heliotropium indicum leaves. *Int J Pharmacol*. 2009:7(1).

153. Kyei, S., Koffuor, G. A., Ramkissoon, P., Ameyaw, E. O., & Asiamah EA. Anti-inflammatory effect of Heliotropium indicum Linn on lipopolysaccharide-induced uveitis in New Zealand white rabbits. *Int J Ophthalmol*. 2016:9(4), 528.

154. Ewere, E. G., Okolie, N. P., Etim, O. E., & Oyebadejo SA. Mitigation of Arsenic-induced Increases in Pro-Inflammatory Cytokines and Haematological Derangements by Ethanol Leaf Extract of Irvingia gabonensis. *Asian J Res Biochem*. 2020:36-47.

155. Ekpe, O.O., Nwaehujor, C.O., Ejiofor C.E., Arikpo, P.W., Woruji, E.E., & Amor ET. IRVINGIA GABONENSIS SEEDS EXTRACT FRACTIONATION, ITS ANTIOXIDANT ANALYSES AND EFFECTS ON RED BLOOD CELL MEMBRANE STABILITY. *Pharmacologyonline,*. 2019:1, 337-353.

156. Abdurahman, E. M., Abdulhakim A. & OGC. In-vitro Antioxidant Activities of Different Stem Bark Extracts of Irvingia gabonensis (Irvingiaceae). *Trop J Nat Prod Res*. 2020:4(6), 223-227.

157. Abiodun, O., Gbotosho, G., Ajaiyeoba, E., Happi, T., Falade, M., Wittlin, S., ... & Oduola A. In vitro antiplasmodial activity and toxicity assessment of some plants from Nigerian ethnomedicine. *Pharm Biol*. 2011:49(1), 9-14.

158. Airaodion, A. I., & Ogbuagu EO. Antiplasmodial Potential of Ethanolic Leaf Extract of Jatropha curcas against Plasmodium berghei. *Asian J Med Princ Clin Pract*. 2020:29-36.

159. Rofida S. Antioxidant activity of Jatropha curcas and Jatropha gossypifolia by DPPH method. *Pharm J Farm Indones (Pharmaceutical J Indones*. 2015:2(6), 281-284.

160. Abd-Alla, H. I., Moharram, F. A., Gaara, A. H., & El-Safty MM. Phytoconstituents of Jatropha curcas L. leaves and their immunomodulatory activity on humoral and cell-mediated immune response in chicks. *Zeitschrift für Naturforsch C,*. 2009:64(7-8), 495-501.

161. Mujumdar, A. M., & Misar A V. Anti-inflammatory activity of Jatropha curcas roots in mice and rats. *J Ethnopharmacol*. 2004:90(1), 11-15.

162. Onyegbule, F. A., Bruce, S. O., Onyekwe, O. N., Onyealisi, O. L., & Okoye PC. Evaluation of the in vivo antiplasmodial activity of ethanol leaf extract and fractions of Jatropha gossypifolia in Plasmodium berghei infected mice. *J Med Plants Res*. 2019:13(11), 269-279.

163. Nagaharika, Y., & Rasheed S. Anti-inflammatory activity of leaves of Jatropha gossypifolia L. by HRBC membrane stabilization method. *J Acute Dis*. 2013:2(2), 156-158.

164. Deo, S., Chaudhari, T., & Inam F. Study of immunomodulatory activity of naturally occurring and related synthetic 1-phenylnaphthalene lignans. *Int J Knowl Eng*. 2012:3(1).

165. Okoh, S. O., Iweriebor, B. C., Okoh, O. O., Nwodo, U. U., & Okoh AI. Antibacterial and antioxidant properties of the leaves and stem essential oils of Jatropha gossypifolia L. *Biomed Res Int*. 2016.

166. Gbeassor, M., Kossou, Y., Amegbo, K., De Souza, C., Koumaglo, K., & Denke A. Antimalarial effects of eight African medicinal plants. *J Ethnopharmacol*. 1989:25(1), 115-118.

167. Akshada, K., Madhuri, P., Kiran, W., Sandip, P., Magdum, C. S., & Naikwade NS. A comprehensive review of Jatropha gossypifolia Linn. *Pharmacogn Rev*. 2008:2(4 suppl.), 2-6.

168. Abiodun Falodun, CHING FIDELIS POH SAA. Phytochemical and anti inflammatory evaluation of Khaya grandifoliola stem bark extract. *Int J PharmTech Res*. 2009;(October):1(4).

169. Bickii, J., Njifutie, N., Foyere, J. A., Basco, L. K., & Ringwald P. In vitro antimalarial activity of limonoids from Khaya grandifoliola CDC (Meliaceae). *J Ethnopharmacol*. 2000:69(1), 27-33.

170. Njayou, F. N., Aboudi, E. C. E., Tandjang, M. K., Tchana, A. K., Ngadjui, B. T., & Moundipa PF. Hepatoprotective and antioxidant activities of stem bark extract of Khaya grandifoliola (Welw) CDC and Entada africana Guill. et Perr. *J Nat Prod*. 2013:6, 73-80.

171. Arthur, H., Joubert, E., De Beer, D., Malherbe, C. J., & Witthuhn RC. Phenylethanoid glycosides as major antioxidants in Lippia multiflora herbal infusion and their stability during steam pasteurisation of plant material. *Food Chem*. 2011:127(2), 581-588.

172. Abena, A. A., Diatewa, M., Gakosso, G., Gbeassor, M., Hondi-Assah, T. H., & Ouamba JM. Analgesic, antipyretic and anti-inflammatory effects of essential oil of Lippia multiflora. *Fitoterapia*. 2003:74(3), 231-236.

173. Tanveer Naved, Javed Inam Siddiqui, S. H. Ansari, Anis A. Ansari HMM. Immunomodulatory activity of Mangifera indica L. fruits (cv Neelam). *J Nat REMEDIES*. 2005;5/2:137-140.

174. Gabino Garrido, Deyarina González, Yeny Lemus, Dagmar García, Lizt Lodeiro, Gypsy Quintero, Carla Delporte, Alberto J Núñez-Sellés RD. In vivo and in vitro anti-inflammatory activity of Mangifera indica L. extract (VIMANG). *Pharmacol Res*. 2004;(Aug):50(2):143-9.

175. Malann, Y. D., Matur BM, & Akinnagbe ES. Antiplasmodial Activity of Extracts and Fractions of Mangifera Indica Against Plasmodium Berghei. *Niger J Parasitol*. 2014;35:1-7.

176. Martínez, G., Delgado, R., Pérez, G., Garrido, G., Núñez Sellés, A. J., & León OS. Evaluation of the in vitro antioxidant activity of Mangifera indica L. extract (Vimang). *Phyther Res An Int J Devoted to Pharmacol Toxicol Eval Nat Prod Deriv*. 2000:14(6), 424-427.

177. Akimanya, A., Midiwo, J. O., Okanga, F., Kerubo, L., Ilias, M., & Walker L. Two flavonoids and a rearranged clerodane diterpenoid from the leaf exudates of microglossa pyrifolia. *16th Symp Nat Prod reseach Netw East Cent Africa Arusha, Tanzania*. 2015;(August to September).

178. Akinpelu Lateef Abiola, Olawuni Idowu Julius, Ogundepo Gbenga Emmanuel, Adegoke Adesoji Mutiu OG and ITO. Spectroscopic analysis and anti-inflammatory effects of Milicia excelsa (Moraceae) leaf and fractions. *GSC Biol Pharm Sci*. 2019:06(03), 051–060.

179. Ayepola, O. O., Samson, A. F., & Onile-Ere O. In vitro Antioxidant and Anti-staphylococcal Activity of Bixa orellana Linn. and Milicia excelsa Welw. *J Complement Altern Med Res*. 2018:1-6.

180. Areola, J. O., Omisore, N. O., & Babalola OO. Antiplasmodial activity of stem-bark extract of Milicia excelsa (welw.) C.c.berg against rodent malaria parasites (Plasmodium berghei) in mice. *Ife J Sci*. 2016;18(4):905-911-911.

181. Waako, P. J., Gumede, B., Smith, P., & Folb PI. The in vitro and in vivo antimalarial activity of Cardiospermum halicacabum L. and Momordica foetida Schumch. Et Thonn. *J Ethnopharmacol*. 2005:99(1), 137-143.

182. P. Watcho, M.M. Donfack, F. Zelefack, T.B. Nguelefack, S.L. Wansi, F. Ngoula, P. Kamtchouing, E. Tsamo AK. Effects of the hexane extract of Mondia whitei on the reproductive organs of male rat. *African J Tradit Complement Altern Med*. 2005:302-311.

183. Owolabi, A. A., Fadare, D. A., Ogbole, O. O., & Ajaiyeoba EO. In vivo antimalarial activity of methanol extracts and fractions of Brachystegia eurycoma and Mondia whiteion chloroquine-resistant Plasmodium berghei. *Niger J Nat Prod Med*. 2019:23(1), 13-21.

184. Fred-Jaiyesimi AA, Ogunjobi OF. Antiepileptic activities of the extract and fractions of Mondia whitei (Hook f.) Skeel leaves. *Pharmacogn J*. 2013;5(6):256-258. doi:10.1016/j.phcgj.2013.10.004

185. Frederick Ayertey, Ebenezer Ofori-Attah, Stephen Antwi, Michael Amoa-Bosompem, Georgina Djameh, Nathaniel Lartey Lartey, Mistuko Ohashi,

Kwadwo Asamoah Kusi, Alfred Ampomah Appiah , Regina Appiah-Opong LKO. Anti-inflammatory activity and mechanism of action of ethanolic leaf extract of Morinda lucida Benth. *J Tradit Complement Med*. 2020;(4 August).

186. Okoh, S. O., Asekun, O. T., Familoni, O. B., & Afolayan AJ. Composition and antioxidant activities of leaf and root volatile oils of Morinda lucida. *Nat Prod Commun*. 2011:6(10).

187. Kipré, G. R., Bagré, I., Silué, K. D., Bla, K. B., Philippe, G., & Allico JD. Evaluation of Antiplasmodial and Antifungal Activity of Morinda morindoides (Baker) Milne-Redh (Rubiaceae), an Ivorian Traditional Medicinal Plant. International. *J Pharmacogn Phytochem Res*. 2015:7(2), 294-297.

188. Lucien, B. G., Calixte, B., Adrien, K. M., Joseph, D. A., & David N. In vitro and In vivo Antioxidant Activity of the Total Dichloromethane-ethanol Extract of Morinda morindoides (Baker) Milne-redh.(ETDE)(Rubiaceae). *Int J Biochem Res Rev*. 2015:7(4), 182-191.

189. Mohammed, A., Tam, D. N. H., Vu, T. L. H., Tieu, T. M., Elfaituri, M. K., Trinh, N. N., ... & Mizukami S. Morinda morindoides: A systematic review of its therapeutic activities. *South African J Bot*. 2020:131, 93-103.

190. Xu, Y. B., Chen, G. L., & Guo MQ. Antioxidant and anti-inflammatory activities of the crude extracts of Moringa oleifera from Kenya and their correlations with flavonoids. *Antioxidants*. 2019:8(8), 296.

191. Mulisa, E., Girma, B., Tesema, S., Yohannes, M., Zemene, E., & Amelo W. Evaluation of in vivo antimalarial activities of leaves of Moringa oleifera against Plasmodium berghei in Mice. *Jundishapur J Nat Pharm Prod*. 2018:13(1), 1-5.

192. Gupta, A., Gautam, M. K., Singh, R. K., Kumar, M. V., Rao, C. V., Goel, R. K., & Anupurba S. Immunomodulatory effect of Moringa oleifera Lam. extract on cyclophosphamide induced toxicity in mice. 2010.

193. Franklyn Nonso Iheagwam, Emmanuel Nsedu Israel, Kazeem Oyindamola Kayode, Opeyemi Christianah DeCampos, Olubanke Olujoke Ogunlana SNC. Nauclea latifolia Sm. Leaf Extracts Extenuates Free Radicals, Inflammation, and Diabetes-Linked Enzymes. *Oxidative Med Cell Longevity*,. 2020.

194. Oyedeji-Amusa, M. O., & Ashafa AOT. Medicinal properties of whole fruit extracts of Nauclea latifolia Smith.: Antimicrobial, antioxidant and hypoglycemic assessments. *South African J Bot*. 2019:121, 105-113.

195. Benoit-Vical, F., Valentin, A., Cournac, V., Pélissier, Y., Mallié, M., & Bastide JM. In vitro antiplasmodial activity of stem and root extracts of Nauclea latifolia SM (Rubiaceae). *J Ethnopharmacol*. 1998:61(3), 173-178.

196. Lusakibanza, M., Mesia, G., Tona, G., Karemere, S., Lukuka, A., Tits, M., ... & Frederich M. In vitro and in vivo antimalarial and cytotoxic activity of five plants used in congolese traditional medicine. *J Ethnopharmacol*. 2010:129(3), 398-402.

197. Mesia, K., Cimanga, K., Tona, L., Mampunza, M.M., Ntamabyaliro, N., Muanda, T., Muyembe, T., Totté, J., Mets, T., Pieters L. Assessment of the short-term safety and tolerability of a quantified 80% ethanol extract from the stem bark of Nauclea pobeguinii (PR 259 CT1) in healthy volunteers: a

clinical phase I study. *Planta Med*. 2011:77, 111–116.

198. Mesia, K., Tona, L., Mampunza, M.M., Ntamabyaliro, N., Muanda, T., Muyembe, T., Musuamba, T., Mets, T., Cimanga, K., Totté J. Antimalarial efficacy of a quantified extract of Nauclea pobeguinii stem bark in human adult volunteers with diagnosed uncomplicated falciparum malaria. Part 2: a clinical phase IIB trial. *Planta Med*. 2012:78, 853–860.

199. Y Tanko, G M Magaji, M Yerima, R A Magaji AM. Anti-nociceptive and anti-inflammatory activities of aqueous leaves extract of Ocimum gratissimum (Labiate) in rodents. *Afr J Tradit Complement Altern Med*. 2008;(Jan 22):5(2):141-6.

200. Santanu Kar Mahapatra, Subhankari Prasad Chakraborty and SR. Immunomodulatory Role of Ocimum gratissimum and Ascorbic Acid against Nicotine-Induced Murine Peritoneal Macrophages In Vitro. *Oxid Med Cell Longev*. 2011.

201. Kefe A, Giday M, Mamo H, Erko B. Antimalarial properties of crude extracts of seeds of Brucea antidysenterica and leaves of Ocimum lamiifolium. *BMC Complement Altern Med*. 2016;16(1):16(1), 118. doi:10.1186/s12906-016-1098-9

202. Ayoola, A. O., Akinloye, O., Oguntibeju, O. O., Oke, J. M., & Odetola AA. Antioxidant activities of Parquetina nigrescens. *African J Biotechnol*. 2011:10(24), 4920-4925.

203. Akinrinmade, F. J., Akinrinde, A. S., Soyemi, O. O., & Oyagbemi AA. Antioxidant potential of the methanol extract of Parquetina nigrescens mediates protection against intestinal ischemia-reperfusion injury in rats. *J Diet Suppl*. 2016:13(4), 420-432.

204. Owoyele, B. V., Nafiu, A. B., Oyewole, I. A., Oyewole, L. A., & Soladoye AO. Studies on the analgesic, anti-inflammatory and antipyretic effects of Parquetina nigrescens leaf extract. *J Ethnopharmacol*. 2009:122(1), 86-90.

205. Onyishi CK. Evaluation of In Vivo Anti-Malarial Activity of Methanolic Leaf Extract of Persea Americana against Plasmodium Berghei-in Mice. *IDOSR J Sci Res*. 2020:5(1) 44-52.

206. Adeyemi, O. O., Okpo, S. O., & Ogunti OO. Analgesic and anti-inflammatory effects of the aqueous extract of leaves of Persea americana Mill (Lauraceae). *Fitoterapia*. 2002:73(5), 375-380.

207. Báez-Magaña, M., Ochoa-Zarzosa, A., Alva-Murillo, N., Salgado-Garciglia, R., & López-Meza JE. Lipid-Rich Extract from Mexican Avocado Seed (Persea americana var. drymifolia) Reduces Staphylococcus aureus Internalization and Regulates Innate Immune Response in Bovine Mammary Epithelial Cells. *J Immunol Res*. 2019.

208. Kosińska, A., Karamać, M., Estrella, I., Hernández, T., Bartolomé, B., & Dykes GA. Phenolic compound profiles and antioxidant capacity of Persea americana Mill. peels and seeds of two varieties. *J Agric Food Chem*. 2012:60(18), 4613-4619.

209. Komlaga, G., Cojean, S., Beniddir, M. A., & Loiseau PM. The antimalarial potential of three Ghanaian medicinal plants. 2015.

210. Chinedu, E., Arome, D., & Ameh S. African herbal plants used as anti-malarial agents-A review. *PharmaTutor,*. 2014:2(3), 47-53.

211. Ojezele, M. O., Moke, E. G., & Onyesom I. Impact of generic antimalarial or Phyllanthus amarus and vitamin co-administration on antioxidant status of experimental mice infested with Plasmodium berghei. *Beni-Suef Univ J basic Appl Sci*. 2017:6(3), 260-265.

212. Olabiyi, F. A., Aboua, Y. G., Popoola, O. K., Monsees, T. K., & Oguntibeju OO. Evaluation of Antioxidant, Antityrosinase Activities and Cytotoxic Effects of Phyllanthus amarus Extracts. *Nat Prod J*. 2020:10(2),.

213. Wu, W., Li, Y., Jiao, Z., Zhang, L., Wang, X., & Qin R. Phyllanthin and hypophyllanthin from Phyllanthus amarus ameliorates immune-inflammatory response in ovalbumin-induced asthma: role of IgE, Nrf2, iNOs, TNF-α, and IL's. *Immunopharmacol Immunotoxicol*. 2019:41(1), 55-67.

214. Harikrishnan, H., Jantan, I., Alagan, A., & Haque MA. Modulation of cell signaling pathways by Phyllanthus amarus and its major constituents: potential role in the prevention and treatment of inflammation and cancer. *Inflammopharmacology*. 2020:1-18.

215. Ukwubile, Cletus Anes; IEO. Analgesic and Anti-inflammatory Activity of Physalis angulata Linn. (Solanaceae) Leaf Methanolic Extract in Swiss Albino Mice. *IBBJ*. Vol 2.

216. Y S Lin, H C Chiang, W S Kan, E Hone, S J Shih MHW. Immunomodulatory activity of various fractions derived from Physalis angulata L extract. *Am J Chin Med*. 1992:20(3-4):233-43.

217. Stoilova, I., Krastanov, A., Stoyanova, A., Denev, P., & Gargova S. Antioxidant activity of a ginger extract (Zingiber officinale). *Food Chem*. 2007:102(3), 764-770.

218. Biruksew, A., Zeynudin, A., Alemu, Y., Golassa, L., Yohannes, M., Debella, A., ... & Suleman S. Zingiber Officinale Roscoe and Echinops Kebericho Mesfin showed antiplasmodial activities against Plasmodium berghei in a dosedependent manner in Ethiopia. *Ethiop J Health Sci*. 2018:28(5).

219. Teugwa, C. M., Mejiato, P. C., Zofou, D., Tchinda, B. T., & Boyom FF. Antioxidant and antidiabetic profiles of two African medicinal plants: Picralima nitida (Apocynaceae) and Sonchus oleraceus (Asteraceae). *BMC Complement Altern Med*. 2013:13(1), 175.

220. Duwiejua, M., Woode, E., & Obiri DD. Pseudo-akuammigine, an alkaloid from Picralima nitida seeds, has anti-inflammatory and analgesic actions in rats. *J Ethnopharmacol*. 2002:81(1), 73-79.

221. Kiraithe, M. N., Nguta, J. M., Mbaria, J. M., & Kiama SG. Evaluation of the use of Ocimum suave Willd.(Lamiaceae), Plectranthus barbatus Andrews (Lamiaceae) and Zanthoxylum chalybeum Engl.(Rutaceae) as antimalarial remedies in Kenyan folk medicine. *J Ethnopharmacol*. 2016:178, 266-271.

222. Kapewangolo, P., Hussein, A. A., & Meyer D. Inhibition of HIV-1 enzymes, antioxidant and anti-inflammatory activities of Plectranthus barbatus. *J Ethnopharmacol*. 2013:149(1), 184-190.

223. Kapewangolo, P., & Meyer D. Plectranthus barbatus; antioxidant, and other inhibitory responses against HIV/AIDS. In HIV/AIDS. *Acad Press*. 2018:149-159.

224. Mi Jang, Seung-Weon Jeong, Somi K Cho, Kwang Seok Ahn, Jong Hyun Lee, Deok Chun Yang J-CK. Anti-inflammatory effects of an ethanolic

extract of guava (Psidium guajava L.) leaves in vitro and in vivo. *J Med Food*. 2014;(Jun):17(6):678-85.

225. Master David, T. Jawahar Abraham TSN& HA. Immunomodulatory effect of Guavarine®, aqueous guava leaf extract, on ornamental Koi carp Cyprinus carpio var. koi L. 1758. *J Appl Aquac*. 2017;Volume 29(Issue 3-4).

226. Chen, H. Y., & Yen GC. Antioxidant activity and free radical-scavenging capacity of extracts from guava (Psidium guajava L.) leaves. *Food Chem*. 2007:101(2), 686-694.

227. Malacrida, C., & Jorge N. Fatty acids and some antioxidant compounds of Psidium guajava seed oil. *Acta Aliment*. 2013:42(3), 371-378.

228. Simon, J. E., Wang, M., Gbewonyo, K., Rafi, M. M., Acquaye, D. F., & Asianowa Y. U.S. Patent No. 7,371,413. *Washington, DC US Pat Trademark Off*. 2008.

229. Leonard EC SD. Uses of vegetable butter-based cetyl myristoleate for treating osteoarthritis and other musculoskeletal disease conditions and injuries. *InUSA,*. 2010.

230. Momoh, J., Aina, O. O., Akoro, S. M., Ajibaye, O., & Okoh HI. In Vivo Anti-Plasmodial Activity and the Effect of Ethanolic Leaf Extract of Rauvolfia Vomitoria on hematological and Lipid Parameters in Swiss Mice Infected with Plasmodium Berghei NK 65. *J Home*. 2014:35, 1-2.

231. Erasto, P., Lubschagne, A., Mbwambo, Z. H., Nondo, R. S., & Lall N. Antimycobacterial, antioxidant activity and toxicity of extracts from the roots of Rauvolfia vomitoria and R. caffra. 2011.

232. Guanqun Zhan, Rongkun Miao, Fuxin Zhang, Yi Hao, Yu Zhang, Ying xu Zhang, Muhammad Khurm, Xinxin Zhang ZG. Peraksine derivatives with potential anti-inflammatory activities from the stems of Rauvolfia vomitoria. *Fitoterapia*. 2020;Volume 146(October):104704.

233. Peter M. Abuja, Michael Murkovic and WP. Antioxidant and Prooxidant Activities of Elderberry (Sambucus nigra) Extract in Low-Density Lipoprotein Oxidation. *J Agric Food Chem*. 1998:46, 10, 4091–4096.

234. Barak V, Halperin T KI. The effect of Sambucol, a black elderberry-based, natural product, on the production of human cytokines: I. Inflammatory cytokines. *Eur Cytokine Netw*. 2001;12(2)(Apr-Jun;):290-6.

235. Ojewole, J. A., Mawoza, T., Chiwororo, W. D., & Owira PM. Sclerocarya birrea (A. Rich) Hochst.[‘Marula’](Anacardiaceae): a review of its phytochemistry, pharmacology and toxicology and its ethnomedicinal uses. *Phyther Res An Int J Devoted to Pharmacol Toxicol Eval Nat Prod Deriv*. 2010:24(5), 633-639.

236. Armentano, M. F., Bisaccia, F., Miglionico, R., Russo, D., Nolfi, N., Carmosino, M., ... & Milella L. Antioxidant and proapoptotic activities of Sclerocarya birrea [(A. Rich.) Hochst.] methanolic root extract on the hepatocellular carcinoma cell line HepG2. *Biomed Res Int*. 2015.

237. Mariod, A. A., & Abdelwahab SI. Sclerocarya birrea (Marula), an African tree of nutritional and medicinal uses: a review. *Food Rev Int*. 2012:28(4), 375-388.

238. Nguta JM. In vivo antimalarial activity, toxicity, and phytochemical composition of total extracts from securidaca longepedunculata Fresen.(polygalaceae). *Biomed Biotechnol Res J*. 2019:3(3), 196.

239. Ottendorfer, D., Frevert, J., Kaufmann, R., Beuscher, N., Bodinet, C., Msonthi, J. D., ... & Hostettmann K. Enhancement of in vitro nonspecific immune functions by African plant extracts. *Phyther Res*. 1994:8(7), 383-390.

240. Adebesin, O. A., Okpuzor, J., Iroanya, O. O., Adenekan, S. O., & Aniekwena C. Antioxidant and cytotoxic properties of Senna alata and Senna podocarpa leaf extracts. *Planta Med*. 2013:79(13), PA27.

241. Olorunnisola, O. S., & Afolayan AJ. In vivo antioxidant and biochemical evaluation of Sphenocentrum jollyanum leaf extract in P. berghei infected mice. Pak. *J Pharm Sci*,. 2013:26(3), 445-450.

242. Kamagaté, M., Koffi, C., Kouamé, N. M., Akoubet, A., Alain, N., Yao, R., & Die HM. Ethnobotany, phytochemistry, pharmacology and toxicology profiles of Cassia siamea Lam. *J Phytopharm*. 2014:3(1), 57-76.

243. Kaur, G., Alam, M. S., Jabbar, Z., Javed, K., & Athar M. Evaluation of antioxidant activity of Cassia siamea flowers. *J Ethnopharmacol*. 2006:108(3), 340-348.

244. Ntandou, GF Nsonde, J. T. Banzouzi, B. Mbatchi, R. D. G. Elion-Itou, A. W. Etou-Ossibi, S. Ramos, F. Benoit-Vical, A. A. Abena and JMO. "Analgesic and anti-inflammatory effects of Cassia siamea Lam. stem bark extracts." *J Ethnopharmacol*. 2010:127, nos. 1 : 108-111.

245. Haddad, M. H. F., Mahbodfar, H., Zamani, Z., & Ramazani A. Antimalarial evaluation of selected medicinal plant extracts used in Iranian traditional medicine. *Iran J Basic Med Sci*. 2017:20(4), 415.

246. Loganayaki, N., Siddhuraju, P., & Manian S. Antioxidant activity of two traditional Indian vegetables: Solanum nigrum L. and Solanum torvum L. *Food Sci Biotechnol*. 2010:19(1), 121-127.

247. Pu, Y., Liu, Z., Zhong, C., Zhang, X., & Bao Y. Immunomodulatory effects of a polysaccharide from Solanum nigrum Linne through TLR4-MyD88 signaling pathway. *Int Immunopharmacol*. 2020:88, 106973.

248. Xiang, L., Wang, Y., Yi, X., & He X. Anti-inflammatory steroidal glycosides from the berries of Solanum nigrum L.(European black nightshade). *Phytochemistry*. 2018:148, 87-96.

249. Cho, H. D., Kim, J. H., Hong, S. M., Lee, J. H., Lee, Y. S., Kim, D. H., & Seo KI. Sorghum extract enhances caspase-dependent apoptosis in primary prostate cancer cells and immune activity in macrophages. *J Life Sci*. 2016:26(12), 1431-1437.

250. Benson, K. F., Beaman, J. L., Ou, B., Okubena, A., Okubena, O., & Jensen GS. West African Sorghum bicolor leaf sheaths have anti-inflammatory and immune-modulating properties in vitro. *J Med Food*. 2013:16(3), 230-238.

251. Awika, J. M., Rooney, L. W., Wu, X., Prior, R. L., & Cisneros-Zevallos L. Screening methods to measure antioxidant activity of sorghum (Sorghum

bicolor) and sorghum products. *J Agric food Chem*. 2003:51(23), 6657-6662.

252. Olorunnisola, O. S., & Afolayan AJ. In vivo anti-malaria activity of methanolic leaf and root extracts of Sphenocentrum jollyanum Pierre. *African J Pharm Pharmacol*. 2011:5(14), 1669-1673.

253. Moody, J. O., V. A. Robert, J. D. Connolly and PJH. "Anti-inflammatory activities of the methanol extracts and an isolated furanoditerpene constituent of Sphenocentrum jollyanum Pierre (Menispermaceae)." *J Ethnopharmacol*. 2006:104, nos. 1-2: 87-91.

254. Ndarubu, T.A., Rahinat G., Majiyebo, A..J., Julius I, N., Moshood A.O., Damola, S.A., & Eustace BB. Strychnos spinosa as a potential anti-oxidants and anti-microbials natural product. *Int J Med Sci*. 2020:2(1), 25-29.

255. Sadau, Y., & Eloff JN. In-vitro lipoxygenase inhibitory activity and total flavonoid of Strychnos spinosa leaf extracts and fractions. *Niger J Pharm Sci*. 2014:13(1).

256. Aravind, S. R., Joseph, M. M., Varghese, S., Balaram, P., & Sreelekha TT. Antitumor and immunopotentiating activity of polysaccharide PST001 isolated from the seed kernel of Tamarindus indica: an in vivo study in mice. *Sci World J*. 2012.

257. Siddhuraju P. Antioxidant activity of polyphenolic compounds extracted from defatted raw and dry heated Tamarindus indica seed coat. *LWT-Food Sci Technol*. 2007:40(6), 982-990.

258. Bhadoriya, S. S., Mishra, V., Raut, S., Ganeshpurkar, A., & Jain SK. Anti-inflammatory and antinociceptive activities of a hydroethanolic extract of Tamarindus indica leaves. *Sci Pharm*. 2012:80(3), 685-700.

259. Suralkar, A. A., Rodge, K. N., Kamble, R. D., & Maske KS. Evaluation of anti-inflammatory and analgesic activities of Tamarindus indica seeds. *Int J Pharm Sci Drug Res*. 2012:4(3), 213-217.

260. Abiodun, O. O., Rodríguez-Nogales, A., Algieri, F., Gomez-Caravaca, A. M., Segura-Carretero, A., Utrilla, M. P., ... & Galvez J. Antiinflammatory and immunomodulatory activity of an ethanolic extract from the stem bark of Terminalia catappa L.(Combretaceae): in vitro and in vivo evidences. *J Ethnopharmacol*. 2016:192, 309-319.

261. Chyau, C. C., Ko, P. T., & Mau JL. Antioxidant properties of aqueous extracts from Terminalia catappa leaves. *LWT-Food Sci Technol*. 2006:39(10), 1099-1108.

262. Ramiro-Puig, E., & Castell M. Cocoa: antioxidant and immunomodulator. *Br J Nutr*. 2009:101(7), 931-940.

263. Ejelonu, O. C., Elekofehinti, O. O., & Adanlawo IG. Tithonia diversifolia saponin-blood lipid interaction and its influence on immune system of normal wistar rats. *Biomed Pharmacother*. 2017:87, 589-595.

264. da Gama, R. M., Guimarães, M., de Abreu, L. C., & Armando-Junior J. Phytochemical screening and antioxidant activity of ethanol extract of Tithonia diversifolia (Hemsl) A. Gray dry flowers. *Asian Pac J Trop Biomed*. 2014:4(9), 740-742.

265. Goffin, E., Ziemons, E., De Mol, P., de Madureira, M. D. C., Martins, A. P., da Cunha, A. P., ... & Frederich M. In vitro antiplasmodial activity of Tithonia diversifolia and identification of its main active constituent: tagitinin C. *Planta Med*. 2002:68(6), 543-545.

266. Olanlokun, J. O., David, O. M., & Afolayan AJ. In vitro antiplasmodial activity and prophylactic potentials of extract and fractions of Trema orientalis (Linn.) stem bark. *BMC Complement Altern Med*. 2017:17(1), 407.

267. Uddin SN. Antioxidant and antibacterial activities of Trema orientalis Linn: an indigenous medicinal plant of indian subcontinent. *Orient Pharm Exp Med*. 2008:8(4), 395-9.

268. Oyebola, O. E., Morenikeji, O. A., & Ademola IO. In-vivo antimalarial activity of aqueous leaf and bark extracts of Trema orientalis against Plasmodium berghei in mice. *J Parasit Dis*. 2017:41(2), 398-404.

269. Jachak, S. M., Gautam, R., Selvam, C., Madhan, H., Srivastava, A., & Khan T. Anti-inflammatory, cyclooxygenase inhibitory and antioxidant activities of standardized extracts of Tridax procumbens L. *Fitoterapia*. 2011:82(2), 173-177.

270. Tiwari, U., Rastogi, B., Singh, P., Saraf, D. K., & Vyas SP. Immunomodulatory effects of aqueous extract of Tridax procumbens in experimental animals. *J Ethnopharmacol*. 2004:92(1), 113-119.

271. Nundkumar, N., & Ojewole JAO. Studies on the antiplasmodial properties of some South African medicinal plants used as antimalarial remedies in Zulu folk medicine. *Methods Find Exp Clin Pharmacol*. 2002:24(7), 397-402.

272. Abosi, A. O., Mbukwa, E., Majinda, R. R., Raseroka, B. H., Yenesew, A., Midiwo, J. O., ... & Waters NC. Vangueria infausta root bark: in vivo and in vitro antiplasmodial activity. *Br J Biomed Sci*. 2006:63(3), 129-133.

273. Kraft, C., Jenett-Siems, K., Siems, K., Jakupovic, J., Mavi, S., Bienzle, U., Eich E. In vitro antiplasmodial evaluation of medicinal plants from Zimbabwe. *Phytother Res*. 2003:17, 123–128.

274. Challand, S., Willcox M. A clinical trial of the traditional medicine Vernonia amygdalina in the treatment of uncomplicated malaria. *J Altern Compl Med*. 2009:15, 1231–1237.

275. Omoregie, E. S., & Pal A. Antiplasmodial, antioxidant and immunomodulatory activities of ethanol extract of Vernonia amygdalina del. Leaf in Swiss mice. *Avicenna J Phytomedicine*. 2016:6(2), 236.

276. Assefa, A., Urga, K., & Guta M. In vivo antimalarial activities of plants used in Ethiopian traditional medicine, Delomenna, Southeast Ethiopia. *Ethiop J Health Sci*. 2007:17(2), 81-90.

277. Agbafor, K. N., & Nwachukwu N. Phytochemical analysis and antioxidant property of leaf extracts of Vitex doniana and Mucuna pruriens. *Biochem Res Int*. 2011.

278. Dénou, A., Togola, A., Inngjerdingen, K. T., Zhang, B. Z., Ahmed, A., Dafam, D. G., ... & Paulsen BS. Immunomodulatory activities of

polysaccharides isolated from plants used as antimalarial in Mali. *J Pharmacogn Phyther*. 2019:11(2), 35-42.

279. Iwueke, A. V., Nwodo, O. F. C., & Okoli CO. Evaluation of the anti-inflammatory and analgesic activities of Vitex doniana leaves. *African J Biotechnol*. 2066:5(20), 1929.

280. Dikasso, D., Makonnen, E., Debella, A., Abebe, D., Urga, K., Makonnen, W., ... & Guta M. Anti-malarial activity of withania somnifera L. Dunal extracts in mice. *Ethiop Med J*. 2006:44(3), 279.

281. Yadava, S. A., Hakkim, L., Sathishkumar, F., & Sathishkumar R. Antioxidant activity of Withania somnifera (L.) Dunal by different solvent extraction methods. *J Pharm Res*. 2011:4(5), 1428-30.

282. Sivamani, S., Joseph, B., & Kar B. Anti-inflammatory activity of Withania somnifera leaf extract in stainless steel implant induced inflammation in adult zebrafish. *J Genet Eng Biotechnol*. 2014:12(1), 1-6.

283. Rasool, M., & Varalakshmi P. Immunomodulatory role of Withania somnifera root powder on experimental induced inflammation: An in vivo and in vitro study. *Vascul Pharmacol*. 2006:44(6), 406-410.

284. Shettar, A. K., Kotresha, K., Kaliwal, B. B., & Vedamurthy AB. Evaluation of in vitro antioxidant and anti-inflammatory activities of Ximenia americana extracts. *Asian Pacific J Trop Dis*. 2015:5(11), 918-923.

285. Olabissi, O. A. F., Moussa, O., Moustapha, O., Edgard, Z. F., Marius, L., & Pierre GI. Acute toxicity and anti-inflammatory activity of aqueous ethanol extract of root bark of Ximenia americana L.(Olacaceae). *African J Pharm Pharmacol*. 2011:5(7), 806-811.

286. Almeida, M. L. B., de Souza Freitas, W. E., de Morais, P. L. D., Sarmento, J. D. A., & Alves RE. Bioactive compounds and antioxidant potential fruit of Ximenia americana L. *Food Chem*. 2016:192, 1078-1082.

287. Macedo, T., Ribeiro, V., Oliveira, A. P., Pereira, D. M., Fernandes, F., Gomes, N. G., ... & Andrade PB. Anti-inflammatory properties of Xylopia aethiopica leaves: Interference with pro-inflammatory cytokines in THP-1-derived macrophages and flavonoid profiling. *J Ethnopharmacol*. 2020:248, 112312.

288. Boampong, J. N., Ameyaw, E. O., Aboagye, B., Asare, K., Kyei, S., Donfack, J. H., & Woode E. The curative and prophylactic effects of xylopic acid on Plasmodium berghei infection in mice. *J Parasitol Res*. 2013.

289. Chuks-Oguine, N. C., Bartimaeus, E. S., & Nwachuku EO. Evaluation of Antioxidant Potentials of Ocimum gratissimum and Xylopia aethiopica in Alcohol-Induced Hepatotoxic Albino Rats. *J Complement Altern Med Res*. 2020:8-16.

290. Obiri, D. D., & Osafo N. Aqueous ethanol extract of the fruit of Xylopia aethiopica (Annonaceae) exhibits anti-anaphylactic and anti-inflammatory actions in mice. *J Ethnopharmacol*. 2013:148(3), 940-945.

291. Tufts, H. R., Harris, C. S., Bukania, Z. N., & Johns T. Antioxidant and anti-inflammatory activities of kenyan leafy green vegetables, wild fruits, and

medicinal plants with potential relevance for kwashiorkor. *Evidence-Based Complement Altern Med*. 2015.

292. Matu, E. N., & Van Staden J. Antibacterial and anti-inflammatory activities of some plants used for medicinal purposes in Kenya. *J Ethnopharmacol*. 2003:87(1), 35-41.

298. Harokopakis, E., Albzreh, M. H., Haase, E. M., Scannapieco, F. A., & Hajishengallis, G. (2006). Inhibition of proinflammatory activities of major periodontal pathogens by aqueous extracts from elder flower (Sambucus nigra). *Journal of periodontology*, *77*(2), 271-279.

299. Gonzal, T. E., Flore, D. N. S., Donatien, A. A., William, N. Y., Herve, T. T., Vanessa, M. M. M., ... & Gilbert, A. (2020). In vitro anti-inflammatory, anti-oxidant and in vivo anti-arthritic properties of stem bark extracts from Nauclea pobeguinii (Rubiaceae) in rats. *Asian Pacific Journal of Tropical Biomedicine*, *10*(2), 65.

300. Acquaviva, R., Di Giacomo, C., Vanella, L., Santangelo, R., Sorrenti, V., Barbagallo, I., ... & Iauk, L. (2013). Antioxidant activity of extracts of Momordica foetida Schumach. et Thonn. *Molecules*, *18*(3), 3241-3249.

301. Gbadamosi, I. T., & Erinoso, S. M. (2015). In vitro antioxidant and antimicrobial activities of Mondia whitei (Hook. f.) skeels. *Journal of Basic and Applied Sciences*, *11*, 428-433.

302. Lin, Y. H., Hsiao, Y. H., Ng, K. L., Kuo, Y. H., Lim, Y. P., & Hsieh, W. T. (2020). Physalin A attenuates inflammation through down-regulating c-Jun NH2 kinase phosphorylation/Activator Protein 1 activation and up-regulating the antioxidant activity. *Toxicology and Applied Pharmacology*, *402*, 115115.

303. Olorunnisola, O. S., Adetutu, A., Balogun, E. A., & Afolayan, A. J. (2013). Ethnobotanical survey of medicinal plants used in the treatment of malarial in Ogbomoso, Southwest Nigeria. *Journal of Ethnopharmacology*, *150*(1), 71-78.

304. Ajaiyeoba, E. O., J. S. Ashidi, Larry Commander Okpako, P. J. Houghton, and Colin W. Wright. "Antiplasmodial compounds from Cassia siamea stem bark extract." *Phytotherapy Research: An International Journal Devoted to Pharmacological and Toxicological Evaluation of Natural Product Derivatives* 22, no. 2 (2008): 254-255.

305. Gwatidzo, L., Chowe, L., Musekiwa, C., & Mukaratirwa-Muchanyereyi, N. (2018). In vitro anti-inflammatory activity of Vangueria infausta: An edible wild fruit from Zimbabwe. *African Journal of Pharmacy and Pharmacology*, *12*(13), 168-175.
